# Thermodynamic framework to assess low abundance DNA mutation detection by hybridization

**DOI:** 10.1371/journal.pone.0177384

**Published:** 2017-05-25

**Authors:** Hanny Willems, An Jacobs, Wahyu Wijaya Hadiwikarta, Tom Venken, Dirk Valkenborg, Nadine Van Roy, Jo Vandesompele, Jef Hooyberghs

**Affiliations:** 1 Flemish Institute for Technological Research, VITO, Mol, Belgium; 2 Institute for Theoretical Physics, KULeuven, Leuven, Belgium; 3 Interuniversity Institute for Biostatistics and Statistical Bioinformatics, Hasselt University, Diepenbeek, Belgium; 4 Center for Medical Genetics Ghent (CMGG), Ghent University, Ghent, Belgium; 5 Cancer Research Institute Ghent (CRIG), Ghent University, Ghent, Belgium; 6 Theoretical Physics, Hasselt University, Diepenbeek, Belgium; Universite du Quebec a Trois-Rivieres, CANADA

## Abstract

The knowledge of genomic DNA variations in patient samples has a high and increasing value for human diagnostics in its broadest sense. Although many methods and sensors to detect or quantify these variations are available or under development, the number of underlying physico-chemical detection principles is limited. One of these principles is the hybridization of sample target DNA versus nucleic acid probes. We introduce a novel thermodynamics approach and develop a framework to exploit the specific detection capabilities of nucleic acid hybridization, using generic principles applicable to any platform. As a case study, we detect point mutations in the KRAS oncogene on a microarray platform. For the given platform and hybridization conditions, we demonstrate the multiplex detection capability of hybridization and assess the detection limit using thermodynamic considerations; DNA containing point mutations in a background of wild type sequences can be identified down to at least 1% relative concentration. In order to show the clinical relevance, the detection capabilities are confirmed on challenging formalin-fixed paraffin-embedded clinical tumor samples. This enzyme-free detection framework contains the accuracy and efficiency to screen for hundreds of mutations in a single run with many potential applications in molecular diagnostics and the field of personalised medicine.

## Introduction

The advent of whole-genome-sequencing has led to a tremendous increase in genetic loci that can be employed for molecular diagnostic tests. In particular, the detection of high-resolution genomic alterations, such as single-nucleotide variants (SNVs), are becoming progressively important in human diagnostics. For example, several known DNA mutations have been associated with clinical syndromes, disease prognosis or can be utilized to predict the response to therapy. Especially modern cancer therapies have a varying efficacy across a patient population, which in turn can be associated to the genomic variation. A case in point are mutations in the RAS oncogene family that predict patient response to EGFR-inhibiting drugs such as panitumumab (Vectibix) and cetuximab (Erbitux) [1]. Confident and swift detection of these single nucleotide substitutions are performed by different techniques [2–4], often non-sequencing molecular technologies as they can be miniaturized and easily adjusted for clinical practice. For this purpose, qPCR seems the most widely used technology in clinical laboratories (see also www.fda.gov/companiondiagnostics). The idea to use hybridization based technologies, e.g. microarrays, for mutation detection is not new [5], but it is rather uncommon. This observation seems somewhat surprising given the fact that hybridization technology is nowadays mature, affordable, widely used and versatile towards type and number of nucleic acid sequences to target for diagnostic tests. Moreover, hybridization as a system for SNV detection can be seen as a simple reversible and enzyme-free process. However, for a clear quantitative interpretation of microarray data, insight and use of the physico-chemical foundations of the hybridization process turn out to be crucial, especially because diagnostic applications impose specific constraints.

Competitive hybridization of different target sequences with the probe is the culprit that complicates the quantitative analysis. As proposed in previous work [6–8], hybridization of nucleic acid strands is a process that can be quantitatively modelled by the theory of thermodynamics. Physico-chemical modeling of nucleic acid hybridization enables the computation of free energies in pairs of single stranded target DNA and probe DNA after thermodynamic equilibrium has been reached. These free energies can in turn be used to differentiate between standard and non-standard Watson-Crick pairings for a particular probe-target interaction. This latter finding is especially powerful in human diagnostics where target DNA sequences will compete for hybridization at the set of probes.

In this work, we apply the theoretical concepts of DNA thermodynamics to present a quantitative hybridization-based technology to account for competitive hybridization, enabling a direct identification of SNV, and a framework to determine the detection limit. The data of this manuscript is produced on a microarray platform but the principles are platform-free and apply to any hybridization based sensor. To proof the concept of this new diagnostic tool, we apply our model to the measurement of point mutations in the KRAS oncogene, an important genetic marker for colorectal and lung cancer diagnostics and treatment stratification [9–11]. The technical challenges associated with this measurement are complicated because the set of relevant and approved biomarkers is changing over time and comprises a panel of markers instead of one specific mutation. Furthermore, the heterogeneity of the tissue material in the tumor is an unknown parameter (the ratio of cancer versus non-cancer cells in a clinical sample is variable and the DNA mutations can be heterozygous or homozygous origin) and requires a rigorous assessment of the detection limits. In addition, the tumor DNA is often of lower quality, since it is derived from formalin-fixed paraffin-embedded (FFPE) tissue. In order to illustrate that the hybridization-based technology is flexible and robust, and allows multiplexing of several mutations with a desired detection limit, we have designed three controlled sets of experiments:

Firstly, a typical clinical tumor sample has cancer cells (that contain the DNA mutation) present in minority. It is usually possible to take samples with more than 10% cancer cells but the ratio cancer/normal tissue is unknown prior the experimentation. The first aim of our study is the development of a method based on hybridization thermodynamics to assess the limit of detection with respect to the relative concentration of mutant DNA. For this purpose Experiment set 1 described in Materials and Methods is generated.

Secondly, in clinical DNA diagnostics, it is often needed to check for more than one possible mutation. In our case we focus on the twelve (mutually exclusive) possible point mutations in exon 2 of the KRAS gene and aim to assess each of them a single experiment. Hence we test the parallelization capability of the method as described in Experiment set 2.

Thirdly, clinical tumor tissue are often conserved as FFPE samples. As a consequence, DNA extracted from this FFPE material is of low quality due to cross-linking between nucleic acids and proteins and nucleic acid fragmentation. Therefore the third and obvious goal is to test real clinical FFPE tumor samples as described in Experiment set 3.

## Materials and methods

### Experimental setup

In the present study we define three sets of experiments, corresponding to the three goals mentioned in the introduction. The first two experimental sets consist of samples in which we use synthetic DNA (PCR amplified gBlocks, see sample preparation) and control the ratio of mutant over wild type. The third experimental set consists of DNA extracted from clinical tumor samples (see sample preparation).

1. **Experiment set 1** contains a dilution series of one mutation type, see Table 1, while keeping a constant total concentration of single stranded (ss) DNA.
2. **Experiment set 2** covers the 12 most commonly reported KRAS mutations, see Table 2, each at a relative mutant concentration of 5%. A wild type sample is used as negative control.
3. **Experiment set 3** consists of 7 clinical tumor samples, for which the mutant % is unknown. FFPE colon carcinoma samples from the Center of Medical Genetics Ghent, were analyzed in a blinded manner and compared with available sanger sequencing data. As a wild type reference the genomic DNA of a blood sample of a healthy individual was used.

**Table 1:** Dilution series for one mutation type.

relative DNA concentration of mutant 10C→G						
*c*_*mut*_/*c*_*total*_	0.10%	0.26%	0.64%	1.6%	4.0%	10%

Six synthetic samples in experiment set 1, containing mutation G12A (10C→G) with relative concentration as listed, dilution factor 2.5, total concentration of 5*nM*.

**Table 2:** Wild type and mutants sequences.

**Wild type sequence**		
	wild type	$5\prime -\ldots \text{CCTAC}\underset{13}{\underset{︸}{\text{G}\text{CC}}}\underset{12}{\underset{︸}{\text{A}\text{CC}}}\text{AGCTCC}\ldots -3\prime$
**Mutant sequences**		
Nucleotide notation	Amino acid notation	
9C→A	G12C	…GCCAC**A**…
9C→T	G12S	…GCCAC**T**…
9C→G	G12R	…GCCAC**G**…
10C→T	G12D	…GCCA**T**C…
10C→G	G12A	…GCCA**G**C…
10C→A	G12V	…GCCA**A**C…
12C→A	G13C	…GC**A**ACC…
12C→T	G13S	…GC**T**ACC…
12C→G	G13R	…GC**G**ACC…
13C→T	G13D	…G**T**CACC…
13C→G	G13A	…G**G**CACC…
13C→A	G13V	…G**A**CACC…

This table shows the 12 most commonly reported mutation types in exon 2 of the KRAS oncogene. All 12 are enclosed in experiment set 2 as gBlocks samples with a relative mutation amount of 5%. The first column shows the mutation types in nucleotide notation. The numbers 9 to 13 are merely nucleotide position counters of the probe region, they were introduced by us and are used in the Results section. The second column shows the amino acid notation: e.g. G12C is a mutation on codon 12 changing the wild type gly-G amino acid into cys-C. The third column shows the corresponding nucleotide sequence with the nucleotide mutation shown in bold.

### Probeset design

A microarray contains a number of predefined spots, which are local spaces on the array surface that contain many physically associated ssDNA copies of a given sequence (called probe sequence). An experiment begins with the hybridization of all the independent spots with a provided sample, i.e. an amount of the fluorescently labeled target ssDNA in solution. After hybridization and washing, the measurement consists of a determination of the fluorescent intensity *I* of each spot, which is proportional to the amount of target that hybridized to the corresponding probes. A probeset is the whole set of different probes which are designed and immobilized for a particular microarray experiment.

For our microarray experiments, we constructed a custom designed probeset as follows. Our study focuses on the region around codon 12 and 13 of exon 2 of the KRAS gene. From this sequence, the optimal probe length is derived: based on previous experimental experience [6] we estimated that a suited probe-target affinity would be achieved for probes of length 23 nucleotides. This results in the wild type target sequence of interest: 5’-GTTGGAGCTGGTGGCGTAGGCAA-3’. From this sequence, a probeset was designed (see Table 3). This probeset contains one perfectly matching (PM) probe against the target wild type. The rest of the probes contain all possible single or double mismatches (1MM or 2MM) against the wild type target, avoiding the free energy penalty coming from interaction between two mismatches and a mismatch located close to the edge of the helix structure [7]. From an experimental point of view: for each probe eight identical spots are present on the microarray and the median of the eight technical replicates is used as the hybridization signal *I* of the probe. The hybridization signal will be the highest for spots with a perfect matching sequencing, while the intensity will decrease depending on the number and kind of mismatch mutations.

**Table 3:** Wild type and mutants sequences.

**target**	
wild type	5’-TTGCCTACGCCACCAGCTCCAAC-3’
**probe**	
probe	3’-AACGGATGCGGTGGTCGAGGTTG-5’
1 MM	3’-AACG**A**ATGCGGTGGTCGAGGTTG-5’
	3’-AACG**T**ATGCGGTGGTCGAGGTTG-5’
	3’-AACG**C**ATGCGGTGGTCGAGGTTG-5’
	3’-AACGG**T**TGCGGTGGTCGAGGTTG-5’
	3’-AACGG**C**TGCGGTGGTCGAGGTTG-5’
	3’-AACGG**G**TGCGGTGGTCGAGGTTG-5’
	…
2 MM	3’-AACG**A**ATGC**A**GTGGTCGAGGTTG-5’
	3’-AACG**A**ATGC**T**GTGGTCGAGGTTG-5’
	3’-AACG**A**ATGC**C**GTGGTCGAGGTTG-5’
	3’-AACG**A**ATGCG**A**TGGTCGAGGTTG-5’
	3’-AACG**A**ATGCG**T**TGGTCGAGGTTG-5’
	3’-AACG**A**ATGCG**C**TGGTCGAGGTTG-5’
	…

This table sketches the probeset design. Probes contain zero, one or two mismatches against the wild type target. We did not introduce mismatches that are either too close to each other or near the boundaries to avoid extra penalty on the free energy (affinity) [7]. PM = perfect match, MM = mismatch

### Sample preparation and microarray experiments

To obtain synthetic ssDNA mixtures, a PCR reaction was performed on the double-stranded sequence-verified gBlocks Gene Fragments (IDT, Leuven, Belgium) which we will refer to as gBlocks. The PCR reaction comprised of 0.4 *μ*M forward (5’-GTCCTGCACCAGTAATATGC-3’), 0.4 *μ*M reverse (5’-CTGGCGTCATAGCTGTTTCCTGTGTGAGTATTAACCTTATG TGTGACA-3’) primers (Eurogentec, Seraing, Belgium), 2 mM MgSO4, 0.2 mM of each dNTP, 2 U Platinum Taq DNA High-Fidelity Polymerase (Life Technologies, Ghent, Belgium), and 0.5 ng gBlocks DNA in a final volume of 50 *μ*l. The reverse primer has a phosphate modification at the 5’ end. The DNA was amplified through 35 cyles (95°C, 30 s; 55°C, 30 s; 72°C, 30s) with a Veriti thermal cyler (Life Technologies). Amplicons were purified using Qiagen PCR purification kit (Qiagen, Hilden, Germany), according to manufacturer’s protocol. A lambda exonuclease treatment (Fermentas, St.Leon-Rot, Germany) was performed on the purified PCR product according to manufacturer’s protocol. Lambda exonuclease selectively digests the 5’-phosphorylated strand of double-stranded DNA. The obtained ssDNA was analysed on a FlashGel DNA system (Lonza, Slough, UK), and concentration was measured on a NanoDrop spectrophotometer.

For the clinical samples, the DNA was extracted using the Gentra Puregene Tissue Kit (Qiagen) according to manufacturer’s protocol. 100 ng DNA was used in the PCR reaction. Samples were also sequenced by Sanger sequencing for KRAS status in an ISO15189 accredited lab. No ethical approval was asked specifically for this study since the samples were send in for diagnostic purposes. The samples come from a biobank that are kept at the Center for Medical Genetics, Ghent. No URL is available since this is not a public biobank. All samples were obtained before the beginning of the study. No one of the authors were treating physicians nor had they any interaction with the subjects.

The microarray experiments of the first experiment set used a total ssDNA concentration of 5 nM, the second and third experimental set used 10 nM. The microarray experiments were performed using the commercially available Agilent G2565BA scanner system. A Cyanine-3 (Cy-3) labeled Barcode sequence was added to the hybridization mixture (Cy3-5’-AAAAACTGGCGTCATAGCTGTTTCCTGTGTGA-3’) to avoid direct labelling of the target sequences. The barcode was diluted in nuclease-free water to a final concentration of 0.05 *μ*M together with ssDNA (the concentration depends on the experiment), 5 *μ*l 10× blocking agent and 25 *μ*l 2× GEx hybridization buffer HI-RPM. The hybridization mixture was centrifuged at 13000 rpm for 1 minute and each microarray of the 8×15K custom Agilent slides was loaded with 40 *μ*l of the mixture. The hybridization occurred in an Agilent oven at 65°C for 17h with rotor setting 10 and the washing was performed according to the instructions of the manufacturer. Samples were analyzed using the Agilent Feature Extraction software (GE1 v5 95 Feb07), combining dynamic auto-focus intensity measurements with automatic background signal subtraction to improve the signal-to-noise ratio.

## Results

### Modelling of DNA thermodynamics

The clinical goal of this work is to differentiate samples containing only wild type (*wt*) DNA from samples consisting of a mixture of wild type with a minority of one of the mutant (*mut*) sequences, see Table 2. In the former case of a pure wild type sample, the microarray intensity *I* for each probe (microarray spot) can be modeled by the theory of the Langmuir isotherm [12–14]:

$$I_{wt}=Ac_{wt}e^{-\Delta G/RT}$$ (1)

where *A* is a device dependent optical proportionality factor, *R* the ideal gas constant, *T* the experimental temperature and most importantly Δ*G* the probe-target affinity. For a sample with a mix of wild type and mutant DNA, competitive hybridization to each probe occurs and leads to

$$\begin{array}{cc} I_{mix} & =I_{wt}+I_{mut}\\ & =A(c_{wt}e^{-\Delta G_{wt}/RT}+c_{mut}e^{-\Delta G_{mut}/RT}) \end{array}$$ (2)

where the free energies Δ*G*_*wt*_ and Δ*G*_*mut*_ are both with respect to the same probe under consideration.

**Fig 1:**
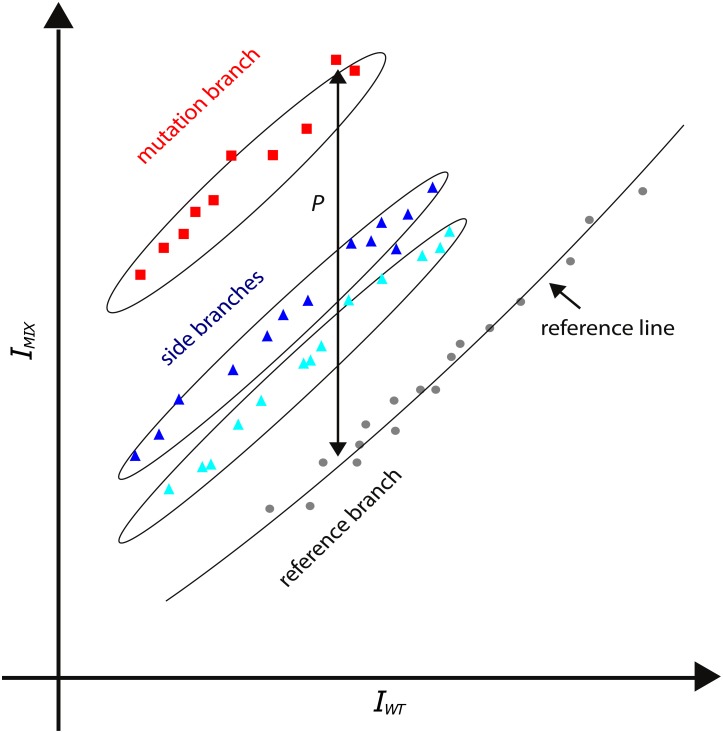
Representative scatter plot: The intensity values from a mixture sample containing a mutant in minority (*I*_*mix*_, y-axis) are plotted versus the intensity of a reference sample containing only wild type DNA (*I*_*wt*_, x-axis). A curve is drawn through the reference branch (black circles) using LOWESS smoothing and is called the reference line. The most deviating branch (mutation branch, red squares) contains information about the mismatching nucleotide of the mutant. Probes that do not match the nucleotide of the mutant but have nucleotide variations at the same position can be found in between (side branches). The distance *ρ* is the difference between the mutation and reference branch and is a visual representation of the change in free energy when a nucleotide mismatch mutation is introduced.

We apply this theory to extract sequence information from the intensity data since Δ*G* is sequence dependent. Hereto we perform one reference measurement with a known wild type sample and use this reference data in a scatter plot for each test sample. In these plots each point corresponds to one probe from the probeset (Table 3). When the test sample is also purely wild type, the resulting data points will lie on the diagonal identity line; when the sample is a wild type—mutant mix, the result will typically look like those presented in Fig 1. Since the mutant is present in minority, the contribution of the second term in Eq (2) will nearly always be negligible. This leads to a vast amount of data points on the identity diagonal (black circles in Fig 1). However, some of the probes will have a nucleotide variation that matches the mutant both in base position and type (Watson-Crick), which results in an increased affinity towards the mutant DNA, i.e. −ΔG_*mut*_ > −ΔG_*wt*_. These probes (red squares in Fig 1) will have an increased intensity. Finally there are probes with a nucleotide variation that matches the mutant in position but not in type (blue triangles in Fig 1). We name these three types of data points respectively the reference branch, the mutant branch and the side branch. More details on this branched structure can be found in Hadiwikarta *et al*. [15].

The information in this plot type leads to two practical consequences. Firstly, it provides quantitative information on the detection limit of a mutant in minority, secondly it can be used to perform hypothesis testing for presence of a mutant. The following two sections elaborate on this.

### Statistical testing for the presence of a mutant

To detect a mutation in a tumor sample, we need a statistical test to accept or reject each of the hypotheses on the presence of a specific mutation. By design we know the sequences of all microarray probes, each of the hypotheses can be associated with a subset of those probes which contain a specific mutation type and position. (Note that in our case the subsets will always contain more than one probe since the probeset of Table 3 includes probes with two mutations.) If the data points of a subset all fall in the reference branch, the hypothesis is falsified, i.e. this mutation is not present in the sample. Else, if the data points have an increased intensity over the reference branch and form the most deviating branch like the squares in Fig 1, this specific mutation type and position is present in the sample. Otherwise, if the data points are increased in intensity but are not the most deviating ones, like the triangles in Fig 1, the mutations position is confirmed but its base type hypothesis is falsified. For a practical implementation we perform locally weighted scatterplot smoothing (LOWESS) of the reference branch and calculate the vertical distance of all subset points to the reference line followed by a Wilcoxon rank-sum test. (In Fig 1 the LOWESS of the reference branch is indicated as a solid line called the reference line.) The Wilcoxon rank-sum is a conservative test, making very few assumptions about the data. There is room for statistical fine tuning, but the resulting *p*-values suffice for the present needs. In practice the *p*-values can be extremely small, therefore we present them in a logarithmic format throughout the manuscript and define *p*′ ≡ −ln(*p*), thus a high *p*′-value indicates a highly significant result.

### Analysis framework for detection limit

In order to screen for the presence of a mutant, the mutation branch needs to be detectable above the reference branch. Therefore a firm understanding of this relation is key to derive the detection limit of a mutant in minority. Hereto, we denote the logarithm of this distance as *ρ*, hence

$$\rho =ln\left(\frac{I_{mix}}{I_{wt}}\right)$$ (3)

From Eqs (1) and (2) one can derive the theoretical expression

$$\rho =\ln\left[1+\frac{c_{mut}}{c_{wt}}e^{-\Delta \Delta G/RT}\right]$$ (4)

where ΔΔG = ΔG_*mut*_ − ΔG_*wt*_. To first order, according to the nearest neighbor model for free energy of DNA, ΔΔ*G* is equal for all probes of the mutation branch and corresponds to the specific affinity penalty of the mismatching nucleotide of the mutant [7]. Moreover, in Eq (4) the device dependent factor *A* canceled out making *ρ* a purely physico-chemical parameter. Finally, one can rewrite the equation as

$$e^{\rho }-1=\frac{c_{mut}}{c_{wt}}e^{-\Delta \Delta G/RT}$$ (5)

In this expression we find a linear relation with the relative mutant abundance *c*_*mut*_/*c*_*wt*_, with intercept zero and a simple proportionality factor which is the physico-chemical impact of the nucleotide mutation *e*^−ΔΔ*G/RT*^. We will next show the validity of this relation experimentally and use it to derive a limit of detection for the KRAS mutations of Table 2. Note that although the theory predicts that the distance *ρ* is equal for all probes of the mutation branch, the experimental results will show some deviations between the data points. This is due to experimental conditions (not fully equilibrated system) and due to interaction effects of nucleotide mismatches [7]. In practice we are interested in *ρ* as a distance measure for the mutation branch as a whole, therefore in the rest of this paper we use the median value of all data points of the mutant branch and call this the distance *ρ* of the branch.

### Assessing the limit of detection for relative concentrations of mutant DNA

**Fig 2:**
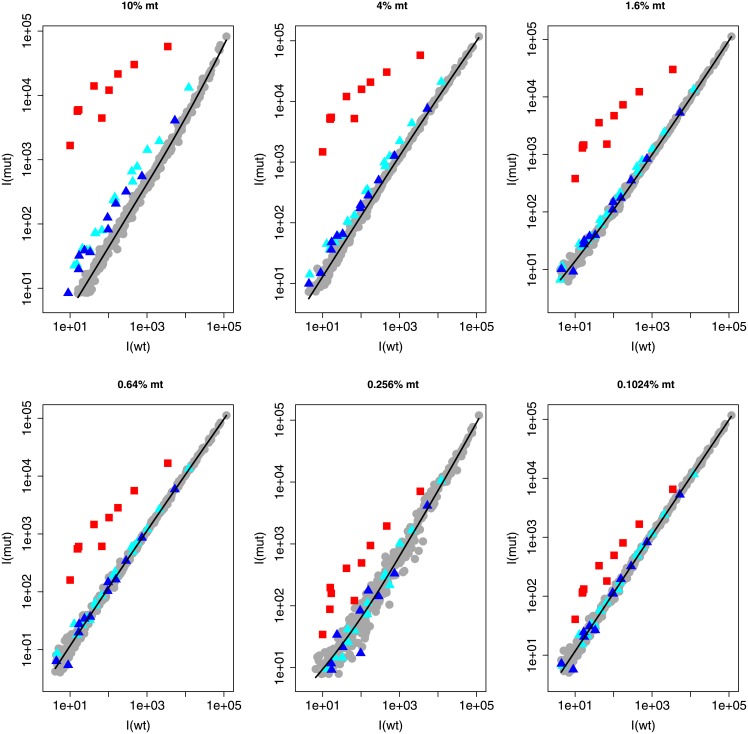
Scatter plot intensities of the 10C→G KRAS mutant using multiple relative concentrations: The intensity values from the mixed samples (wild type with 10C→G mutant; G12A in amino acid notation) are shown on the y-axis, while the intensity values of the wild type reference are shown on the x-axis. Data points below background intensity are removed. The plots show a decreasing relative mutant concentration, dilution factor 2.5. The relative concentration, *c*_*mut*_/*c*_*total*_, is indicated as a percentage on top of each graph.

**Fig 3:**
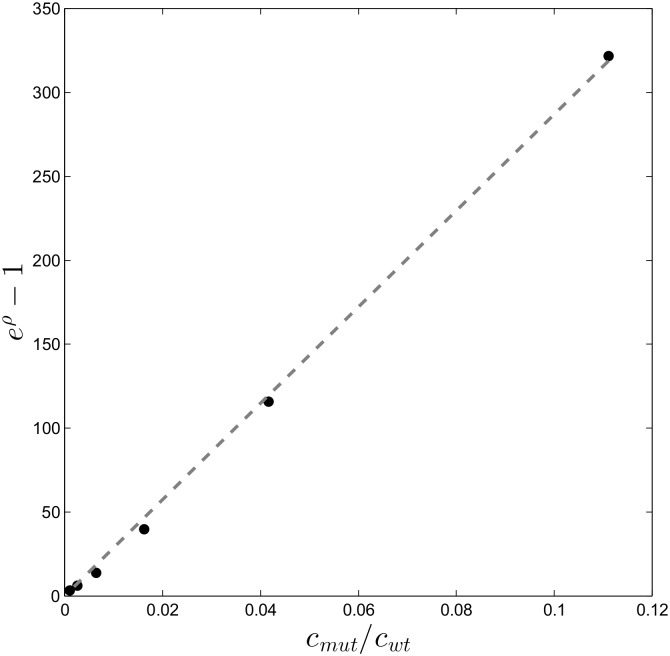
A concentration profile of 10C→G (G12A amino acid mutation) based on Eq 5. The dashed line is a linear fit with offset zero and a slope which corresponds to exp(−ΔΔ*G*/*RT*).

We next present experimental evidence that the vertical distance *ρ* between the mutation branch and the reference line is quantitatively related to the relative abundance of the mutant, indicated in %. In our hybridization model, this distance determines the detectability of a mutation, and the corresponding relative concentration *c*_*mut*_/*c*_*wt*_ is mutation dependent. In order to get an estimate of the detection limit for each mutation, we start by analyzing experiment set 1: the dilutions series of mutation 10C→G (or G12A in amino acid notation) presented in Table 1. The results of these six experiments are presented in Fig 2, which clearly show a deviating mutation branch in each subplot. We expect that the mutation branch distance relates to the relative concentration *c*_*mut*_/*c*_*wt*_ following Eq 5. The dilutions series experiment shows the validity of this relation as can be seen in Fig 3. The Figure confirms that the profile passes through the origin and is linear to a degree sufficient for our further analysis. Hence the profile is determined when a single measurement point is available.

**Fig 4:**
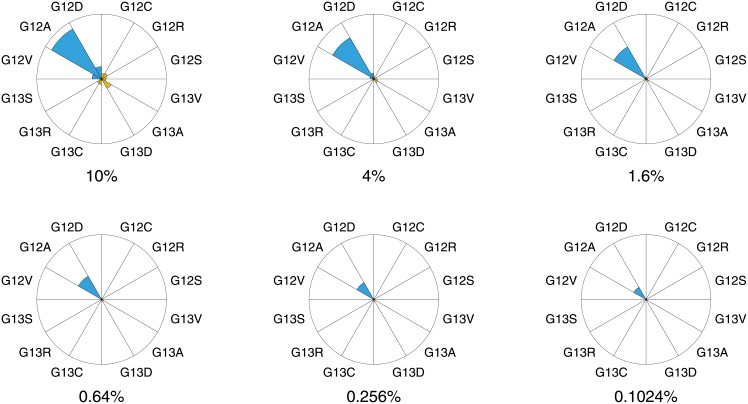
Pie diagrams of the statistical results of experiment set 1: Concentration range experiment with gBlocks samples. Each diagram corresponds to a relative concentration of the 10C→G mutant (G12A in amino acid notation) in the samples. The size of each pie is proportional to the vertical distance between the mutation branch and reference branch (*ρ*). The colour of each pie diagram represents the statistical significance: p-values below 0.01 are shown in blue while p-values above 0.01 are shown in orange. All values can be found in S1 Table for reference.

Next we apply the statistical test, described previously, on experiment set 1. For each sample, 12 mutations are possible: 3 on nucleotide position 9, 10, 12 and 13 (see Table 2). For each possible mutation the hypothesis is tested whether its corresponding mutation branch is deviating significantly from the reference branch, resulting in a *p*-value. The results are shown visually in Fig 4 and also a summary is provided in S1 Table for reference. For each sample a high statistical significance for a mutant hypothesis on position 10 is found. The associated point mutation leads to the correct identification of mutant 10C→G for each sample. Note that, at position 10, the mutation branch distance *ρ* is decreasing with decreasing mutant content as expected, but the statistical significance is untouched and remains invariant over the whole concentration range. Therefore, the statistical results do not deviate from the physical observations that we performed previously and this provides strong confidence in the method.

The concentration range results lead to two important conclusions. Firstly, the mutation 10C→G is still detectable at a relative concentration *c*_*mut*_/*c*_*total*_ = 0.1% (as confirmed by the statistical test). Secondly, the data in the 0.1% plot give a value of *ρ* ≈ 0.5, which we can subsequently use as the threshold *ρ*_*t*_ for detection. Hence, we can infer detection limits for other mutations by a measurement of *ρ* at a single concentration, which we do in the following section.

### Assessing the limit of detection for multiple mutations

**Fig 5:**
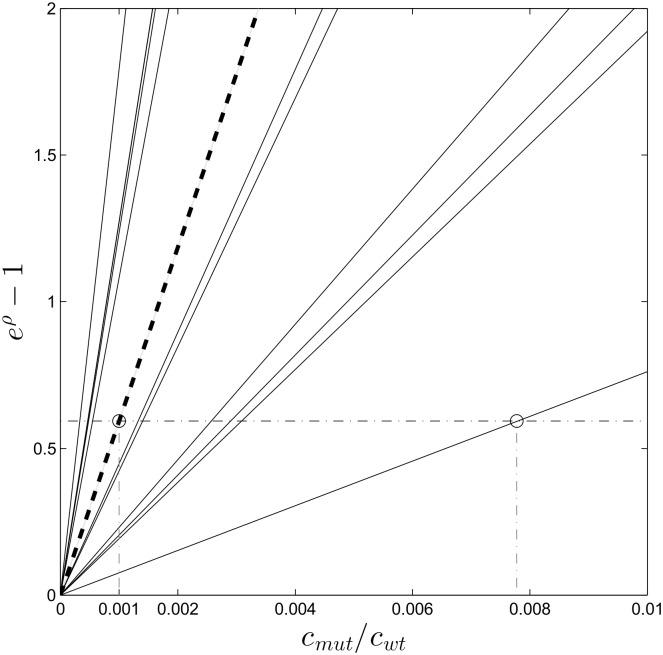
Plot of concentration profile similar to Fig 3 for all 12 KRAS mutations, based on experiment set 2. The profile is known to be linear, hence a single experiment per mutation type suffices. A higher slope corresponds to a stronger mutation. The bold dotted line is 10C→G (G12A in amino acid notation), the same mutation we used in Fig 3. The horizontal dashed line corresponds to the threshold value *ρ*_*t*_ ≈ 0.5 from which we can derive the concentration threshold for each mutation.

To check for more than one mutation in a hybridization experiment, we tested the 12 most commonly reported KRAS mutations at a relative concentration of 5% (see Table 2) and used a wild type sample as negative control. The result is shown in Fig 5, which clearly shows that the concentration profile that corresponds to the weakest ΔΔ*G* (which is the smallest slope) still intersects with the threshold at the point *c_mut_*/*c_wt_* ≈ 0.008 < 1%. This important result means that the detection limit of the weakest mutation is well below 1%.

**Fig 6:**
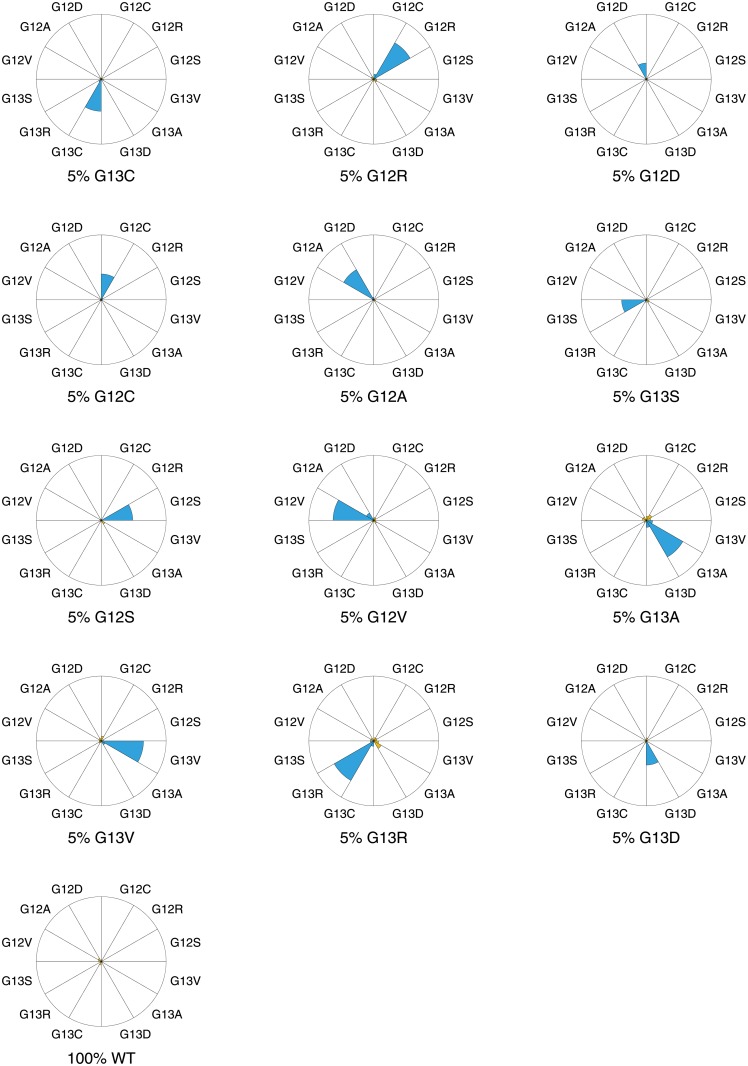
Pie diagrams of the statistical results of experiment set 2: 12 KRAS samples with each mutant present at 5% concentration. The size of each pie is proportional to the vertical distance between the mutation branch and reference branch (*ρ*). The colour of each pie diagram represents the statistical significance: p-values below 0.01 are shown in blue while p-values above 0.01 are shown in orange. All values can be found in S2 Table for reference.

We next confirm this result using statistical analysis, using the same approach we applied on experimental set 1 previously (Fig 4). As illustrated in Fig 6, each sample shows exactly one high statistical significance and for each sample the correct mutant is retrieved. The statistical results of the most significant nucleotide positions are also summarized in S2 Table.

### Application to clinical samples

**Fig 7:**
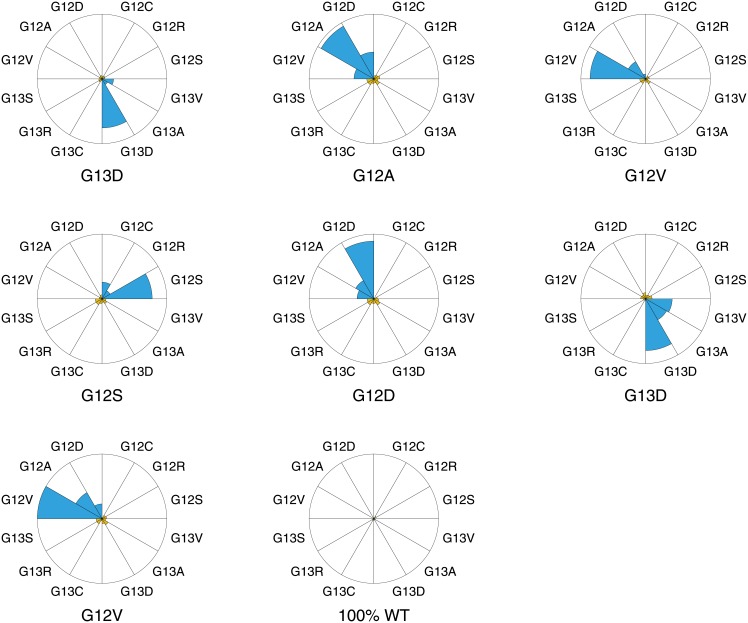
Pie diagrams of the statistical results of experiment set 3: Blinded clinical samples. The size of each pie is proportional to the vertical distance between the mutation branch and reference branch (*ρ*). The colour of each pie diagram represents the statistical significance: p-values below 0.01 are shown in blue while p-values above 0.01 are shown in orange. All values can be found in S3 Table for reference.

We analyzed seven blind coded clinical FFPE tumor samples for which sanger sequencing data of the KRAS status was available. The samples were treated as described in Materials and Methods and statistically analyzed leading to the results in S3 Table and Fig 7. For each of the seven samples the *p*′-values indicated one clear mutation. After decoding of the samples, the results of the last column of the table appeared in full agreement with sequencing data.

### Summary of statistical tests

**Fig 8:**
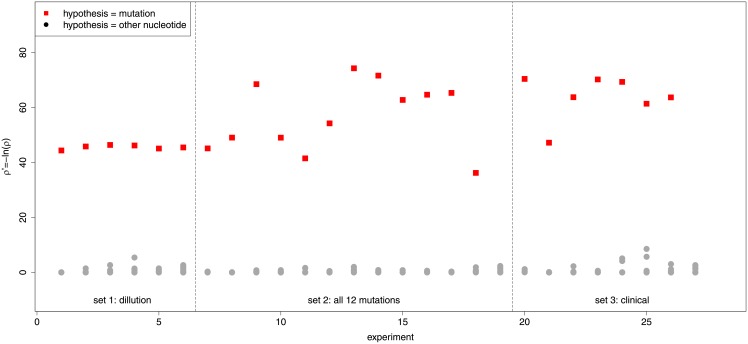
A summary of the statistical results of all three experiment sets. The *p*′-value of the most significant hypothesis (the true mutation) is indicated by a square. The *p*′-values of all the hypotheses at the other three nucleotides are indicated with a dot. Raw data can be found in S1, S2 and S3 Tables.

As a summary of the statistical tests we first note that in each sample the correct mutant was retrieved as the most statistically significant hypothesis. As a further analysis we combine all experimental results and present them in Fig 8. Here, for each sample, the *p*′-value of the most significant hypothesis (the true mutation) is indicated and compared with the *p*′-values of the other hypotheses (the other three nucleotides). This figure shows a clear gap between the correct and false hypotheses indicating that it is not difficult to set a significance threshold to distinguish true from false positives. As an extra test, the *p*′-values of the WT experiments (nr. 19 and 27) are also well below those of the mutant hypotheses in mutant-containing samples.

## Discussion

In this paper we developed a framework to assess the capability of hybridization to detect point mutations present in a small percentage in mixed samples. We use a solid support nucleic acid platform and consider 12 clinically highly relevant hotspot point mutations in exon 2 of the *KRAS* oncogene. Clinical samples for which this test could be used always contain a mixture of cancer and non-cancer cells, the former is in minority and is a potential carrier of a point mutation. Using a dilution series with a decreasing amount of mutant DNA we first show a quantitative agreement between measured values and a suited thermodynamics theory. Using this agreement we next determine the detection limit of mutant DNA without the need to perform dilution series for each mutation. The results show that each mutation can be detected at levels at least as low as 1% of mutant, which makes it clinically highly relevant.

The current manuscript builds on previous work on DNA hybridization [6–8, 12, 15, 16], the novelty of the presented work is in the use of DNA thermodynamics in the design of an assay capable to detect multiple and specific mutations with a well-defined limit of detection derived from physical principles and capable to perform this on clinical FFPE samples. The example of the FFPE samples illustrates that our method can deal with the limited quality and heterogeneity of the tissue material. Due to the intrinsic parallel character of the microarray technology, this approach makes it possible to screen for hundreds of different point mutations in a single run.

This work shows that enzyme-free detection of point mutations via DNA hybridization is a highly quantitative technique with an accuracy, robustness and parallelization suited for clinical applications. The results also support and complement the research efforts in hybridization-based methods [17–27]. By using the concepts of DNA thermodynamics upon hybridization, the current technique has the advantage that mutations with low relative abundance can be detected in a higher parallelization level than PCR-based methods. In comparison to sequencing techniques, we can state that the process of hybridization is a very elementary reaction in which no enzymes are involved, which opens doors for further low-tech detection assays at physiological conditions [28]. Last but not least, the framework presented in this paper is platform-free and shows the advantage of a good description of the underlying physico-chemical principles.

## Supporting information

S1 Fig Scatter plots for experiment set 2 and 3. (PDF)

S1 Table Statistical results of experiment set 1: Concentration range experiments with gBlocks samples (mutation 10C→G). (PDF)

S2 Table Statistical results of experiment set 2: 12 gBlocks samples with each mutant present at 5%. (PDF)

S3 Table Statistical results of experiment set 3: Blinded clinical samples. (PDF)
