# Supplementary material for: Thermodynamic framework to assess low abundance DNA mutation detection by hybridization
Source: PLoS One. 2017 May 25;12(5):e0177384. doi: 10.1371/journal.pone.0177384 (PMC5444680; doi:10.1371/journal.pone.0177384)
Supplement: S1 Fig — (PDF) [file pone.0177384.s001.pdf]

# Supplementary Figures: Thermodynamic Framework to Assess Low Abundance DNA Mutation Detection by Hybridization

H. Willems[1], A. Jacobs[1], W. W. Hadiwikarta[1,2], T. Venken[1,3], D. Valkenborg[1,4], N. Van Roy[5], J. Vandesompele[5,6], J. Hooyberghs[1,7,*]

**1 Flemish Institute for Technological Research, VITO, Mol, Belgium**
**2 Institute for Theoretical Physics, KULeuven, Leuven, Belgium**
**3 Laboratory for Biomolecular Modelling, KULeuven, Leuven, Belgium**
**4 Interuniversity Institute for Biostatistics and Statistical Bioinformatics, Hasselt University, Diepenbeek, Belgium.**
**5 Center for Medical Genetics Ghent (CMGG), Ghent University, Ghent, Belgium**
**6 Cancer Research Institute Ghent (CRIG), Ghent University, Ghent, Belgium**
**7 Theoretical Physics, Hasselt University, Diepenbeek, Belgium**

*** Corresponding author: jef.hooyberghs@vito.be**

Raw agilent data files can be found online at `https://figshare.com/articles/Experiments_zip/4785643`

Figures for the concentration rang experiments (experiment set 1) are shown in the main text of the article. The other results can be found below and are visualized using the three different probe lengths (L=21, 23 and 25).

# Experiment set 2: 12 gBlocks samples with each mutant present at 5%.

## Experiment 7, G13C

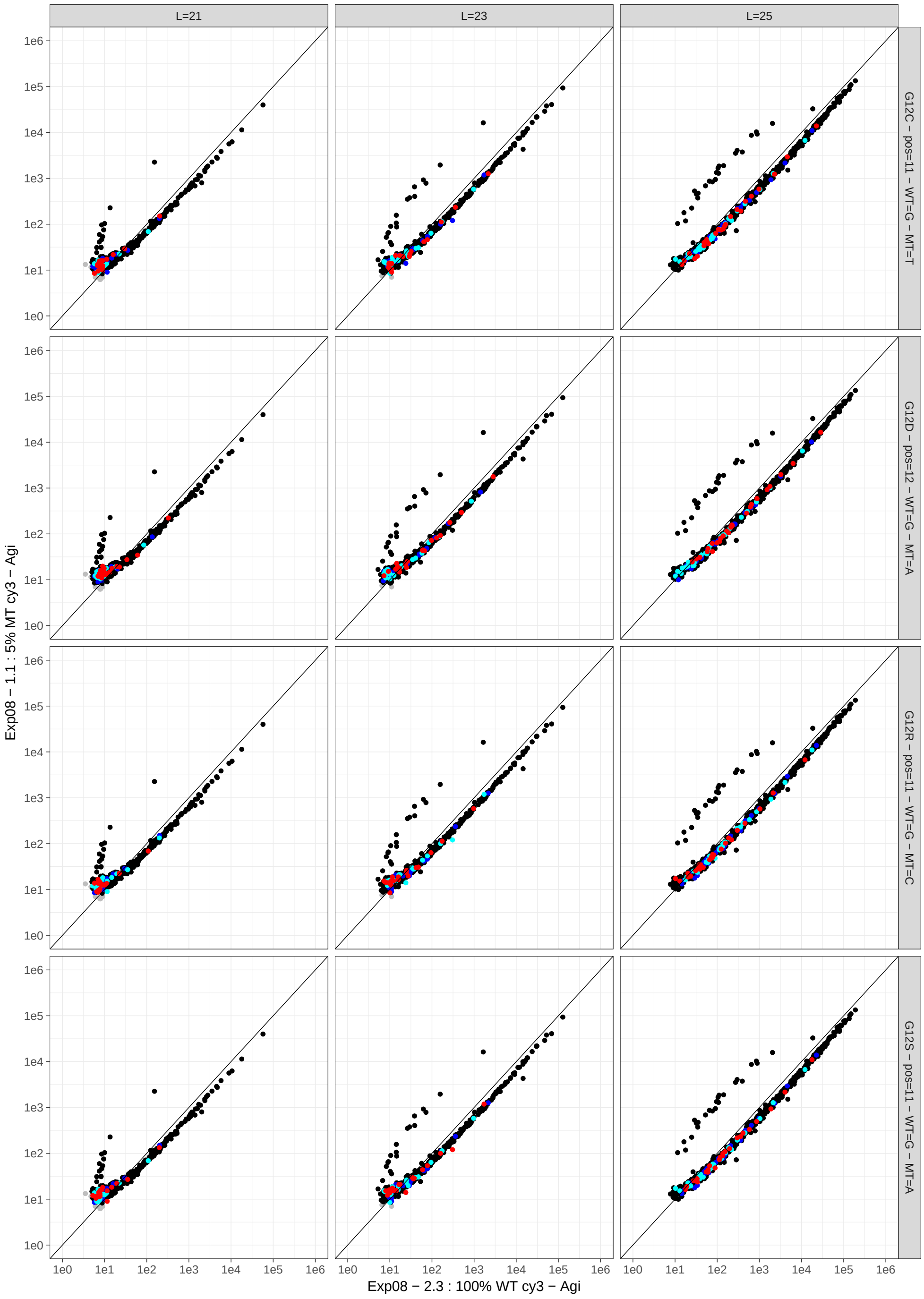

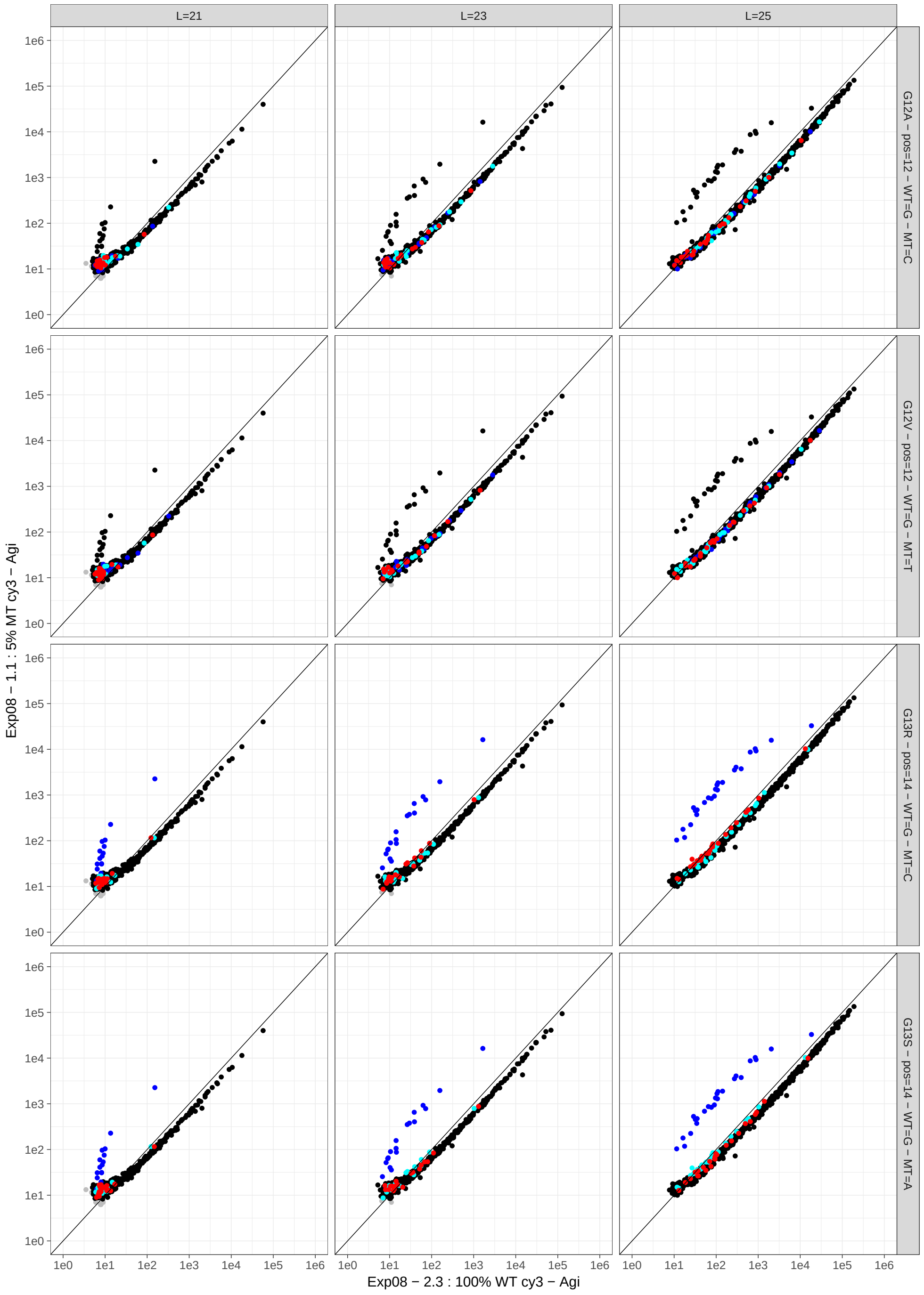

L=21
L=23
L=25
G12A − pos=12 − WT=G − MT=C
G12V − pos=12 − WT=G − MT=T
G13R − pos=14 − WT=G − MT=C
G13S − pos=14 − WT=G − MT=A
1e0
1e1
1e2
1e3
1e4
1e5
1e6
Exp08 − 1.1 : 5% MT cy3 − Agi
Exp08 − 2.3 : 100% WT cy3 − Agi

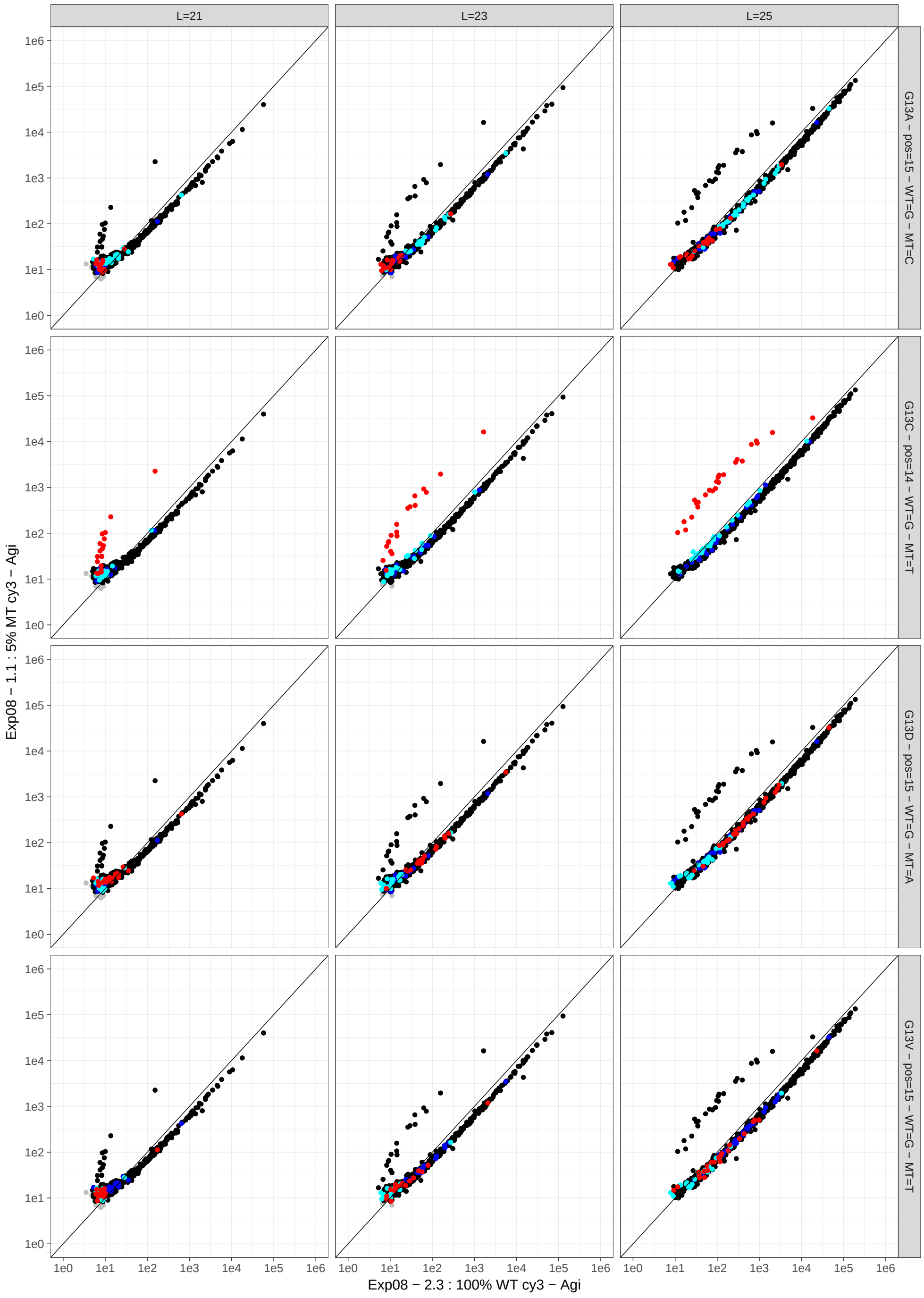
L=21
L=23
L=25
G13A − pos=15 − WT=G − MT=C
G13C − pos=14 − WT=G − MT=T
G13D − pos=15 − WT=G − MT=A
G13V − pos=15 − WT=G − MT=T
1e0
1e1
1e2
1e3
1e4
1e5
1e6
Exp08 − 1.1 : 5% MT cy3 − Agi
Exp08 − 2.3 : 100% WT cy3 − Agi

# Experiment 8, G12R

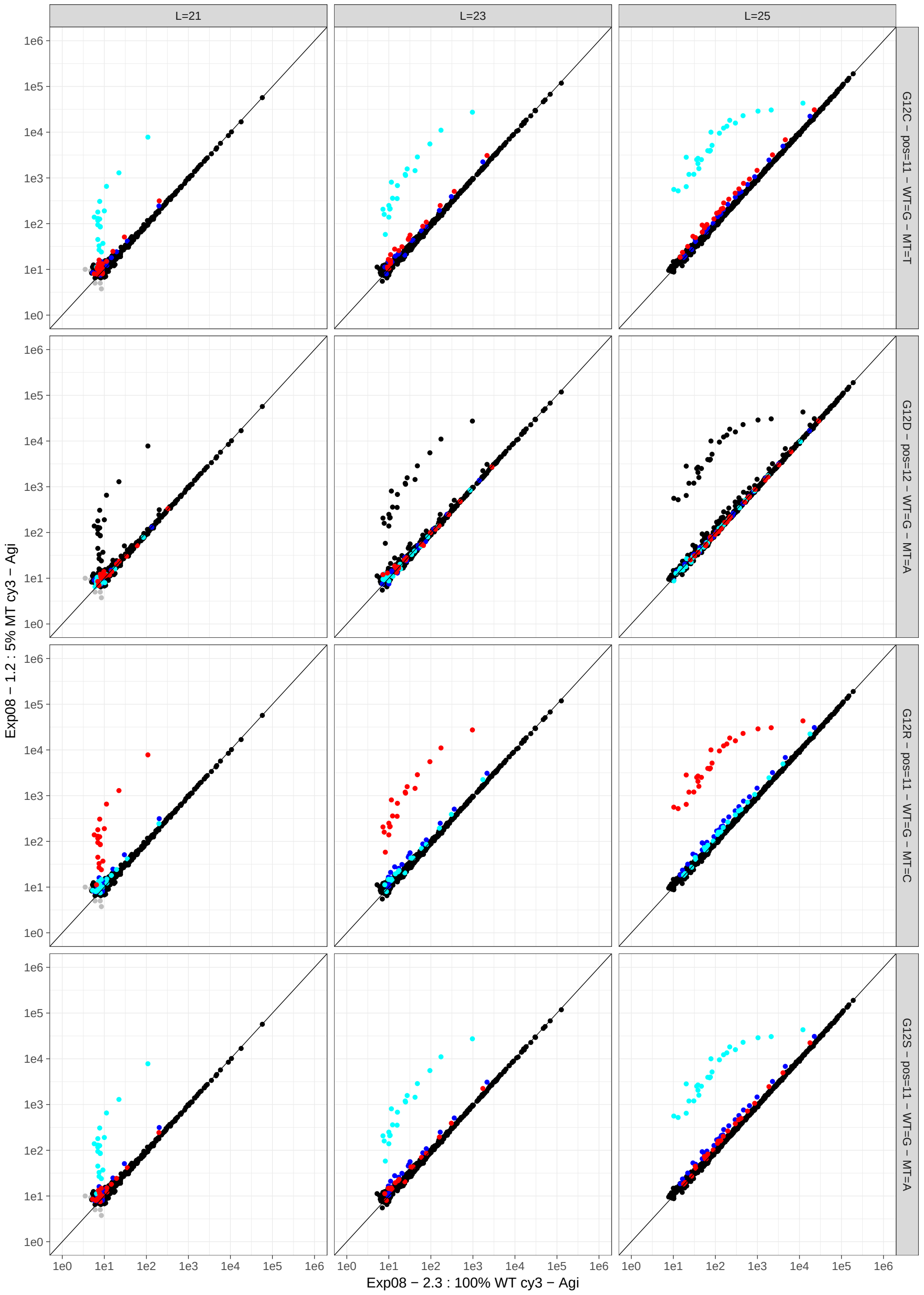

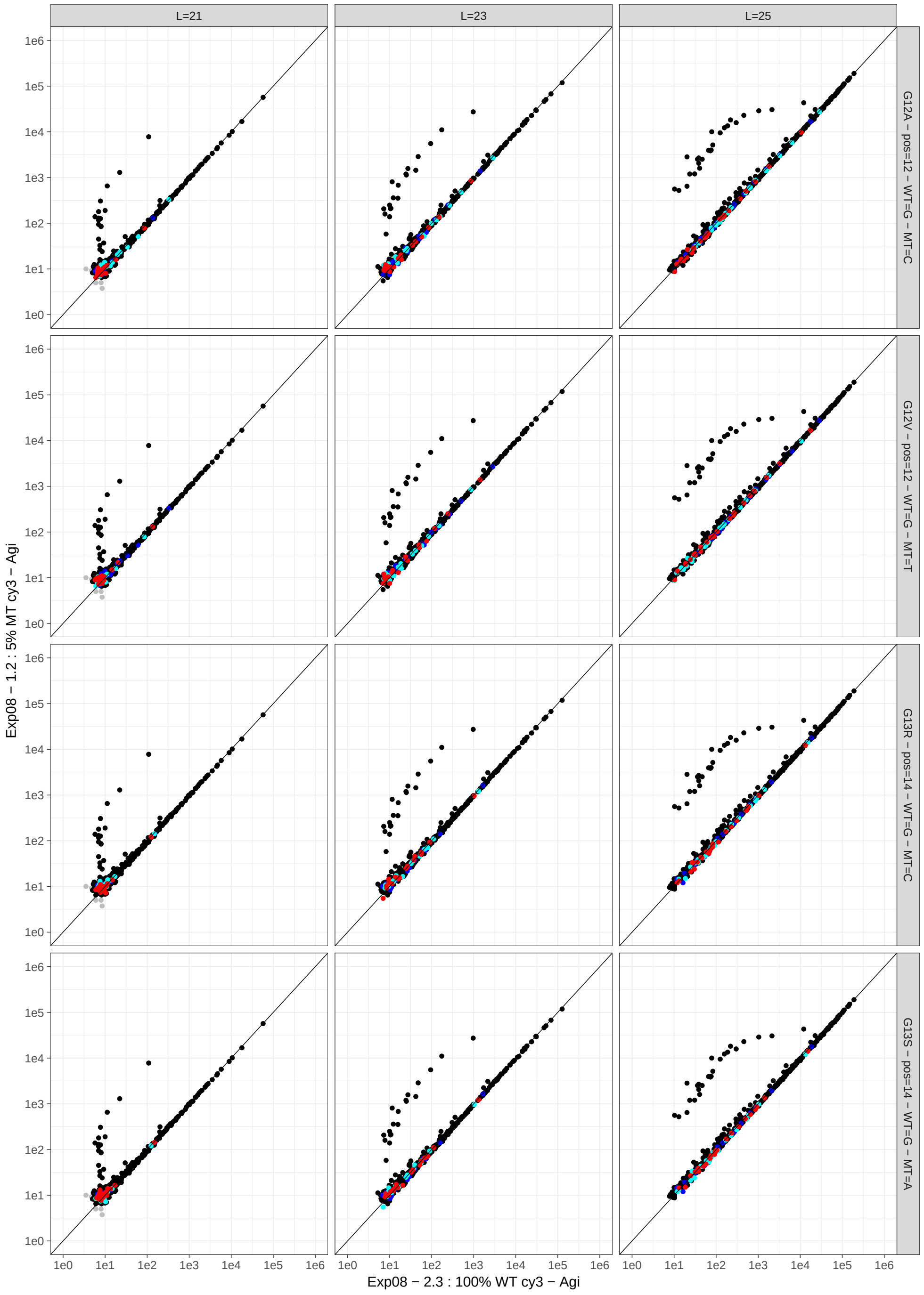
L=21
L=23
L=25
G12A − pos=12 − WT=G − MT=C
G12V − pos=12 − WT=G − MT=T
G13R − pos=14 − WT=G − MT=C
G13S − pos=14 − WT=G − MT=A
Exp08 − 1.2 : 5% MT cy3 − Agi
Exp08 − 2.3 : 100% WT cy3 − Agi
1e0
1e1
1e2
1e3
1e4
1e5
1e6

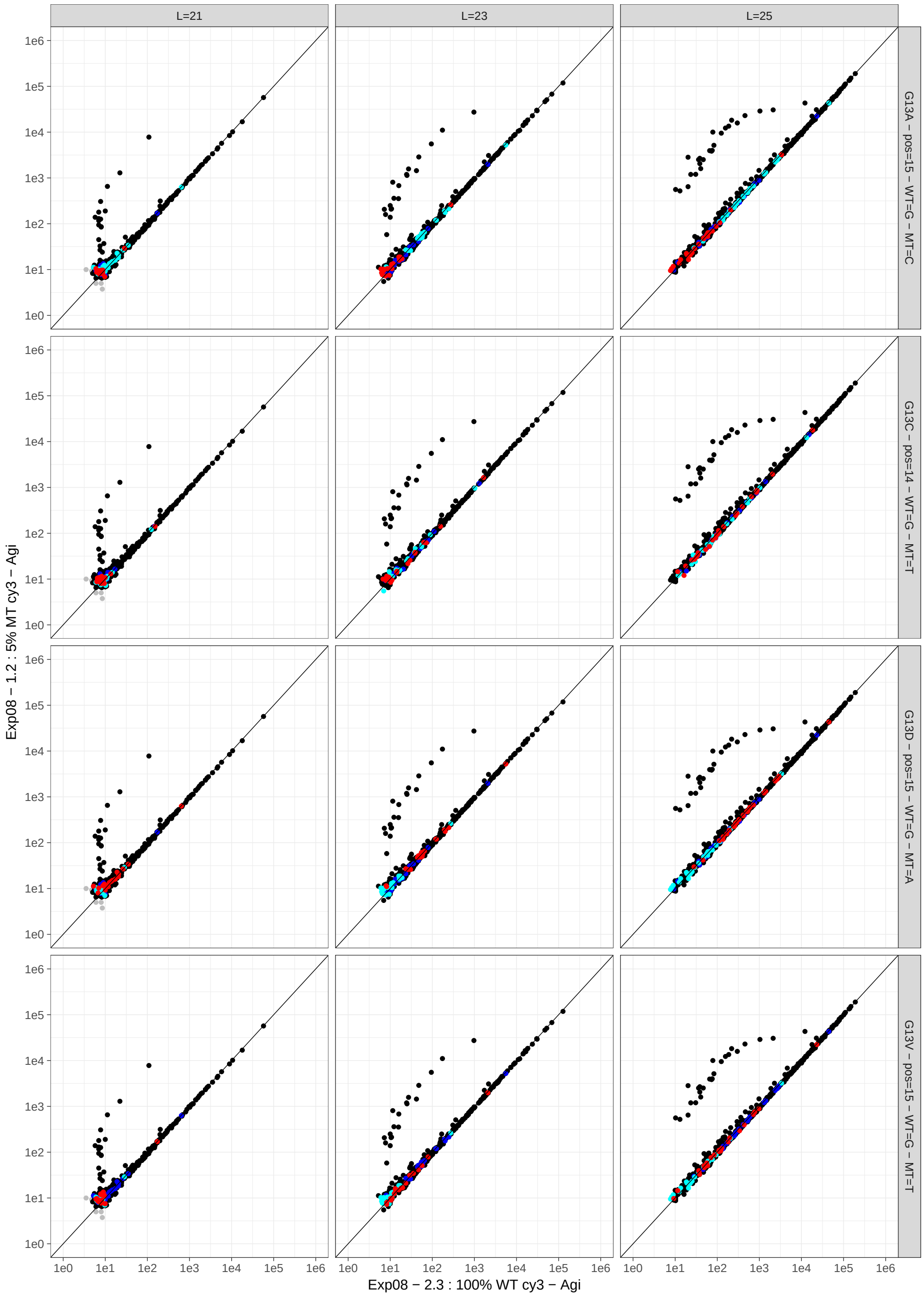
L=21
L=23
L=25
G13A − pos=15 − WT=G − MT=C
G13C − pos=14 − WT=G − MT=T
G13D − pos=15 − WT=G − MT=A
G13V − pos=15 − WT=G − MT=T
Exp08 − 1.2 : 5% MT cy3 − Agi
Exp08 − 2.3 : 100% WT cy3 − Agi
1e0
1e1
1e2
1e3
1e4
1e5
1e6

## Experiment 9, G12D

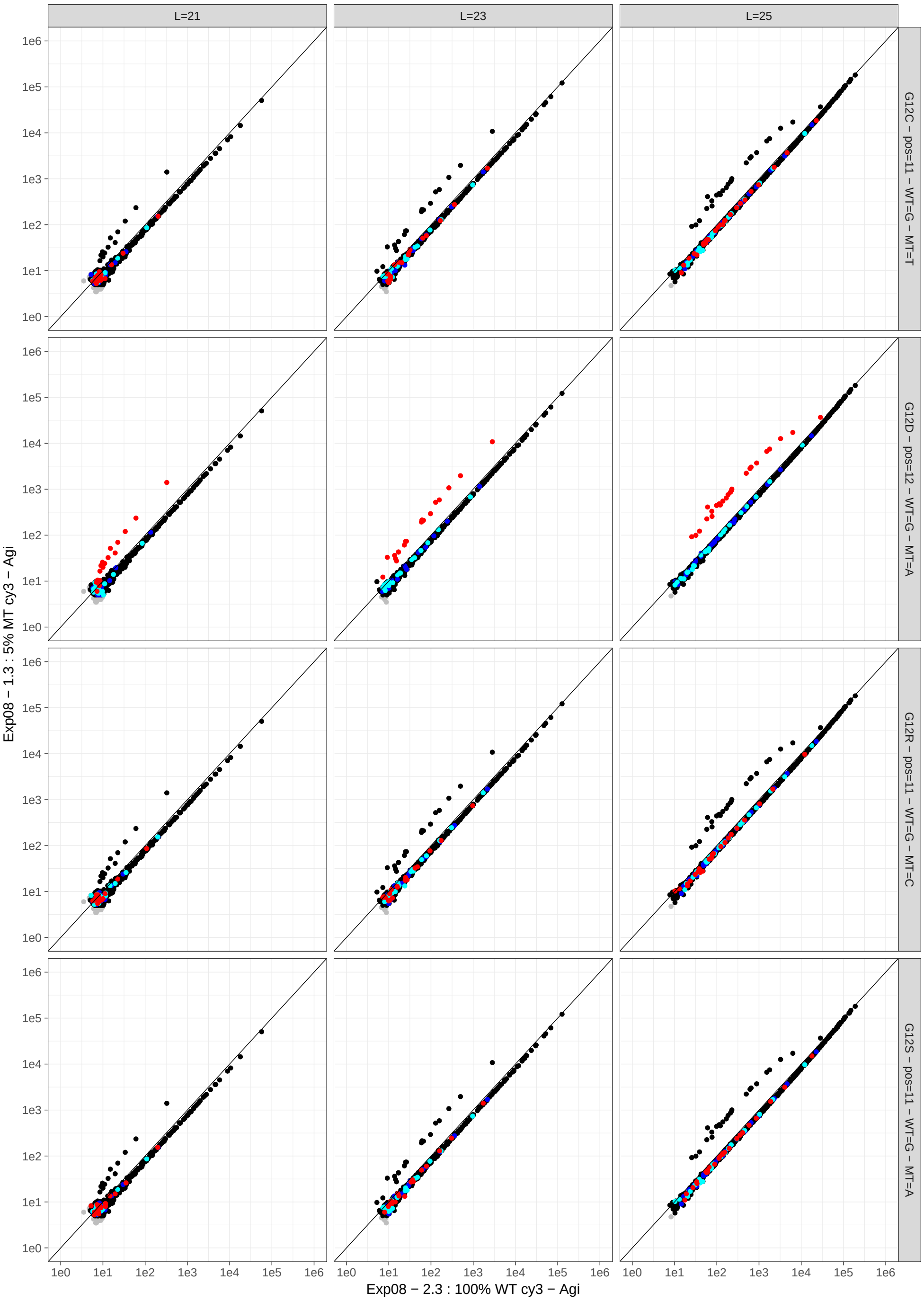

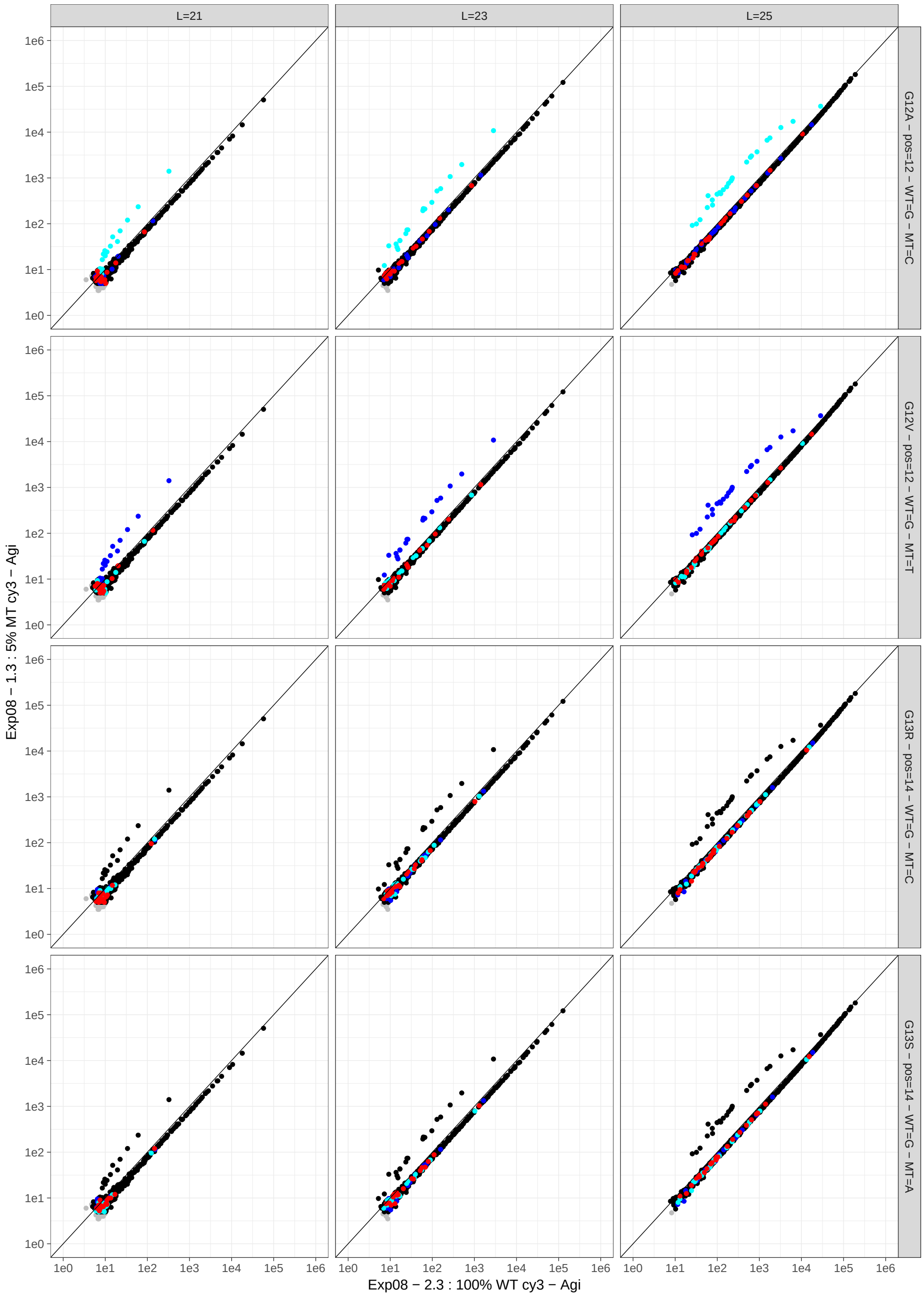
L=21
L=23
L=25
G12A − pos=12 − WT=G − MT=C
G12V − pos=12 − WT=G − MT=T
G13R − pos=14 − WT=G − MT=C
G13S − pos=14 − WT=G − MT=A
1e0
1e1
1e2
1e3
1e4
1e5
1e6
Exp08 − 1.3 : 5% MT cy3 − Agi
Exp08 − 2.3 : 100% WT cy3 − Agi

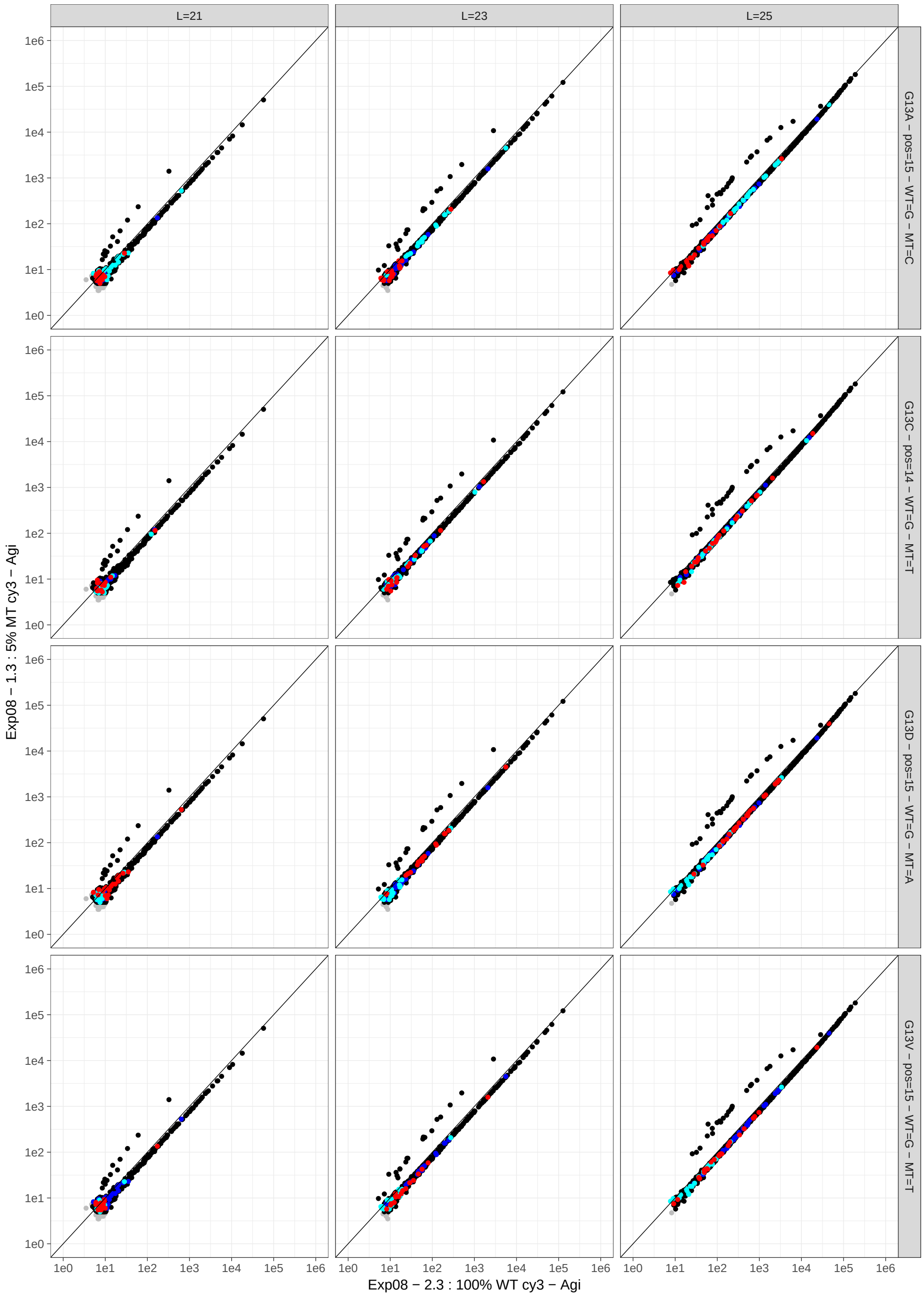
L=21
L=23
L=25
G13A − pos=15 − WT=G − MT=C
G13C − pos=14 − WT=G − MT=T
G13D − pos=15 − WT=G − MT=A
G13V − pos=15 − WT=G − MT=T
1e0
1e1
1e2
1e3
1e4
1e5
1e6
Exp08 − 1.3 : 5% MT cy3 − Agi
Exp08 − 2.3 : 100% WT cy3 − Agi

## Experiment 10, G12C

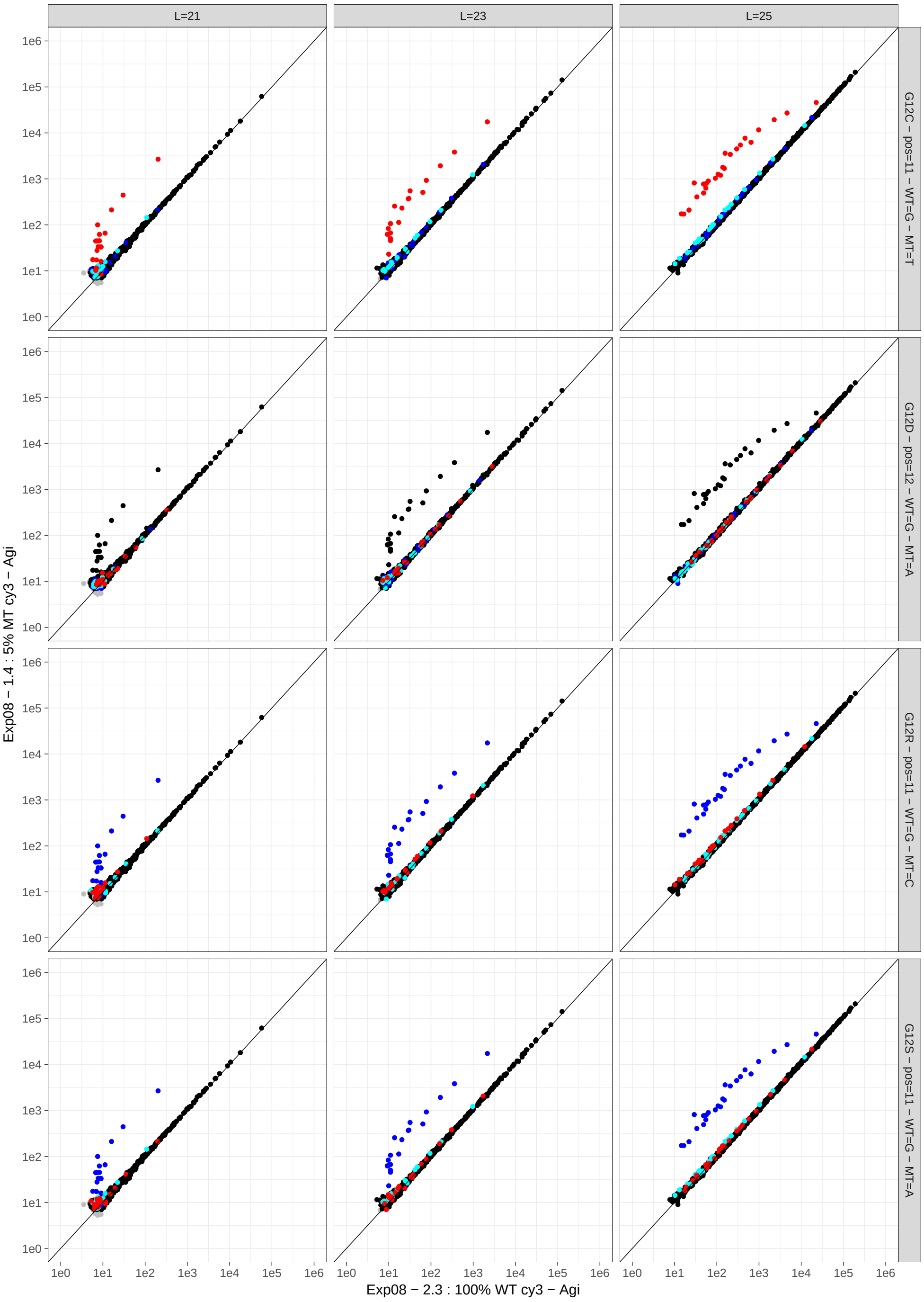

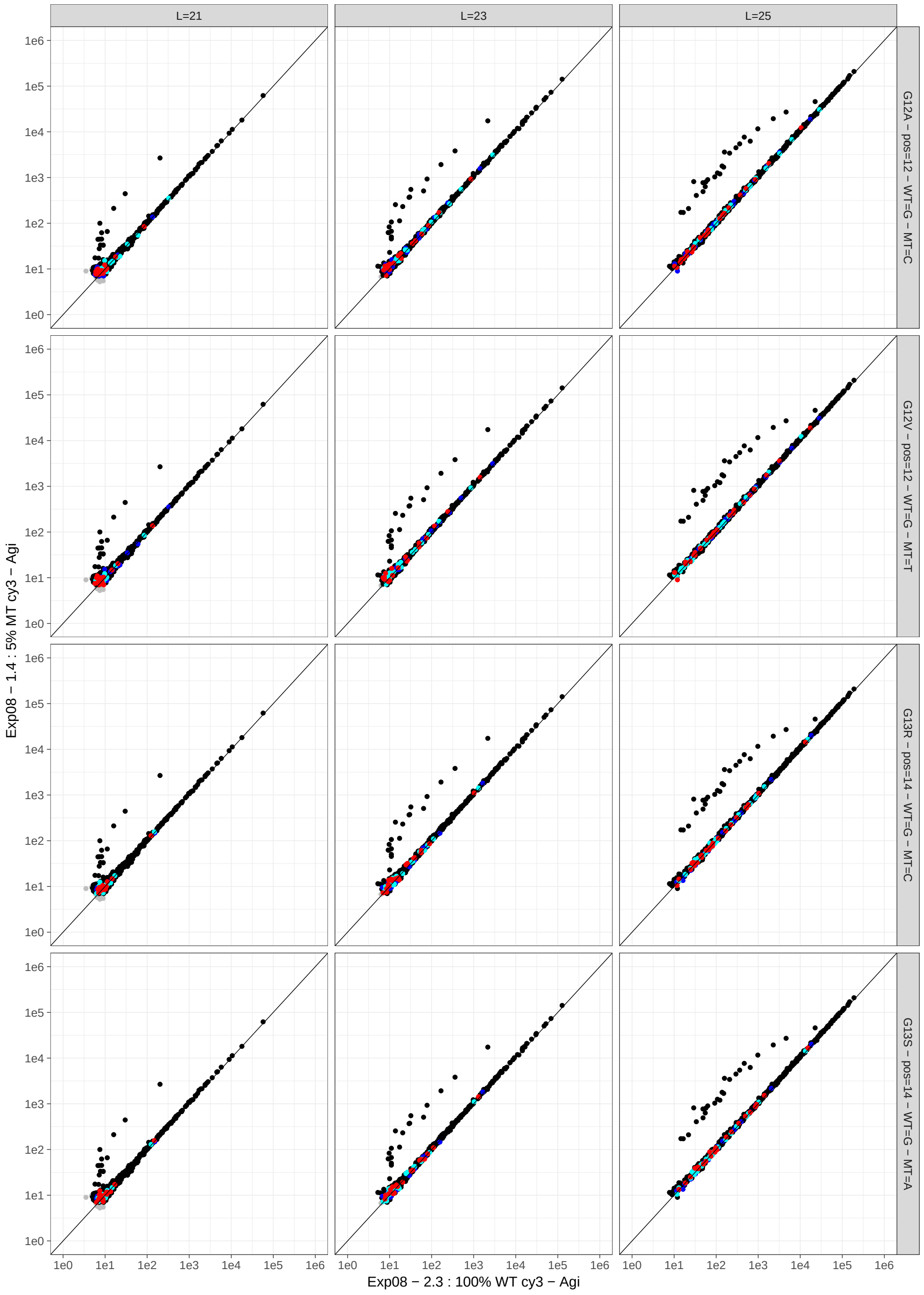
L=21
L=23
L=25
G12A − pos=12 − WT=G − MT=C
G12V − pos=12 − WT=G − MT=T
G13R − pos=14 − WT=G − MT=C
G13S − pos=14 − WT=G − MT=A
1e0
1e1
1e2
1e3
1e4
1e5
1e6
Exp08 − 1.4 : 5% MT cy3 − Agi
Exp08 − 2.3 : 100% WT cy3 − Agi

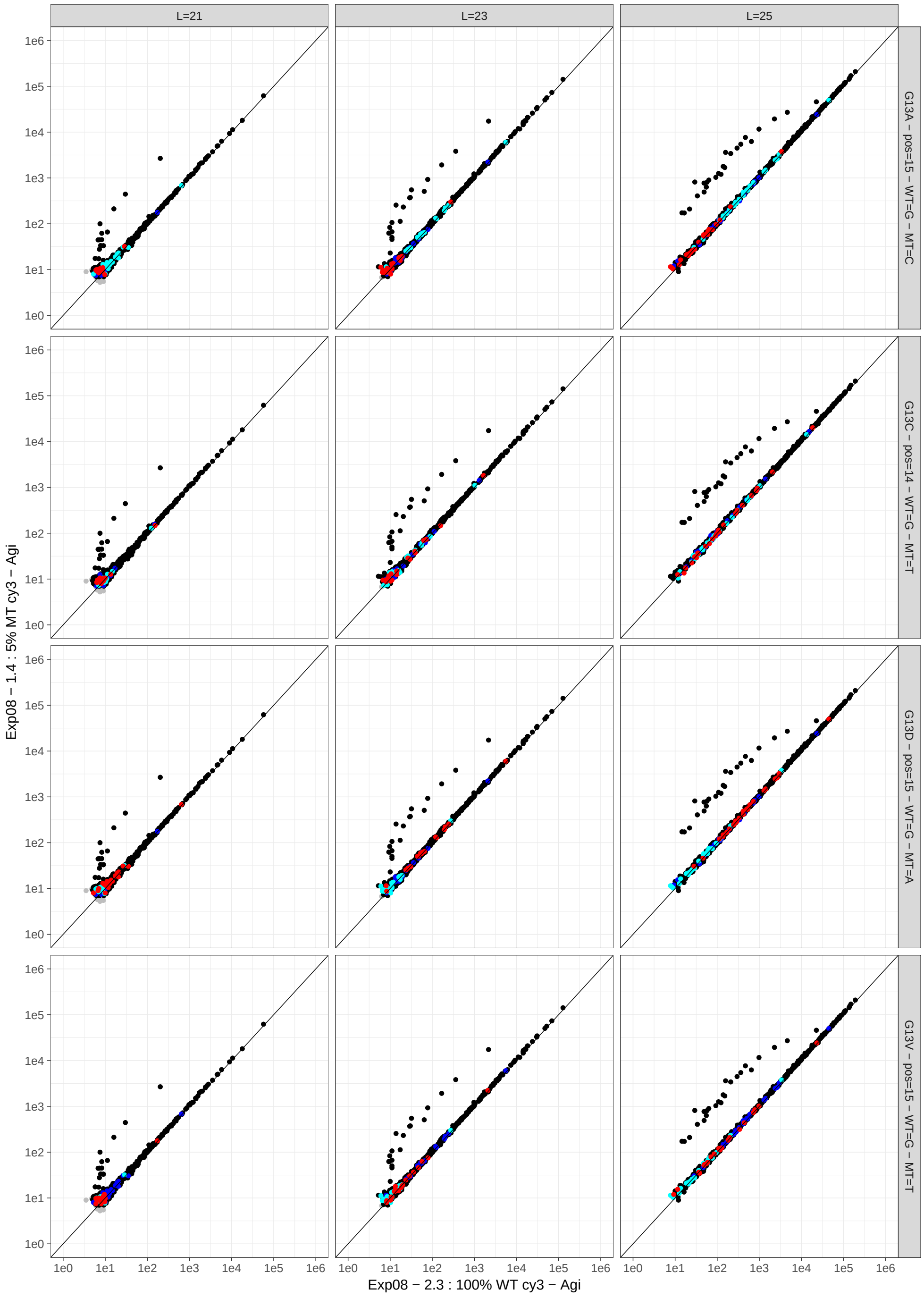

L=21
L=23
L=25
G13A − pos=15 − WT=G − MT=C
G13C − pos=14 − WT=G − MT=T
G13D − pos=15 − WT=G − MT=A
G13V − pos=15 − WT=G − MT=T
Exp08 − 1.4 : 5% MT cy3 − Agi
Exp08 − 2.3 : 100% WT cy3 − Agi
1e0
1e1
1e2
1e3
1e4
1e5
1e6

## Experiment 11, G12A

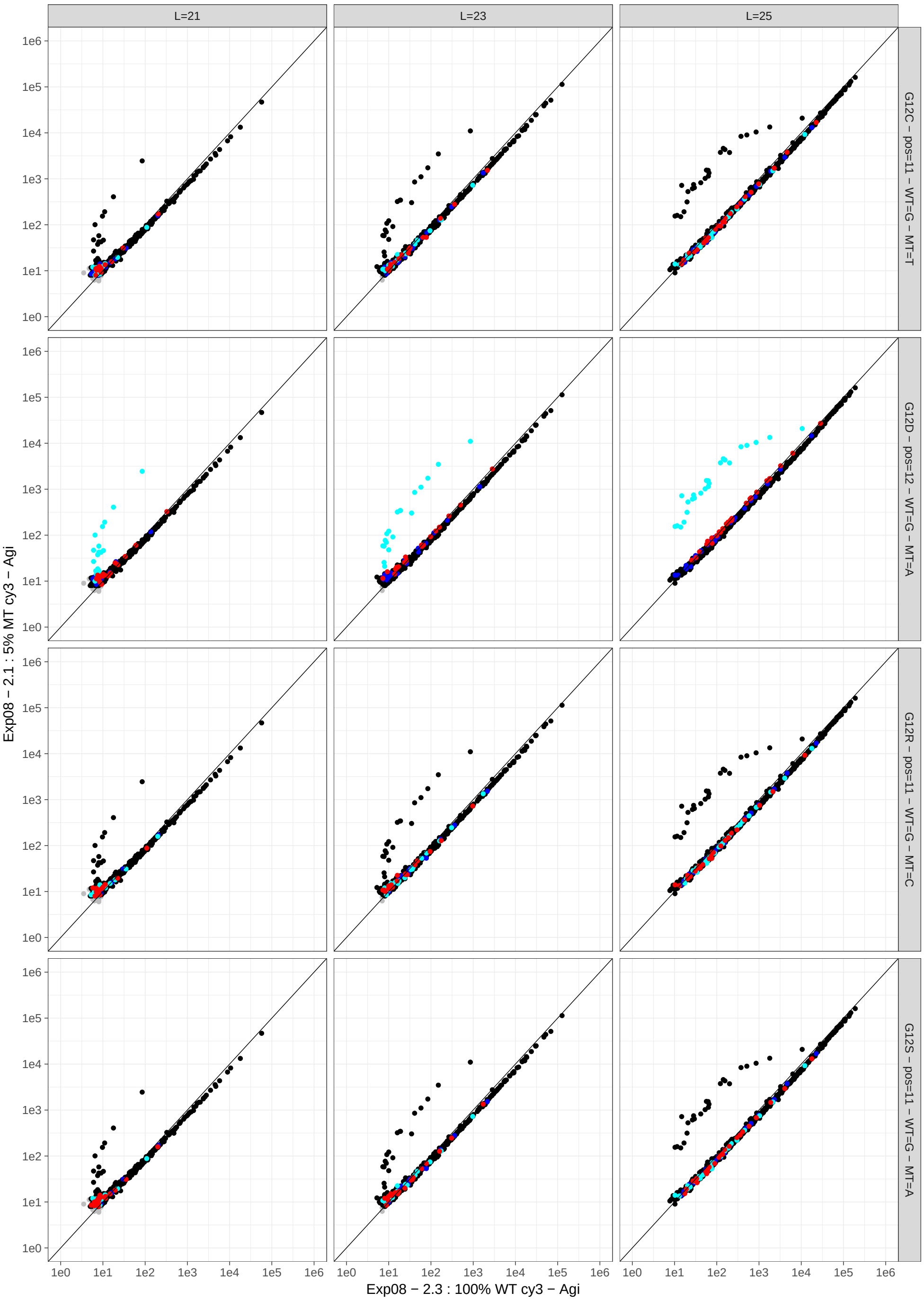

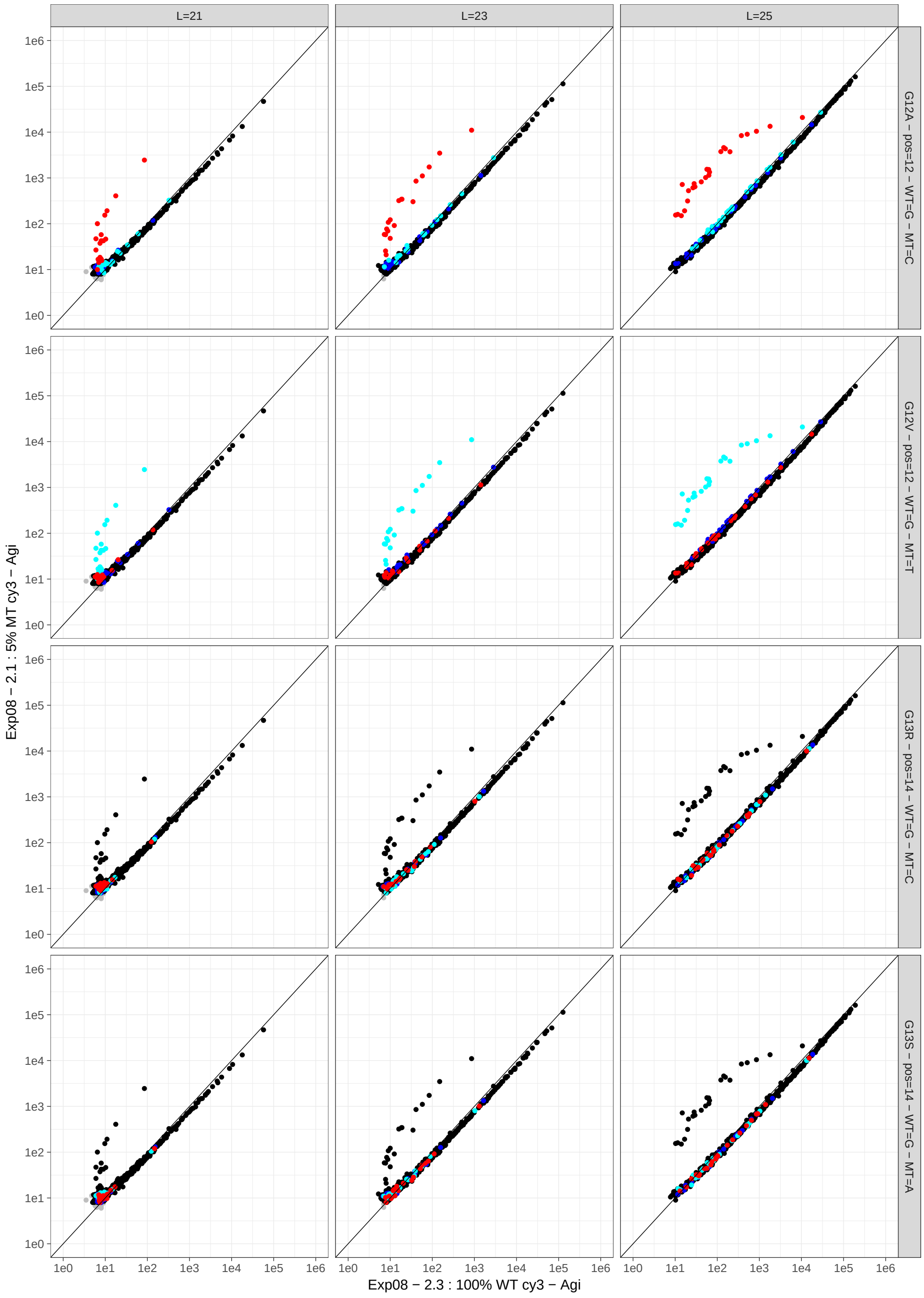
L=21
L=23
L=25
G12A – pos=12 – WT=G – MT=C
G12V – pos=12 – WT=G – MT=T
G13R – pos=14 – WT=G – MT=C
G13S – pos=14 – WT=G – MT=A
Exp08 – 2.1 : 5% MT cy3 – Agi
Exp08 – 2.3 : 100% WT cy3 – Agi
1e0
1e1
1e2
1e3
1e4
1e5
1e6

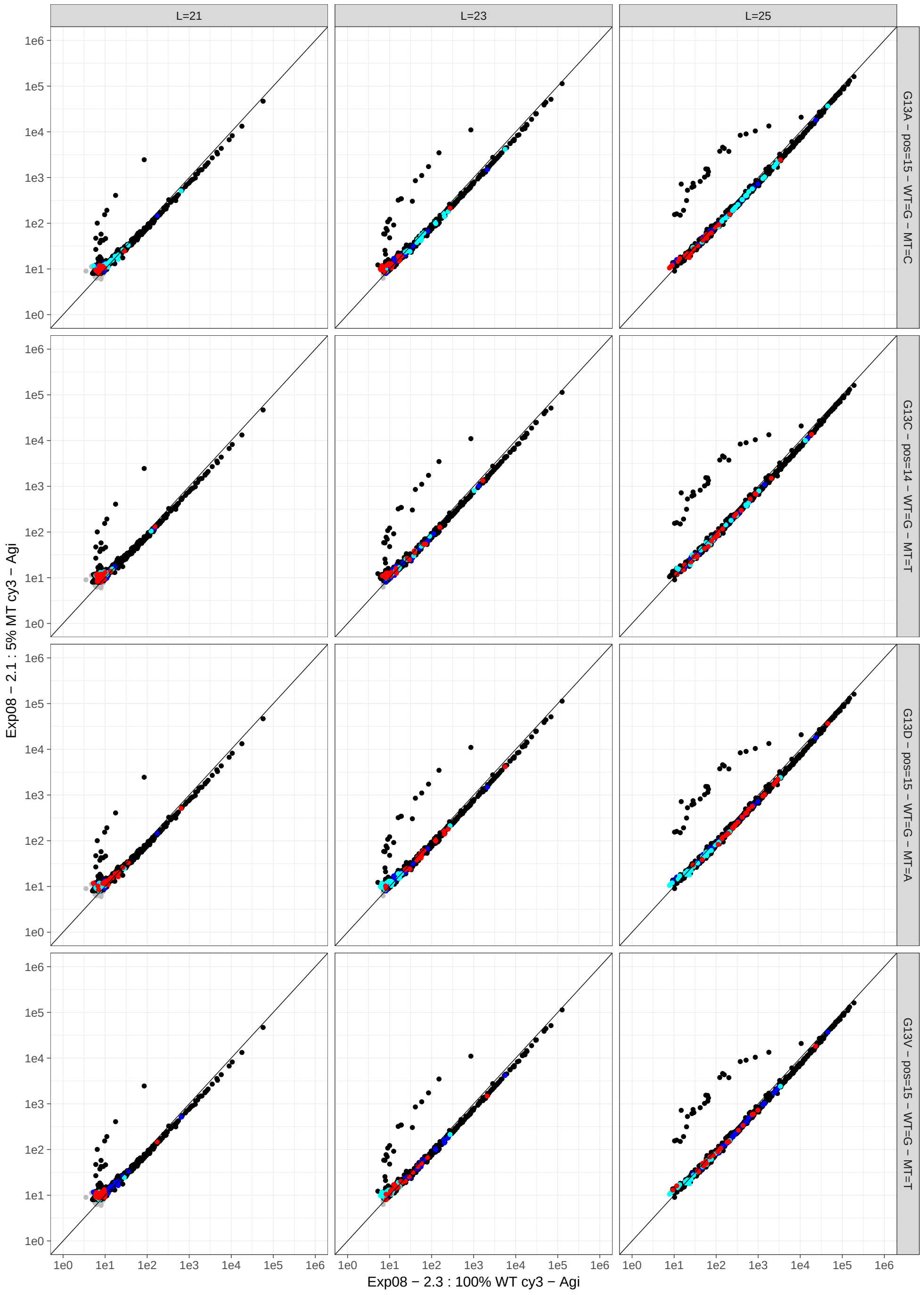

L=21
L=23
L=25
G13A − pos=15 − WT=G − MT=C
G13C − pos=14 − WT=G − MT=T
G13D − pos=15 − WT=G − MT=A
G13V − pos=15 − WT=G − MT=T
Exp08 − 2.1 : 5% MT cy3 − Agi
Exp08 − 2.3 : 100% WT cy3 − Agi
1e0
1e1
1e2
1e3
1e4
1e5
1e6

# Experiment 12, G13S

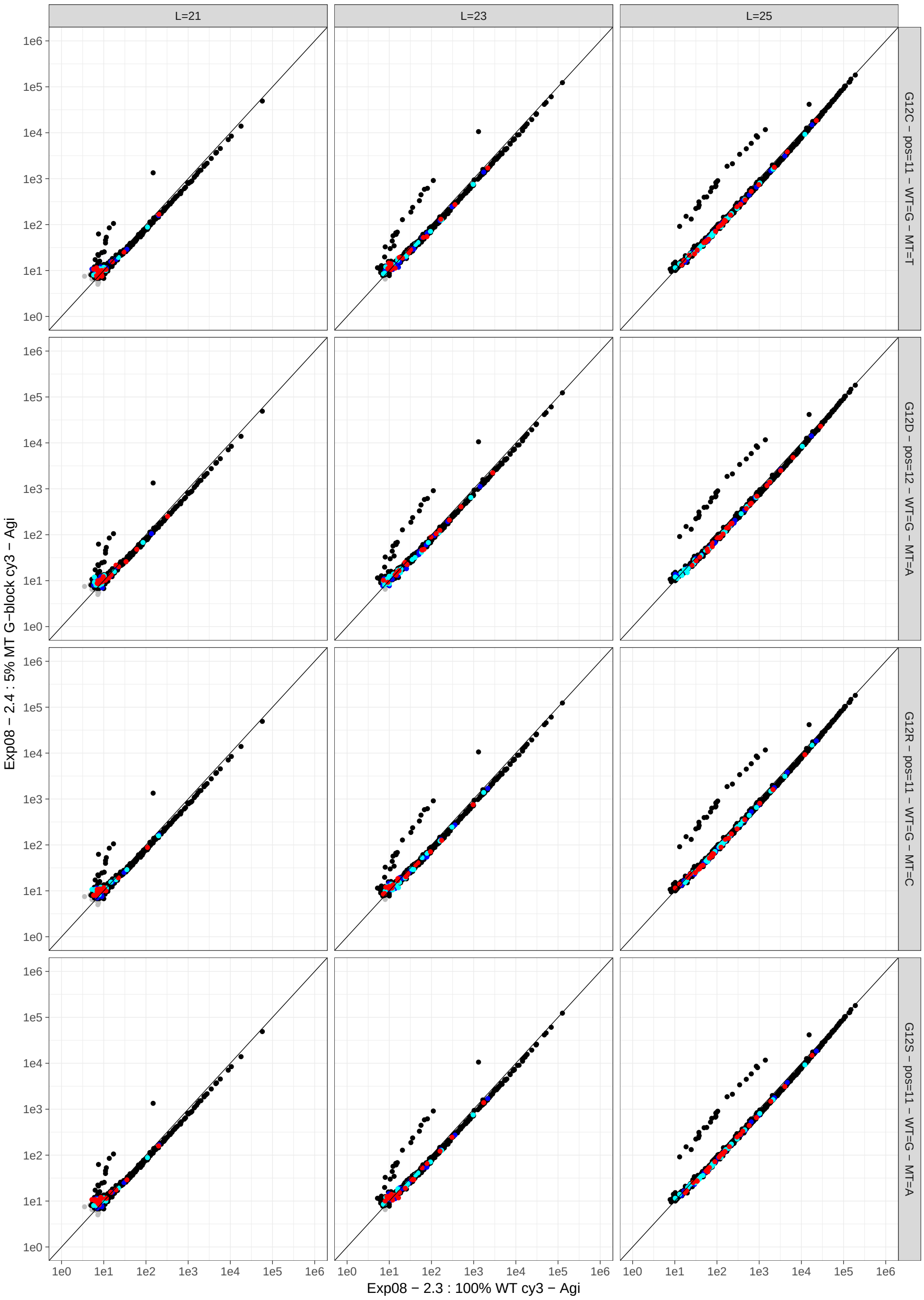

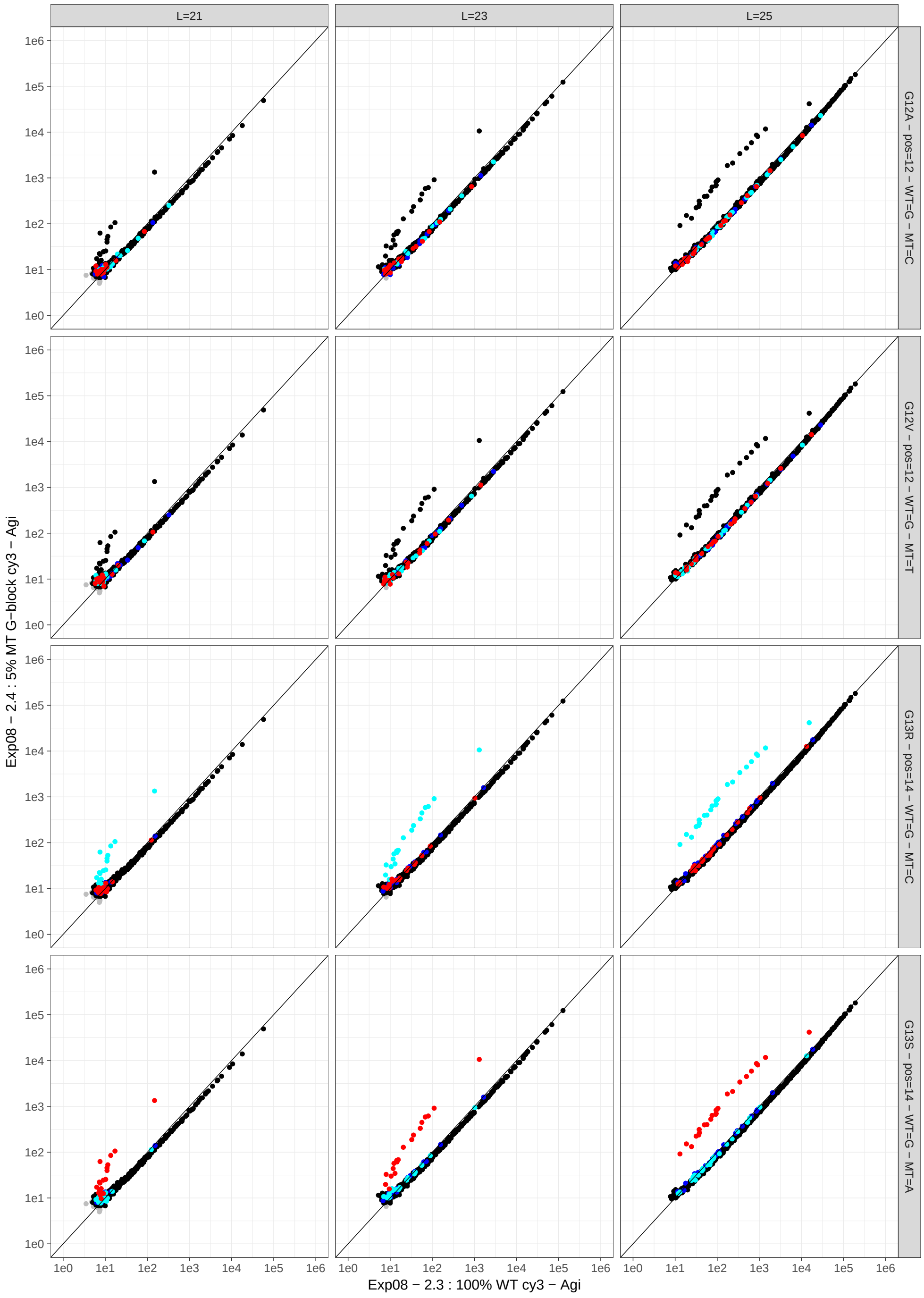
L=21
L=23
L=25
G12A – pos=12 – WT=G – MT=C
G12V – pos=12 – WT=G – MT=T
G13R – pos=14 – WT=G – MT=C
G13S – pos=14 – WT=G – MT=A
Exp08 − 2.4 : 5% MT G−block cy3 − Agi
Exp08 − 2.3 : 100% WT cy3 − Agi
1e0
1e1
1e2
1e3
1e4
1e5
1e6

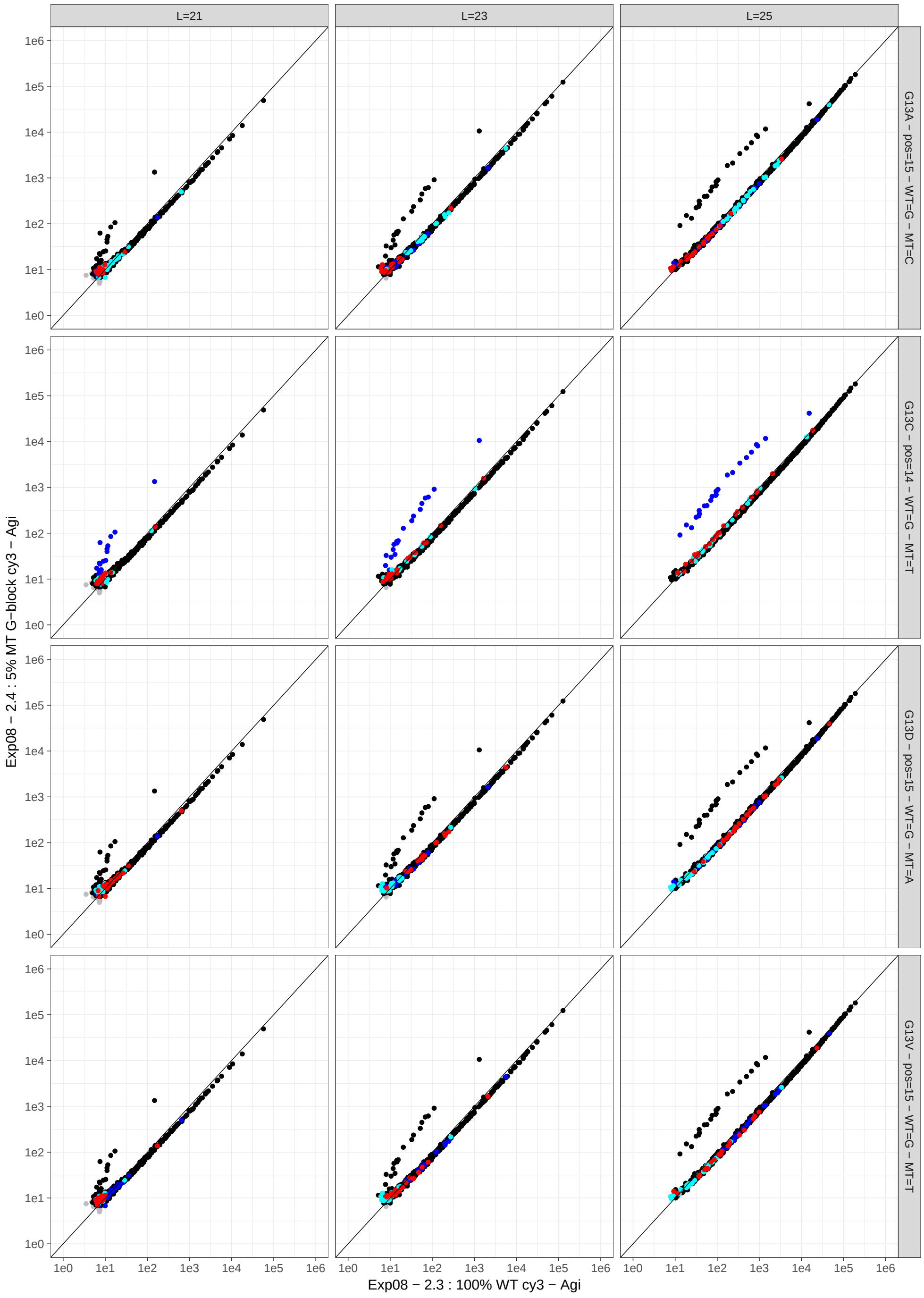

L=21
L=23
L=25
G13A − pos=15 − WT=G − MT=C
G13C − pos=14 − WT=G − MT=T
G13D − pos=15 − WT=G − MT=A
G13V − pos=15 − WT=G − MT=T
Exp08 − 2.4 : 5% MT G−block cy3 − Agi
Exp08 − 2.3 : 100% WT cy3 − Agi
1e0
1e1
1e2
1e3
1e4
1e5
1e6

## Experiment 13, G12S

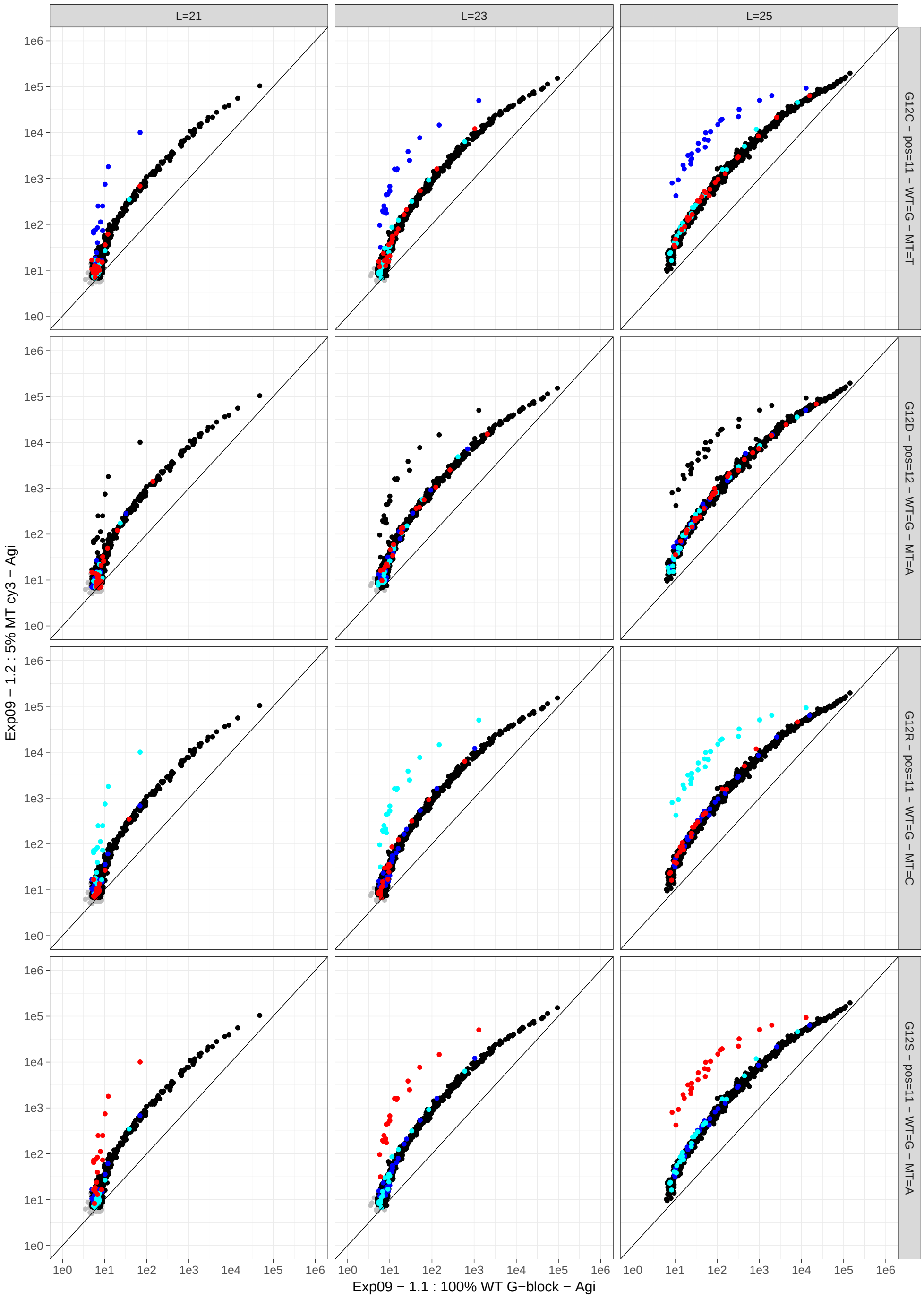

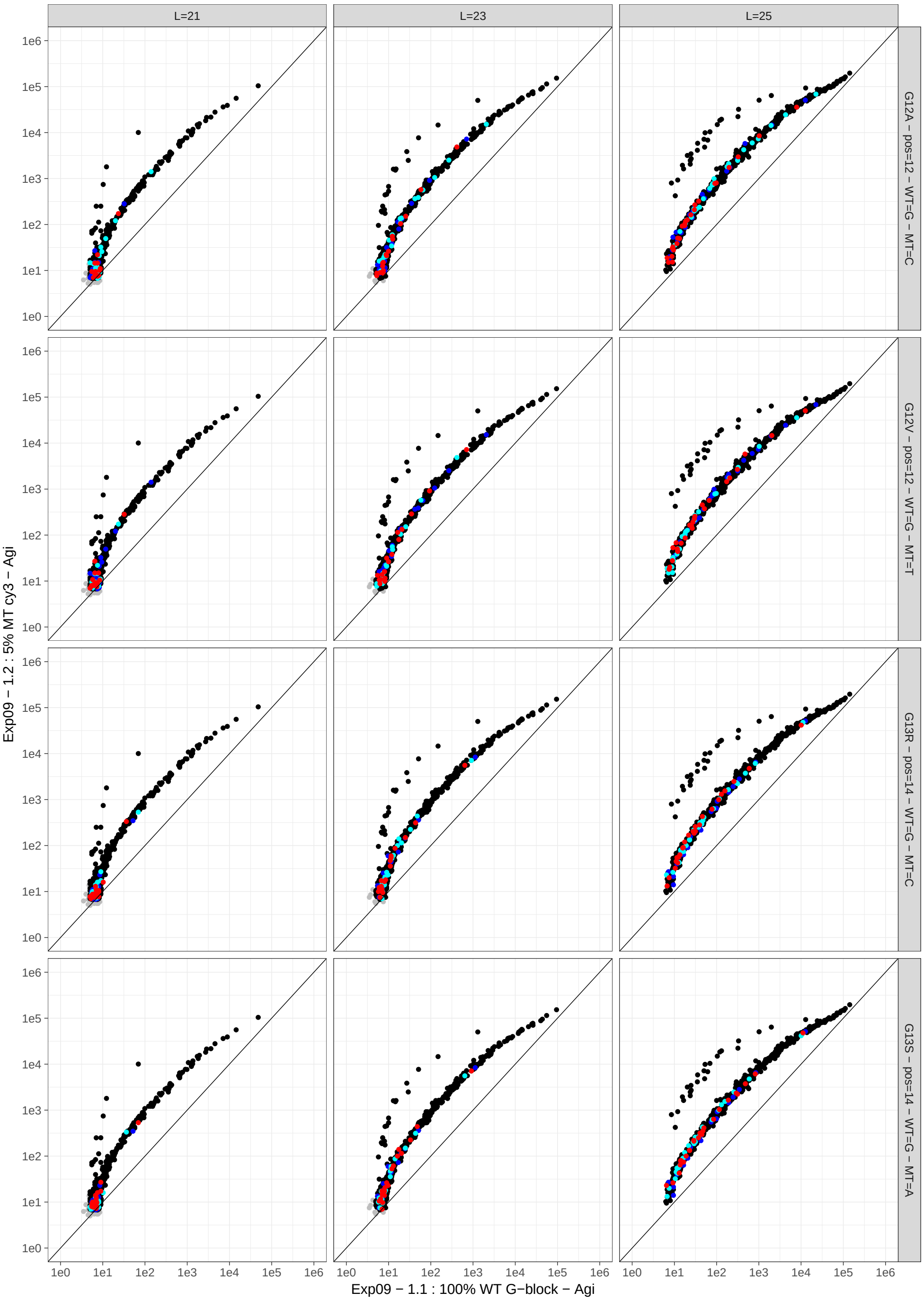

L=21
L=23
L=25
G12A − pos=12 − WT=G − MT=C
G12V − pos=12 − WT=G − MT=T
G13R − pos=14 − WT=G − MT=C
G13S − pos=14 − WT=G − MT=A
Exp09 − 1.2 : 5% MT cy3 − Agi
Exp09 − 1.1 : 100% WT G−block − Agi
1e0
1e1
1e2
1e3
1e4
1e5
1e6

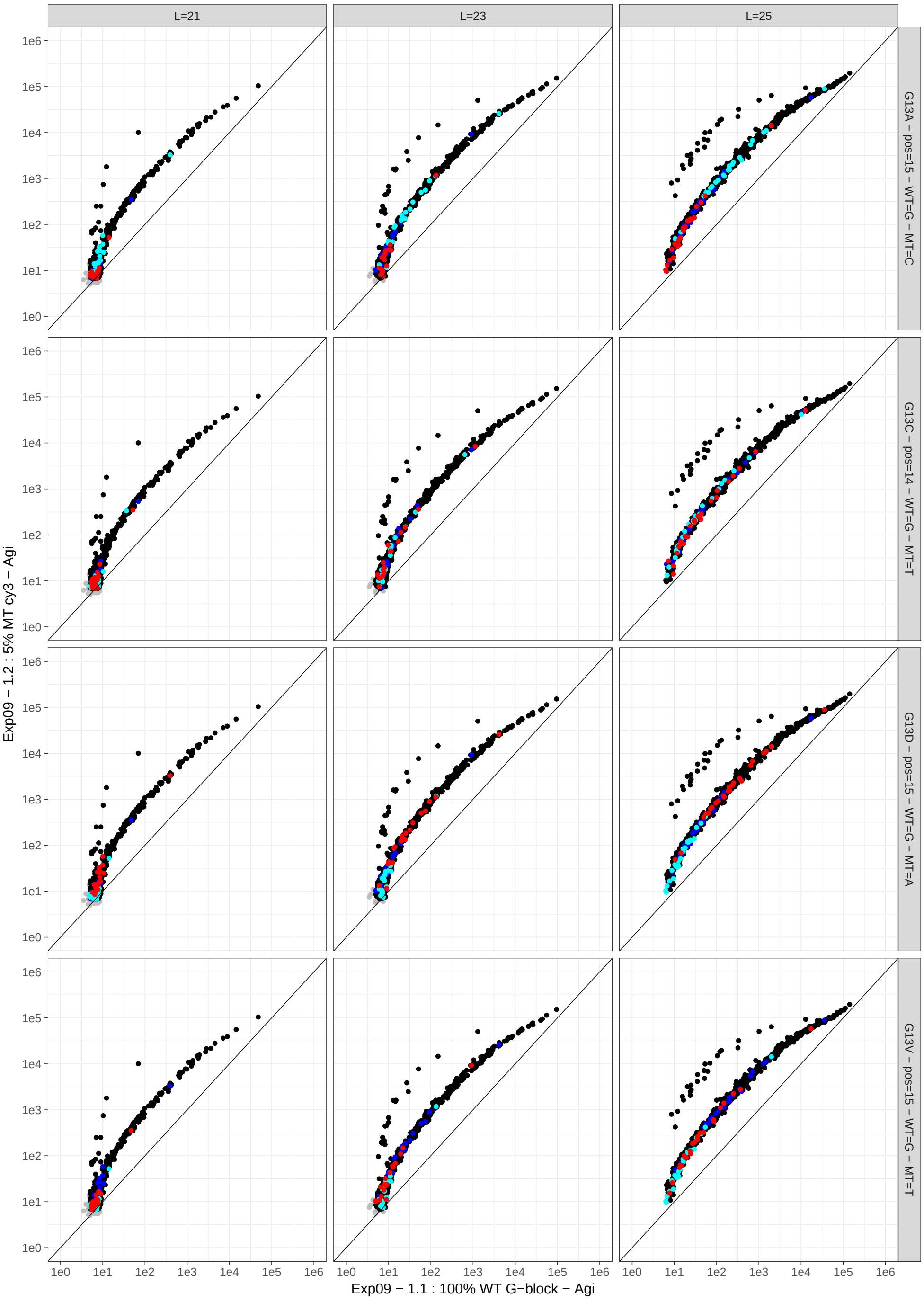

L=21
L=23
L=25
G13A − pos=15 − WT=G − MT=C
G13C − pos=14 − WT=G − MT=T
G13D − pos=15 − WT=G − MT=A
G13V − pos=15 − WT=G − MT=T
Exp09 − 1.2 : 5% MT cy3 − Agi
Exp09 − 1.1 : 100% WT G−block − Agi
1e0
1e1
1e2
1e3
1e4
1e5
1e6

## Experiment 14, G12V

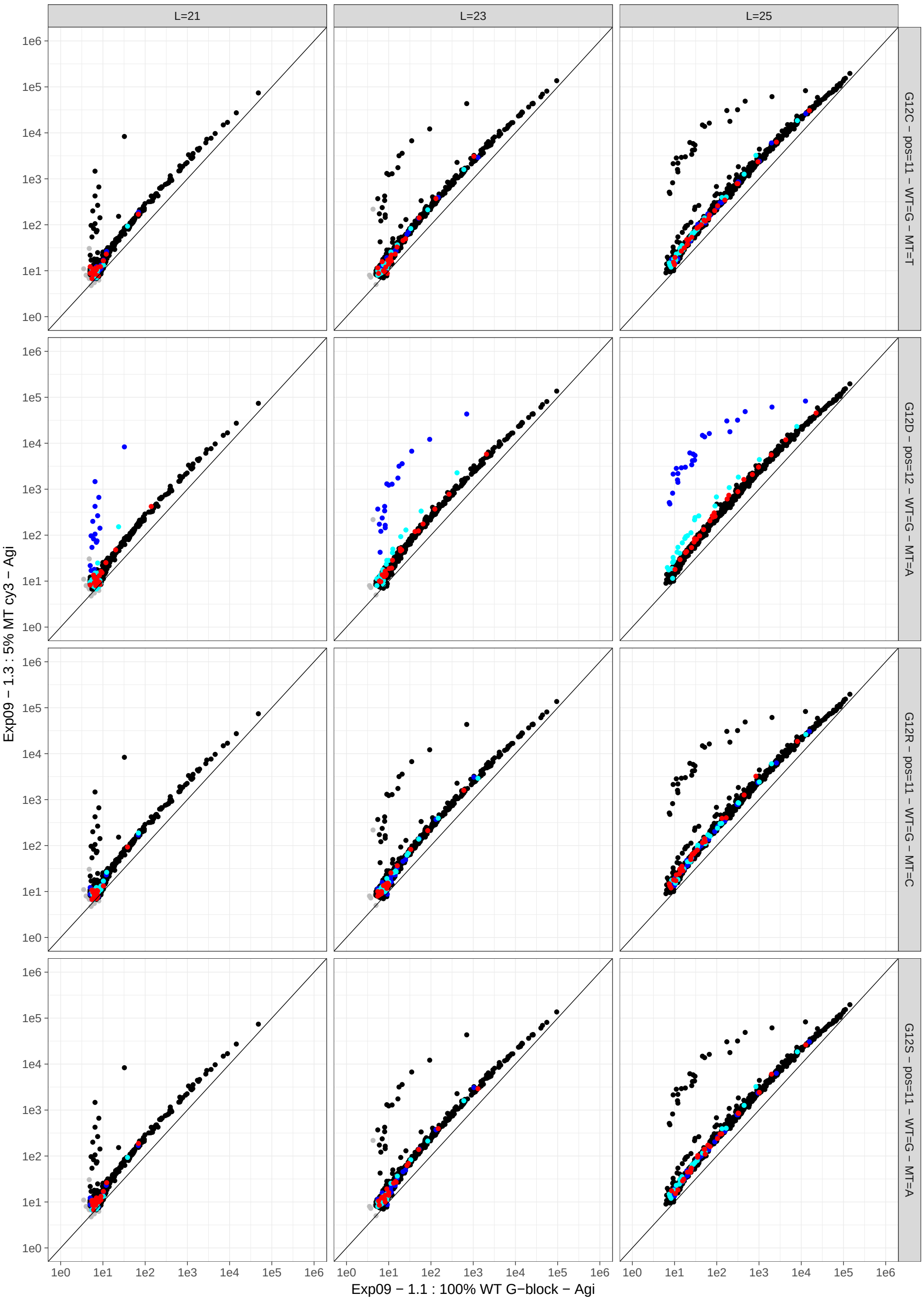

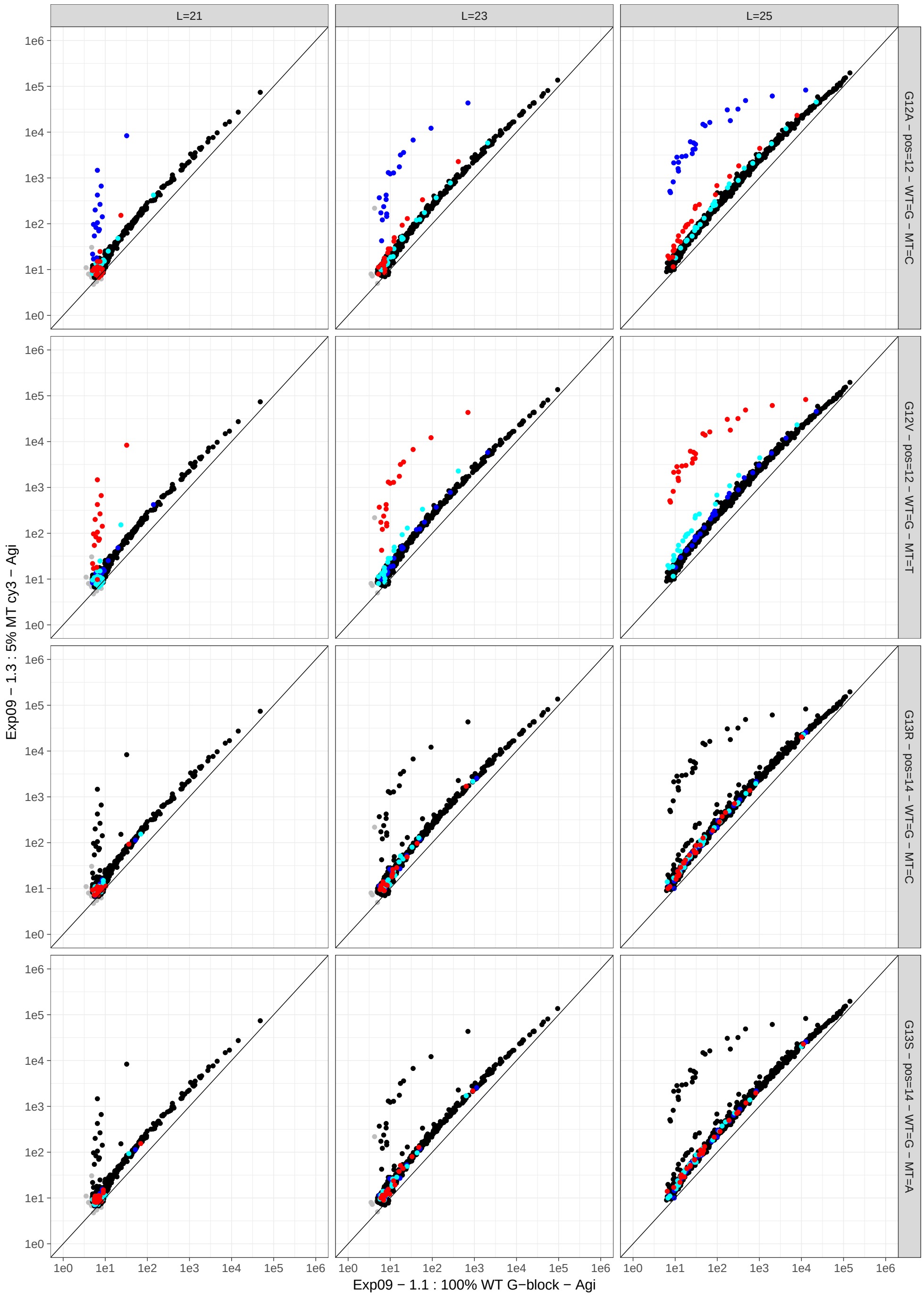

L=21
L=23
L=25
G12A – pos=12 – WT=G – MT=C
G12V – pos=12 – WT=G – MT=T
G13R – pos=14 – WT=G – MT=C
G13S – pos=14 – WT=G – MT=A
Exp09 – 1.3 : 5% MT cy3 – Agi
Exp09 – 1.1 : 100% WT G–block – Agi
1e0
1e1
1e2
1e3
1e4
1e5
1e6

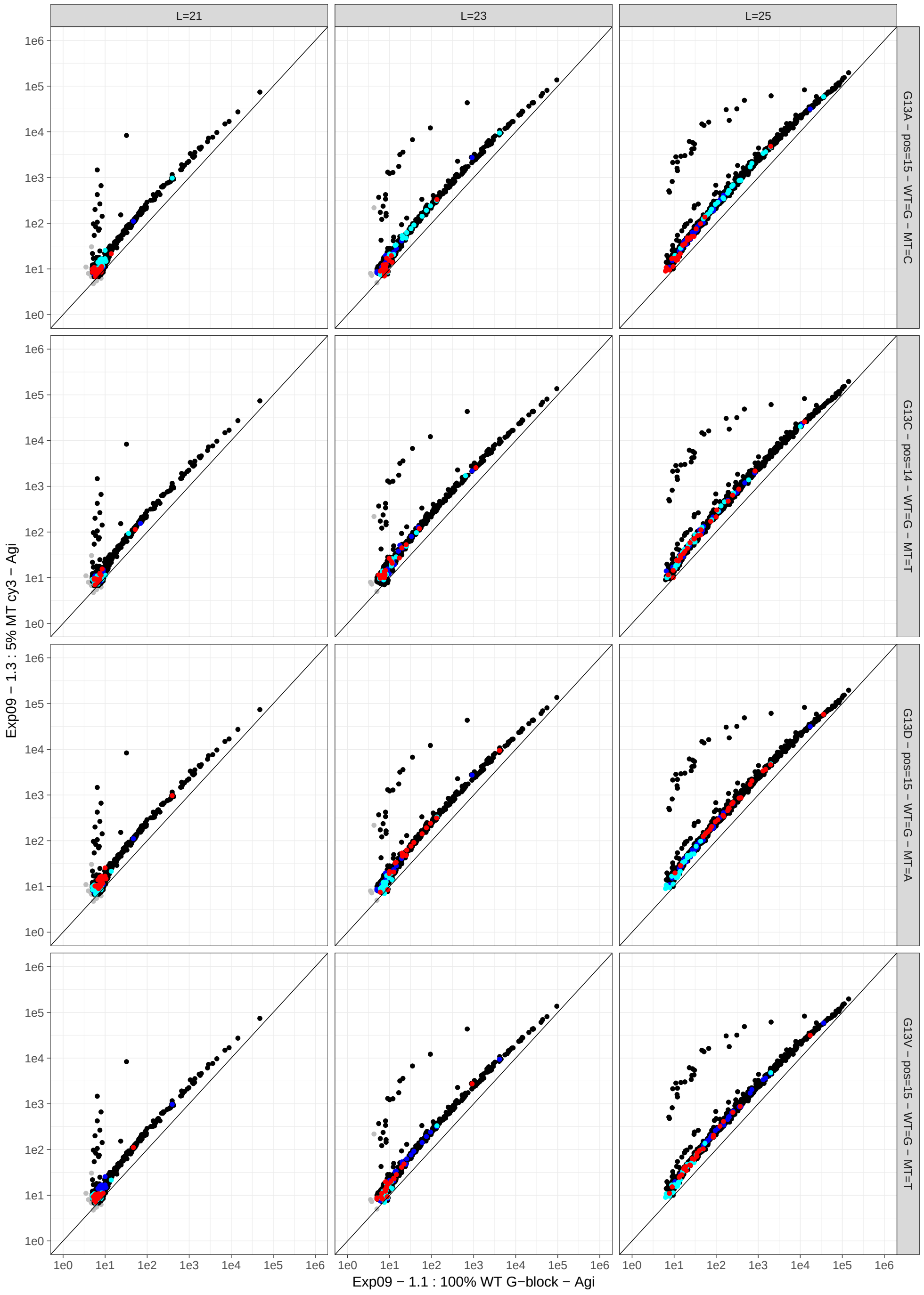

L=21
L=23
L=25
G13A − pos=15 − WT=G − MT=C
G13C − pos=14 − WT=G − MT=T
G13D − pos=15 − WT=G − MT=A
G13V − pos=15 − WT=G − MT=T
Exp09 − 1.3 : 5% MT cy3 − Agi
Exp09 − 1.1 : 100% WT G−block − Agi
1e0
1e1
1e2
1e3
1e4
1e5
1e6

# Experiment 15, G13A

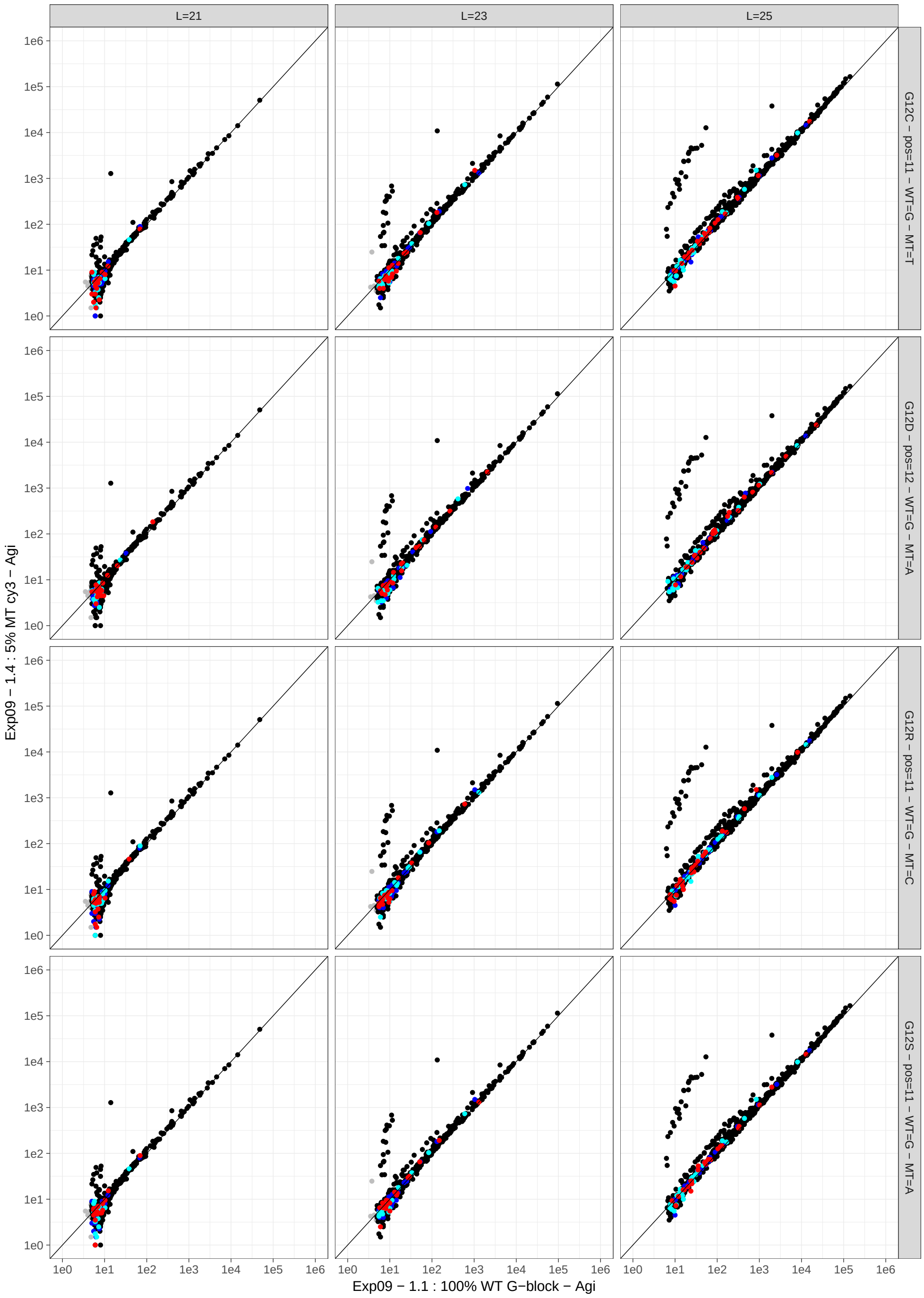

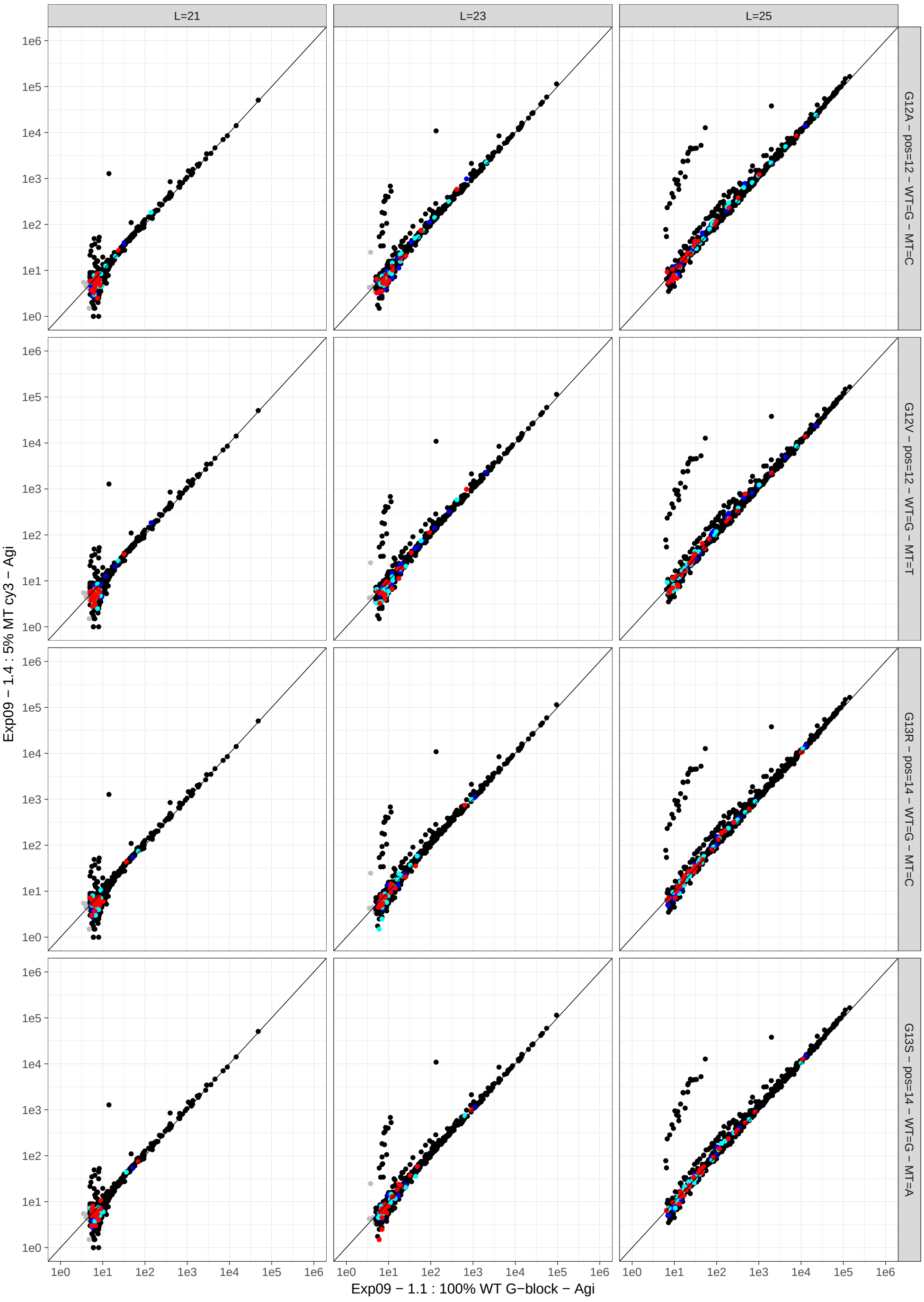

L=21
L=23
L=25
G12A − pos=12 − WT=G − MT=C
G12V − pos=12 − WT=G − MT=T
G13R − pos=14 − WT=G − MT=C
G13S − pos=14 − WT=G − MT=A
Exp09 − 1.4 : 5% MT cy3 − Agi
Exp09 − 1.1 : 100% WT G−block − Agi
1e0
1e1
1e2
1e3
1e4
1e5
1e6

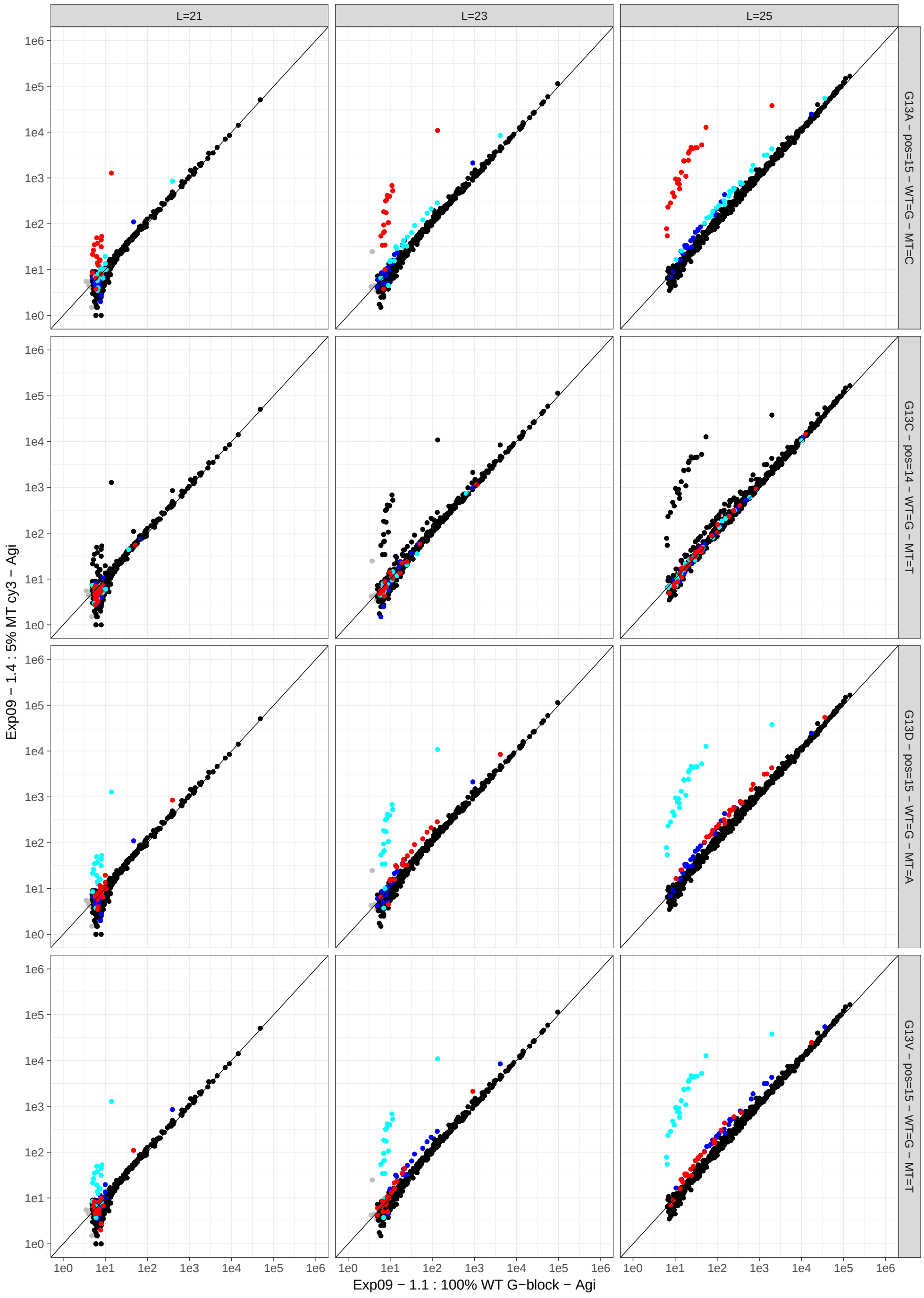

L=21
L=23
L=25
G13A − pos=15 − WT=G − MT=C
G13C − pos=14 − WT=G − MT=T
G13D − pos=15 − WT=G − MT=A
G13V − pos=15 − WT=G − MT=T
Exp09 − 1.4 : 5% MT cy3 − Agi
Exp09 − 1.1 : 100% WT G−block − Agi
1e0
1e1
1e2
1e3
1e4
1e5
1e6

# Experiment 16, G13V

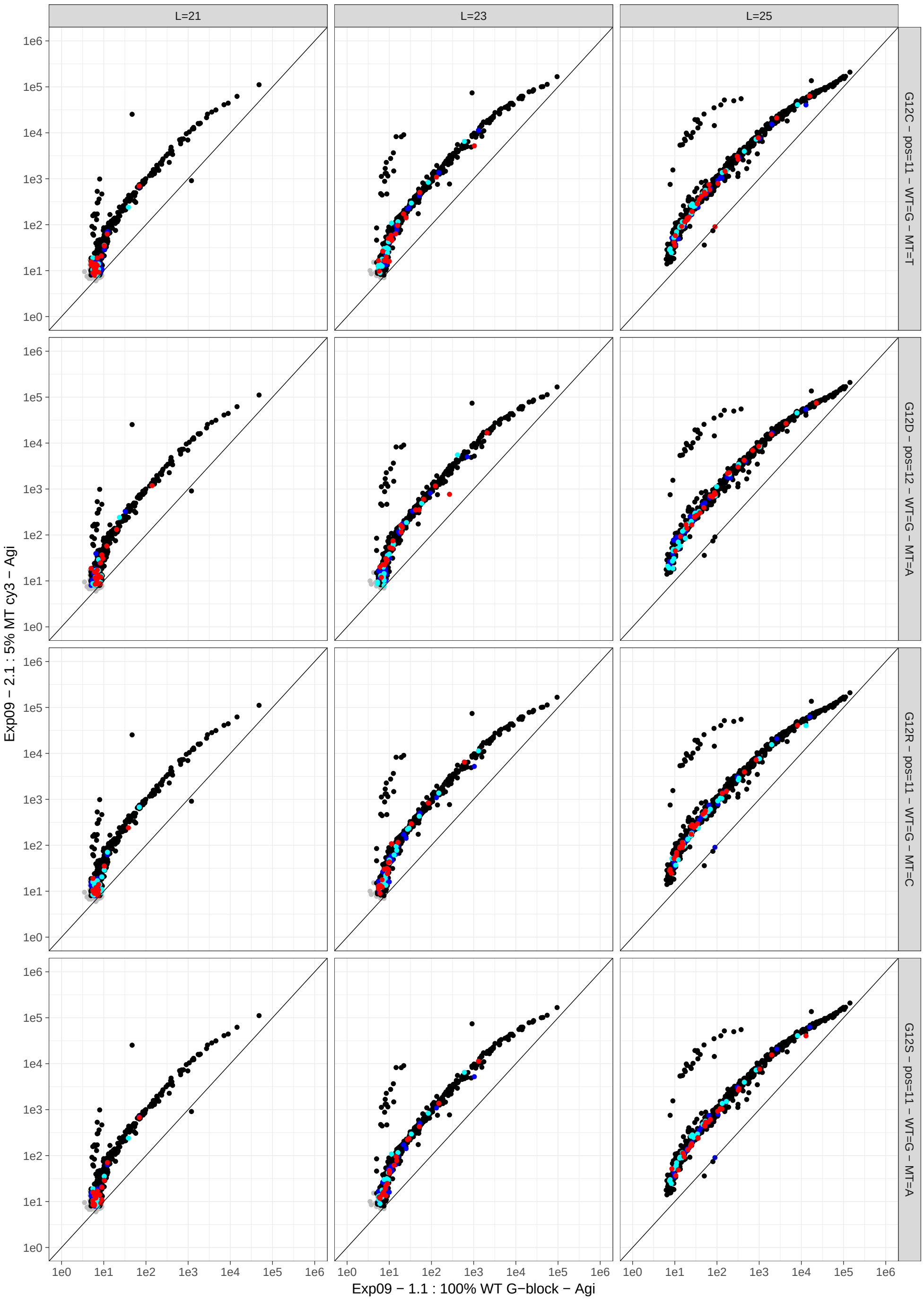

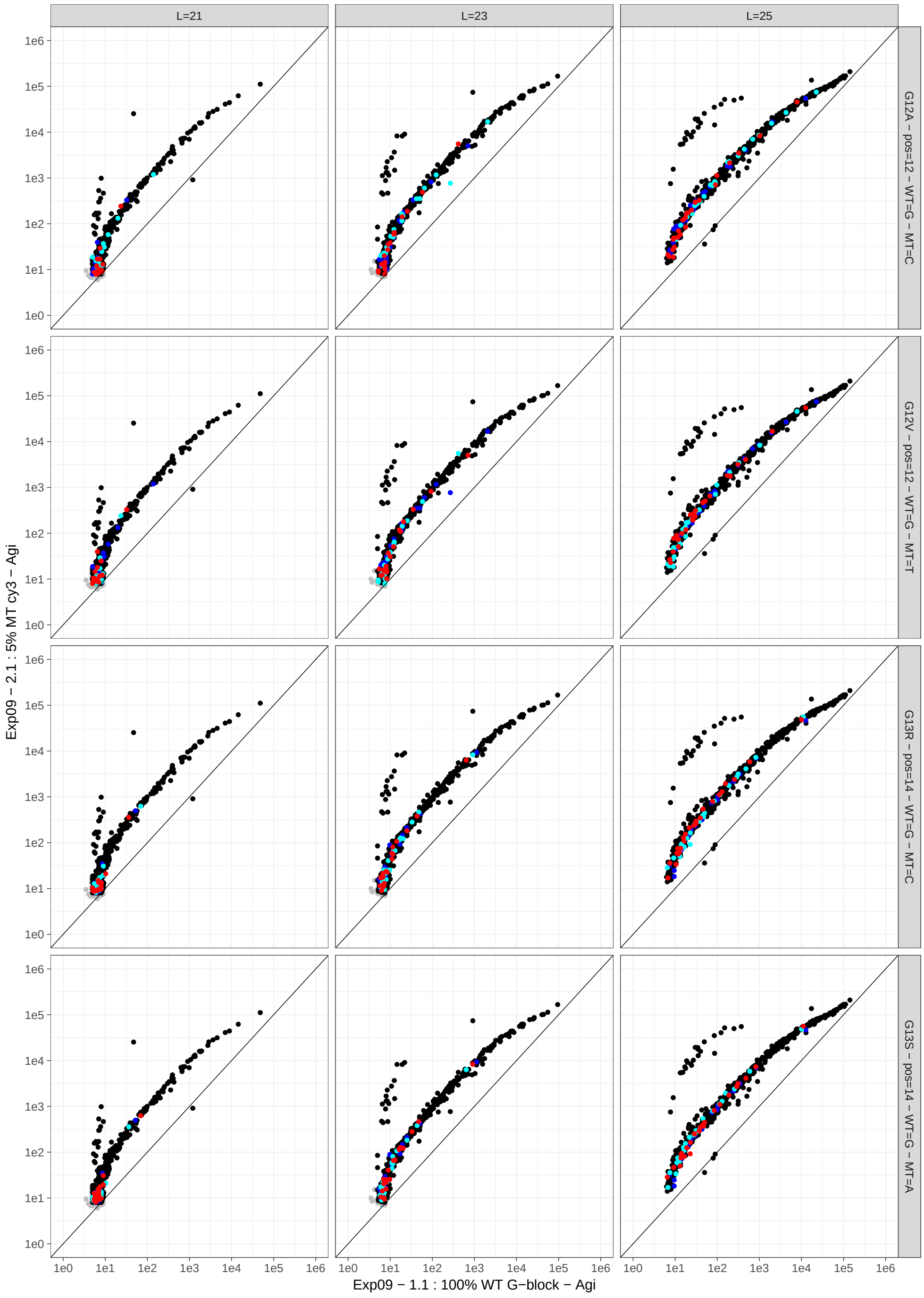

L=21
L=23
L=25
G12A − pos=12 − WT=G − MT=C
G12V − pos=12 − WT=G − MT=T
G13R − pos=14 − WT=G − MT=C
G13S − pos=14 − WT=G − MT=A
1e0
1e1
1e2
1e3
1e4
1e5
1e6
Exp09 − 2.1 : 5% MT cy3 − Agi
Exp09 − 1.1 : 100% WT G−block − Agi

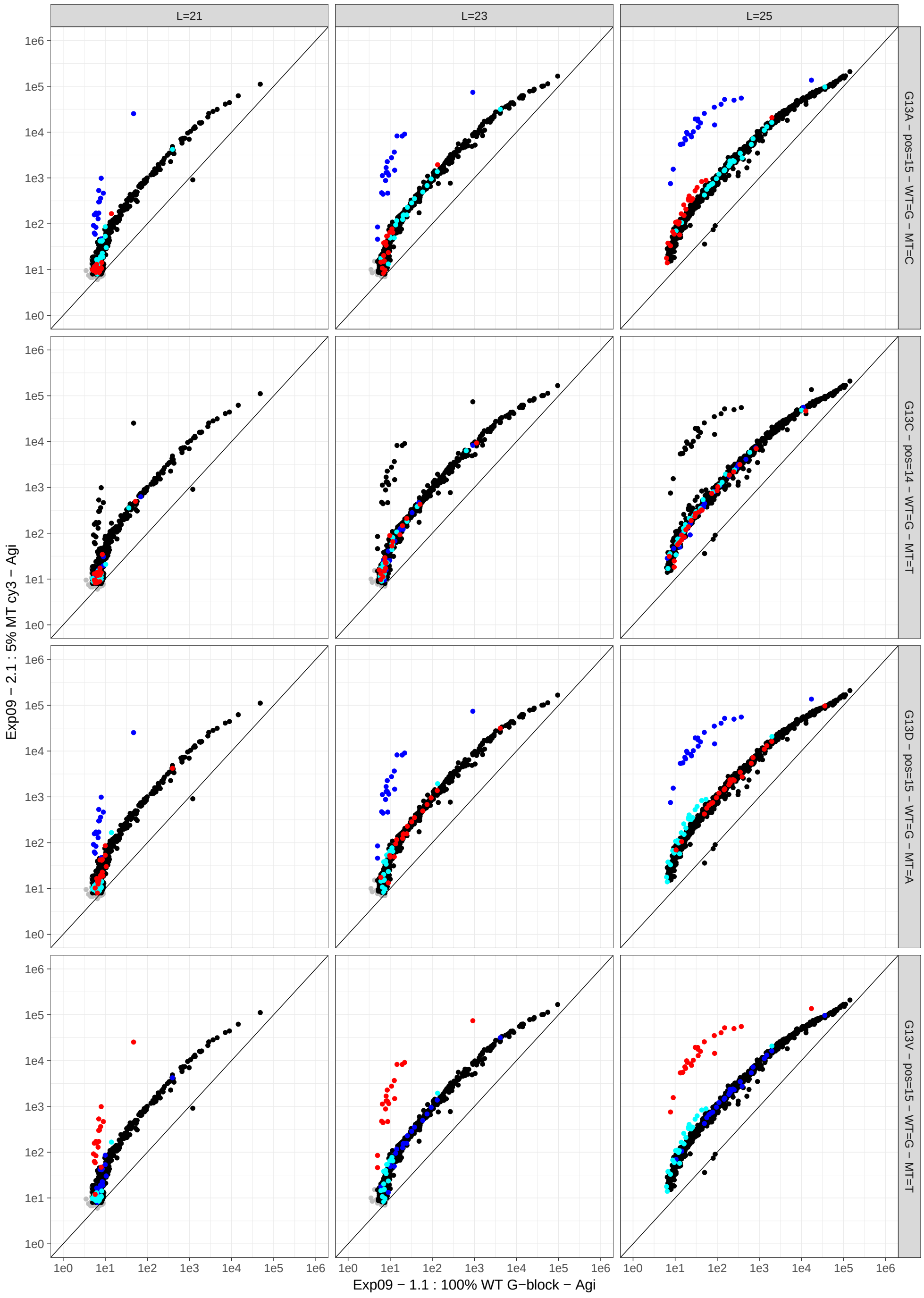
L=21
L=23
L=25
G13A − pos=15 − WT=G − MT=C
G13C − pos=14 − WT=G − MT=T
G13D − pos=15 − WT=G − MT=A
G13V − pos=15 − WT=G − MT=T
1e0
1e1
1e2
1e3
1e4
1e5
1e6
Exp09 − 2.1 : 5% MT cy3 − Agi
Exp09 − 1.1 : 100% WT G−block − Agi

## Experiment 17, G13R

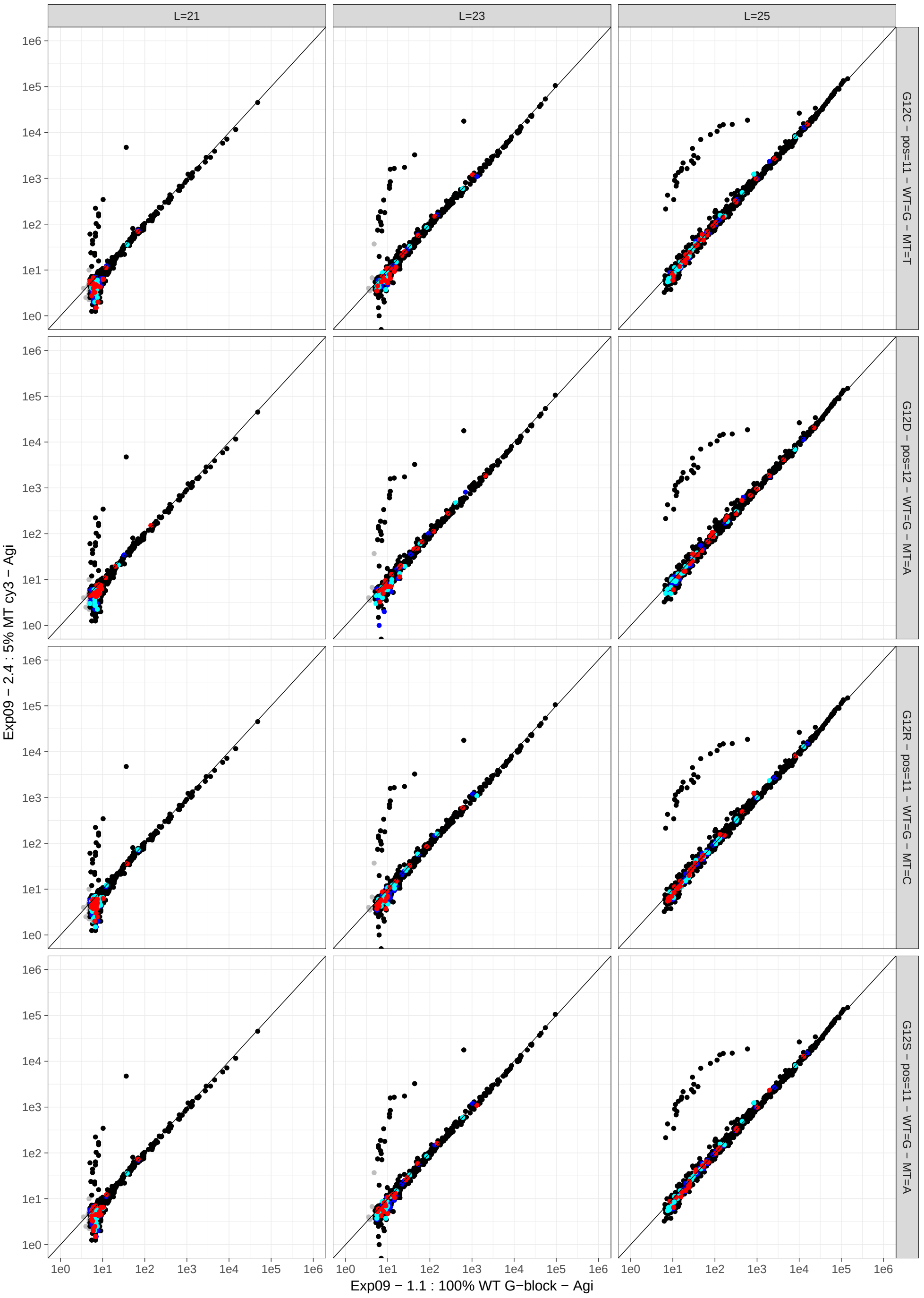

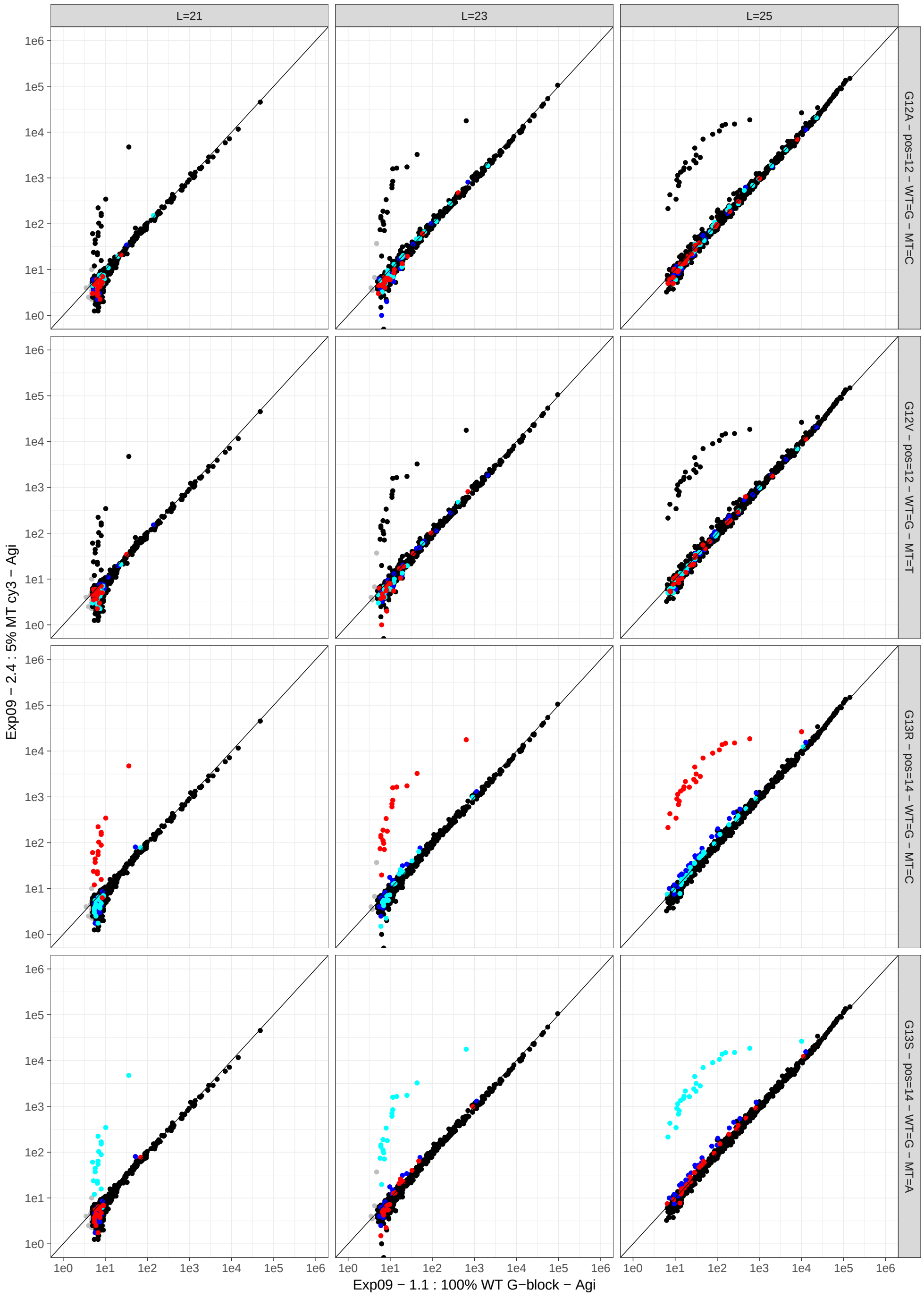

L=21
L=23
L=25
G12A – pos=12 – WT=G – MT=C
G12V – pos=12 – WT=G – MT=T
G13R – pos=14 – WT=G – MT=C
G13S – pos=14 – WT=G – MT=A
1e0
1e1
1e2
1e3
1e4
1e5
1e6
Exp09 – 2.4 : 5% MT cy3 – Agi
Exp09 – 1.1 : 100% WT G−block – Agi

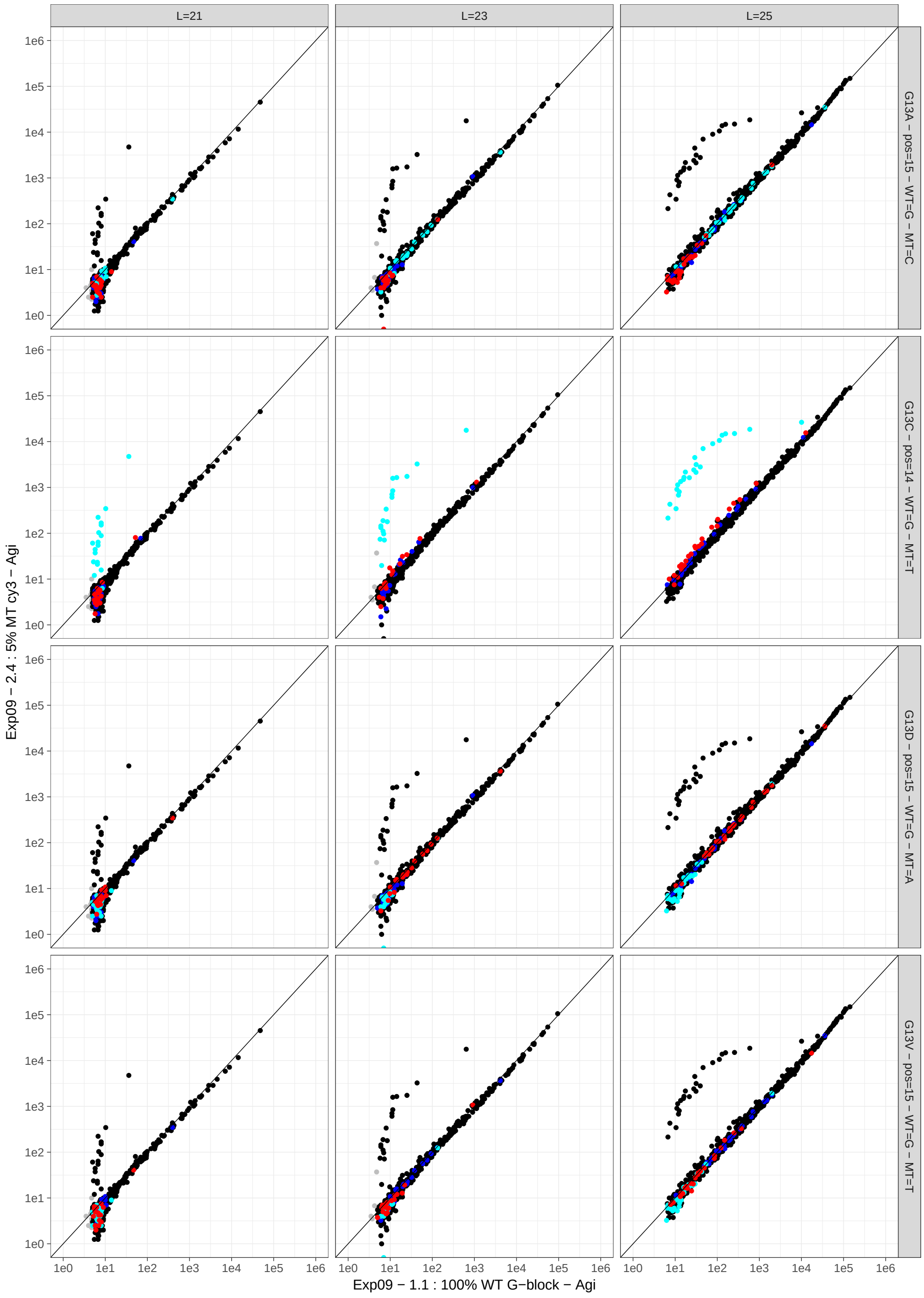

L=21
L=23
L=25
G13A − pos=15 − WT=G − MT=C
G13C − pos=14 − WT=G − MT=T
G13D − pos=15 − WT=G − MT=A
G13V − pos=15 − WT=G − MT=T
1e0
1e1
1e2
1e3
1e4
1e5
1e6
Exp09 − 2.4 : 5% MT cy3 − Agi
Exp09 − 1.1 : 100% WT G−block − Agi

# Experiment 18, G13D

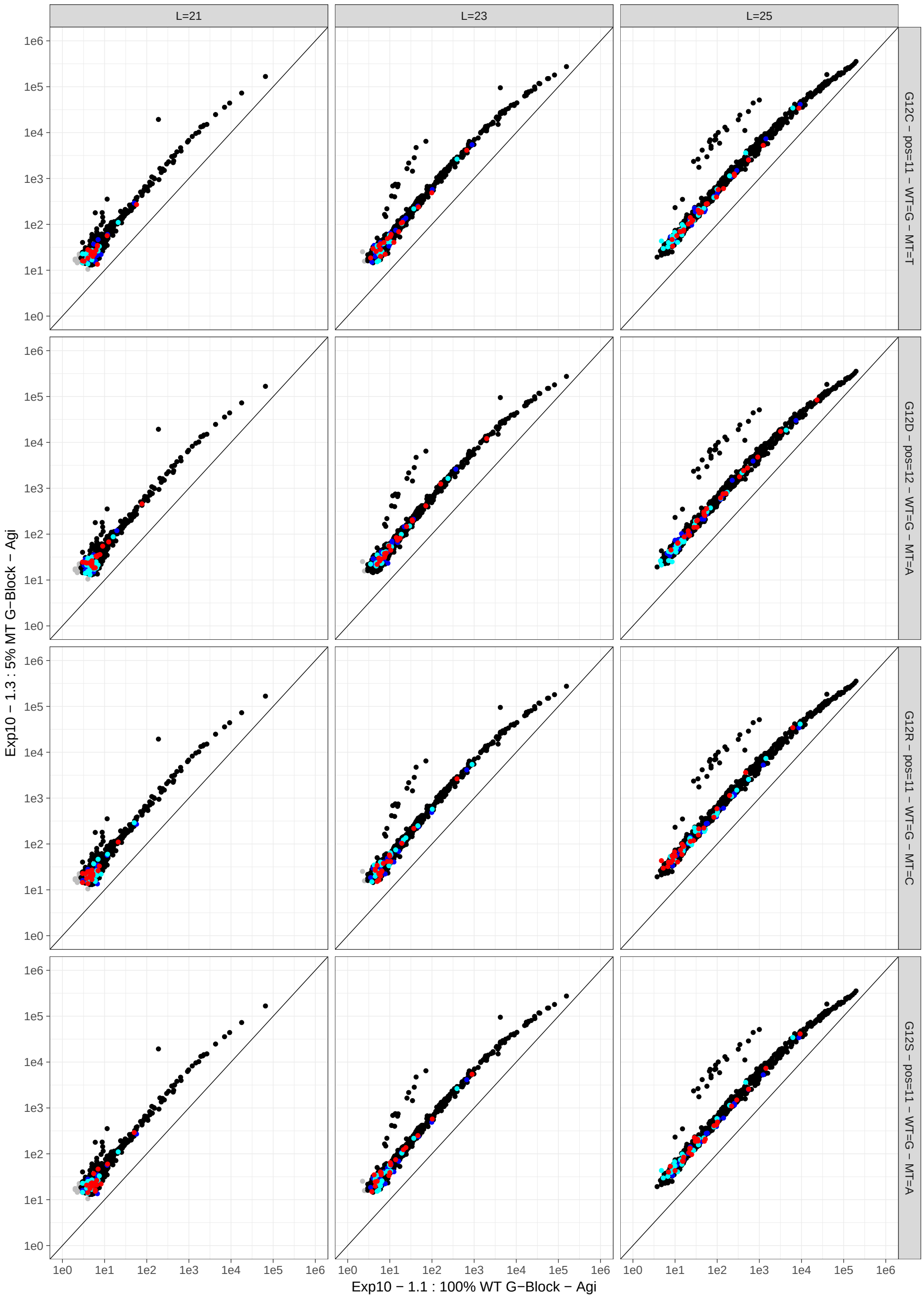

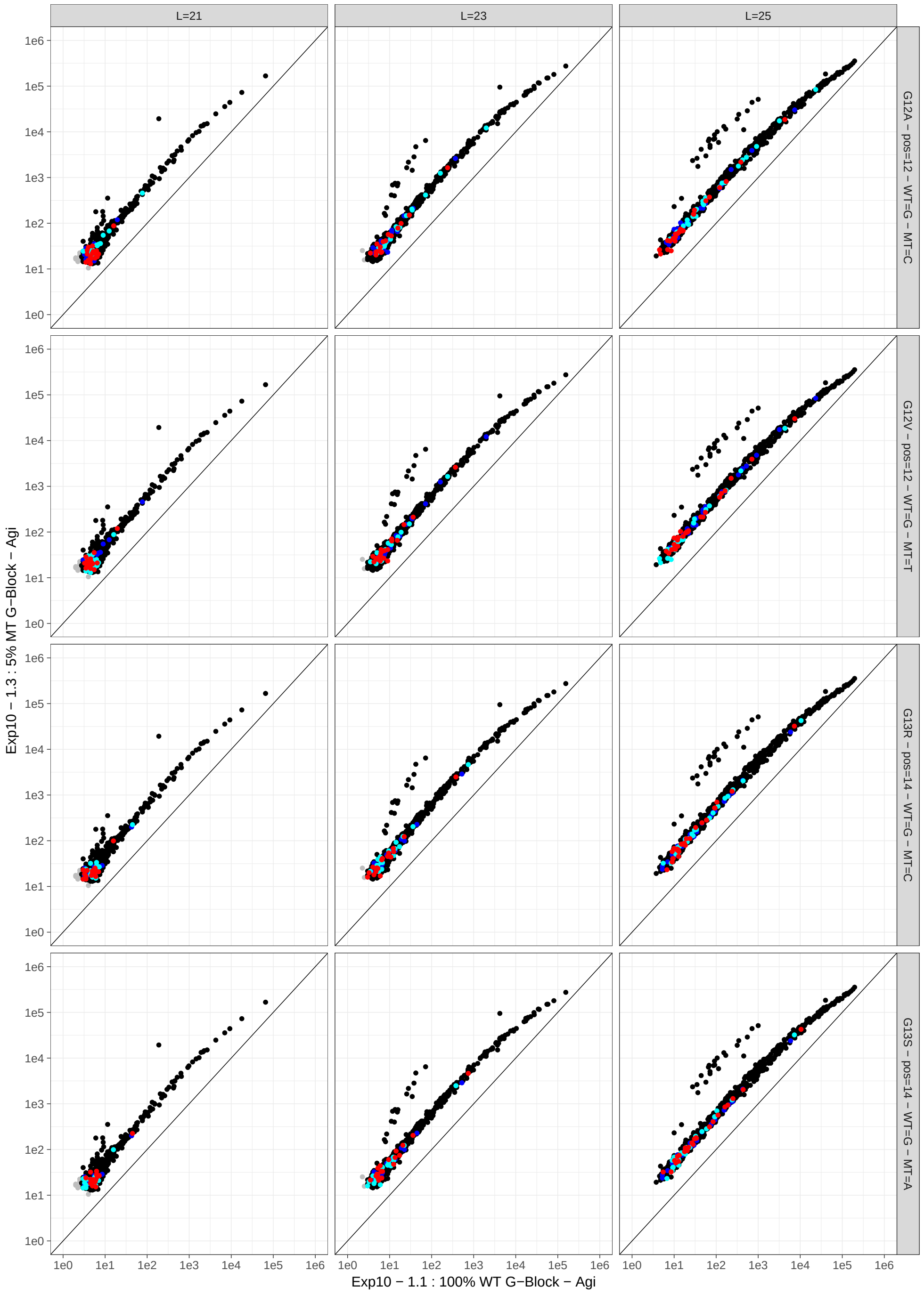
L=21
L=23
L=25
G12A − pos=12 − WT=G − MT=C
G12V − pos=12 − WT=G − MT=T
G13R − pos=14 − WT=G − MT=C
G13S − pos=14 − WT=G − MT=A
1e0
1e1
1e2
1e3
1e4
1e5
1e6
Exp10 − 1.3 : 5% MT G−Block − Agi
Exp10 − 1.1 : 100% WT G−Block − Agi

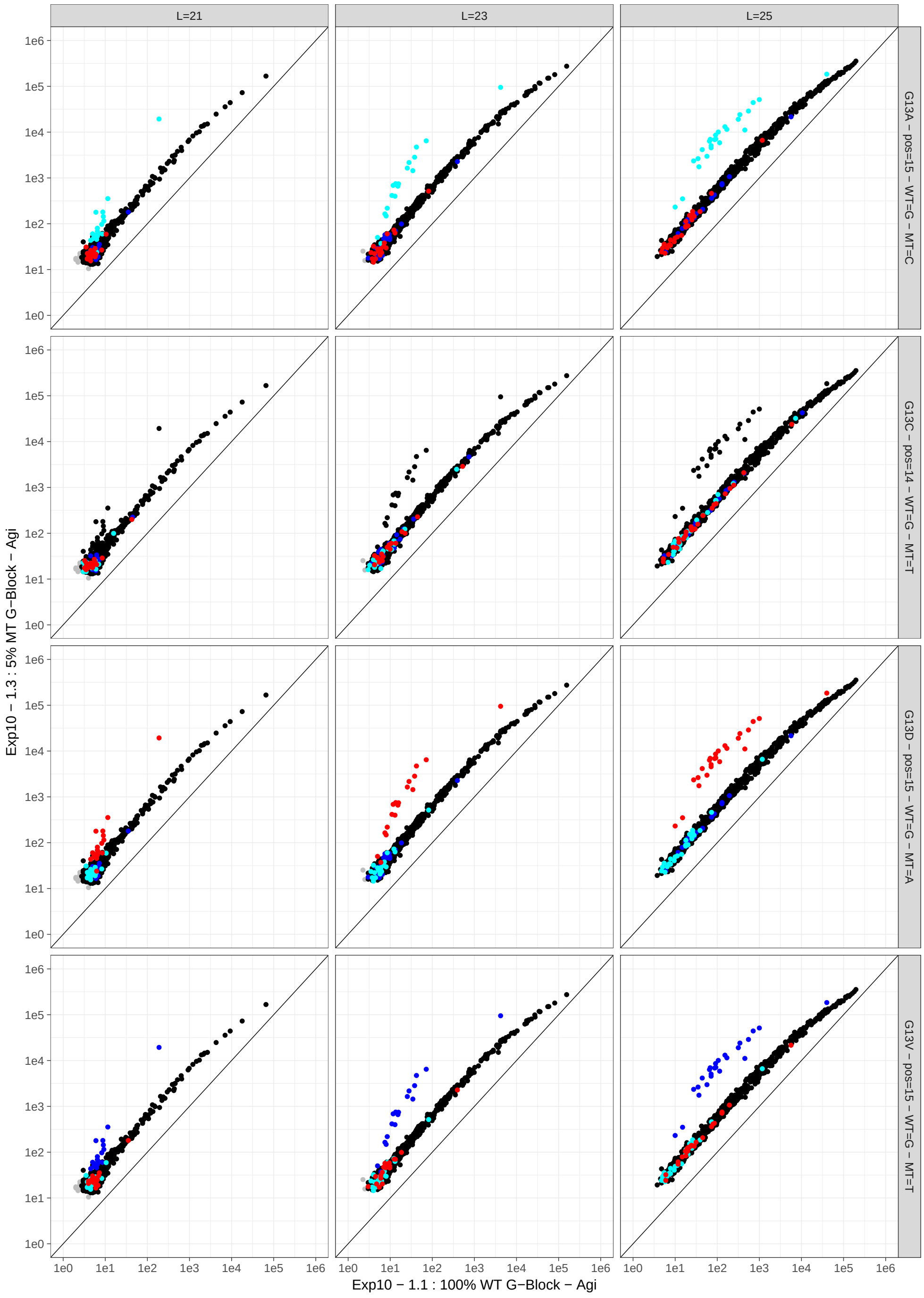

L=21
L=23
L=25
G13A − pos=15 − WT=G − MT=C
G13C − pos=14 − WT=G − MT=T
G13D − pos=15 − WT=G − MT=A
G13V − pos=15 − WT=G − MT=T
1e0
1e1
1e2
1e3
1e4
1e5
1e6
Exp10 − 1.3 : 5% MT G−Block − Agi
Exp10 − 1.1 : 100% WT G−Block − Agi

## Experiment 19, WT

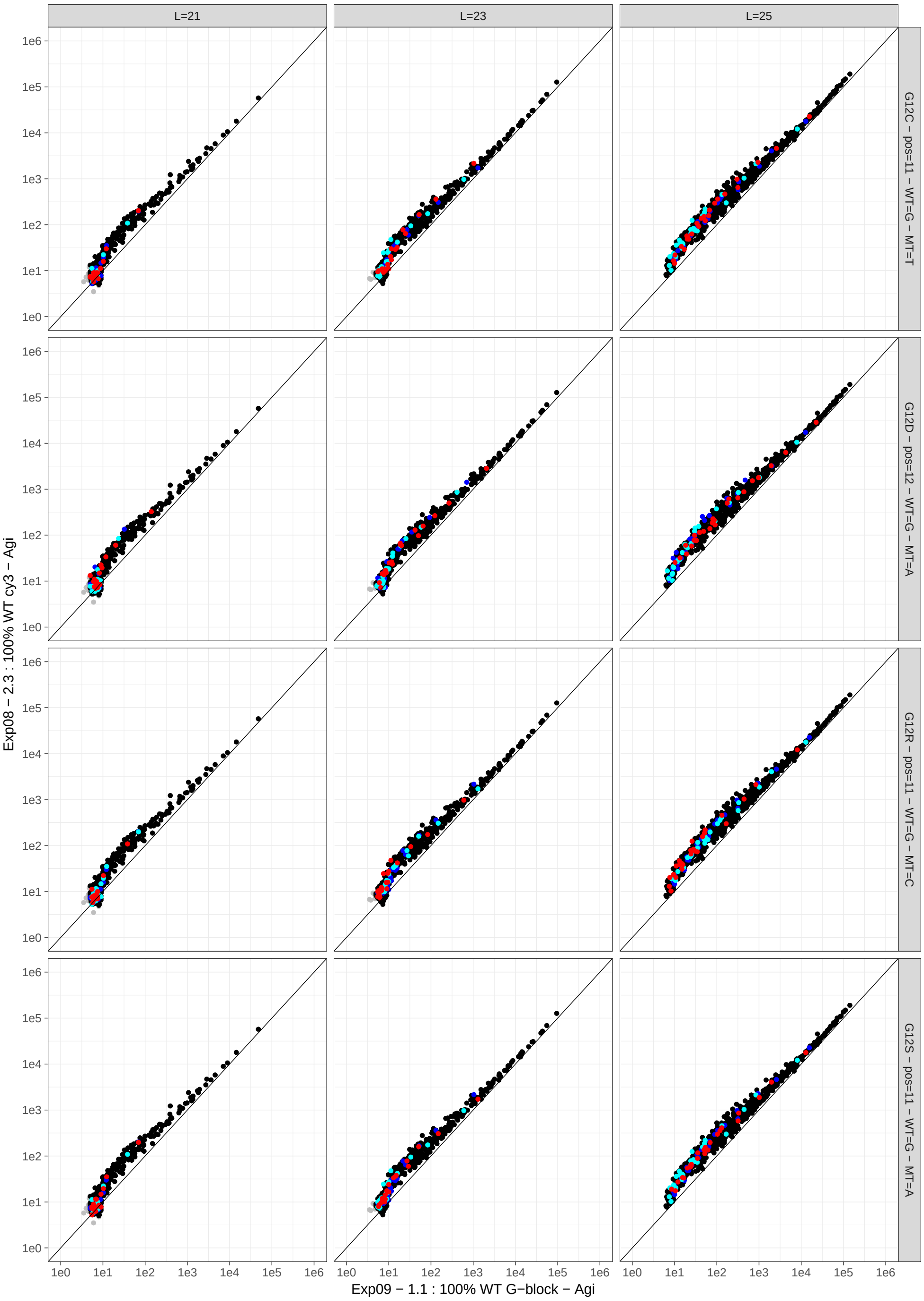

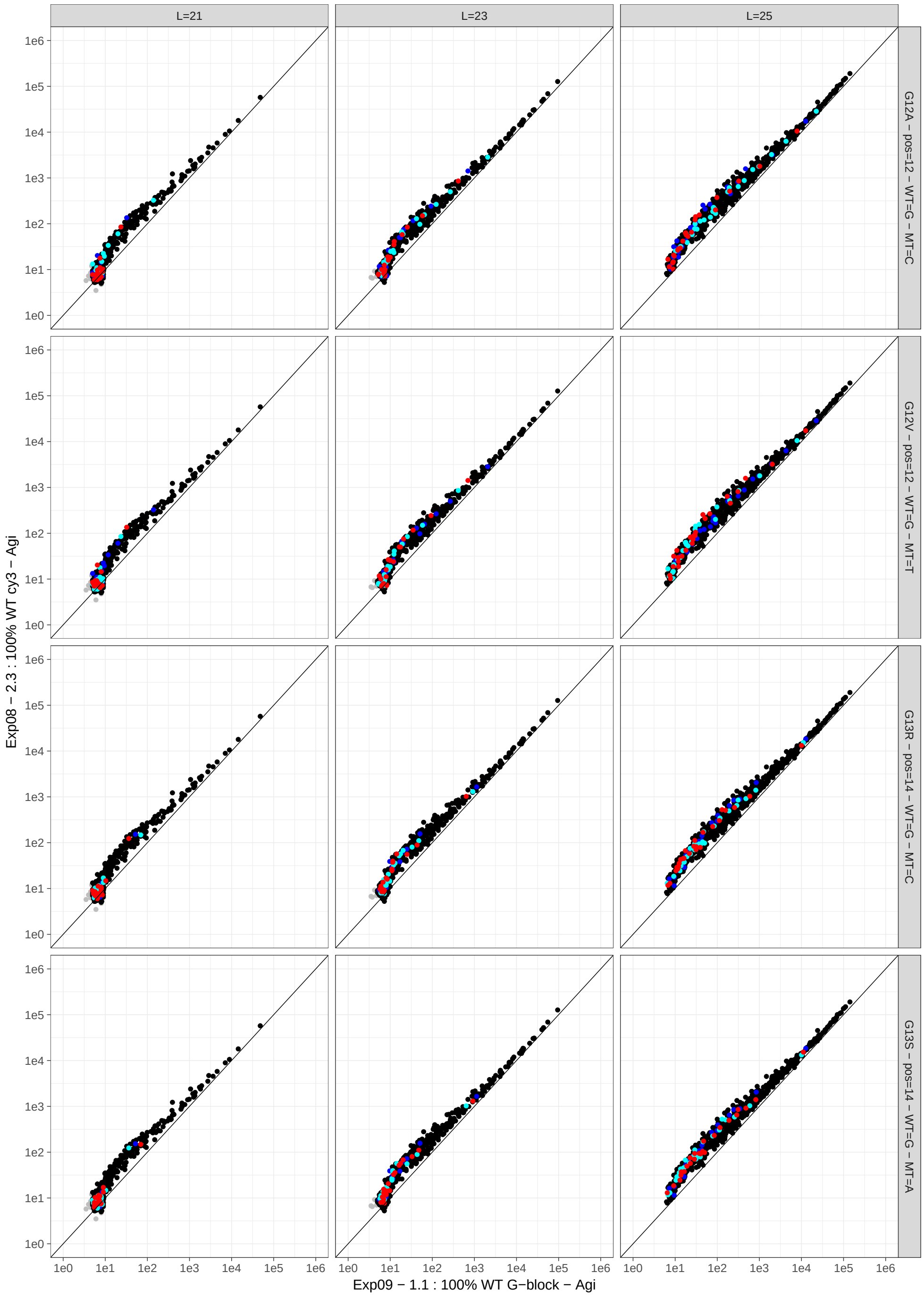
L=21
L=23
L=25
G12A − pos=12 − WT=G − MT=C
G12V − pos=12 − WT=G − MT=T
G13R − pos=14 − WT=G − MT=C
G13S − pos=14 − WT=G − MT=A
1e0
1e1
1e2
1e3
1e4
1e5
1e6
Exp08 − 2.3 : 100% WT cy3 − Agi
Exp09 − 1.1 : 100% WT G−block − Agi

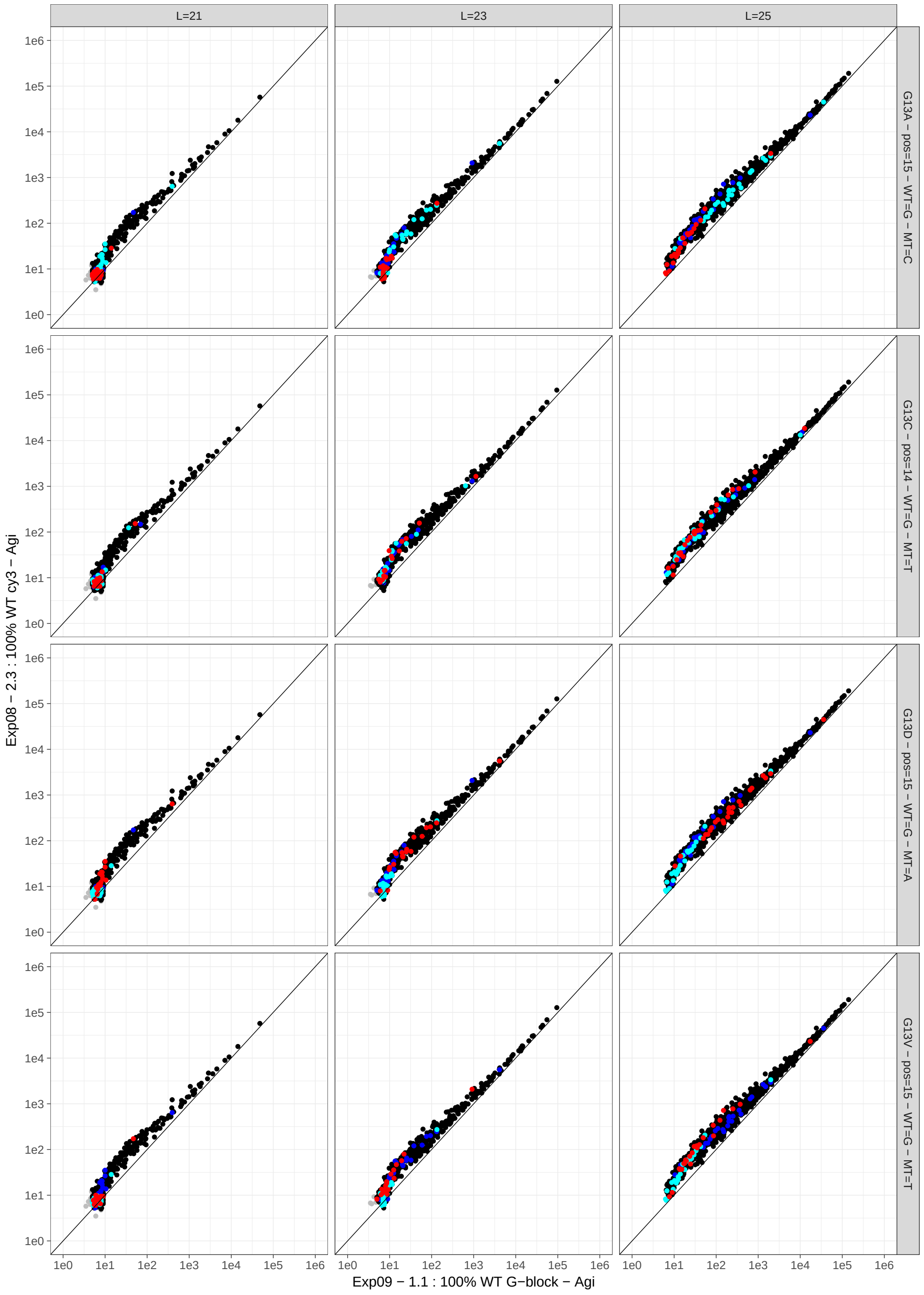

L=21
L=23
L=25
G13A − pos=15 − WT=G − MT=C
G13C − pos=14 − WT=G − MT=T
G13D − pos=15 − WT=G − MT=A
G13V − pos=15 − WT=G − MT=T
Exp08 − 2.3 : 100% WT cy3 − Agi
Exp09 − 1.1 : 100% WT G−block − Agi
1e0
1e1
1e2
1e3
1e4
1e5
1e6

# Experiment set 3: blinded clinical samples

## Experiment 20, G13D

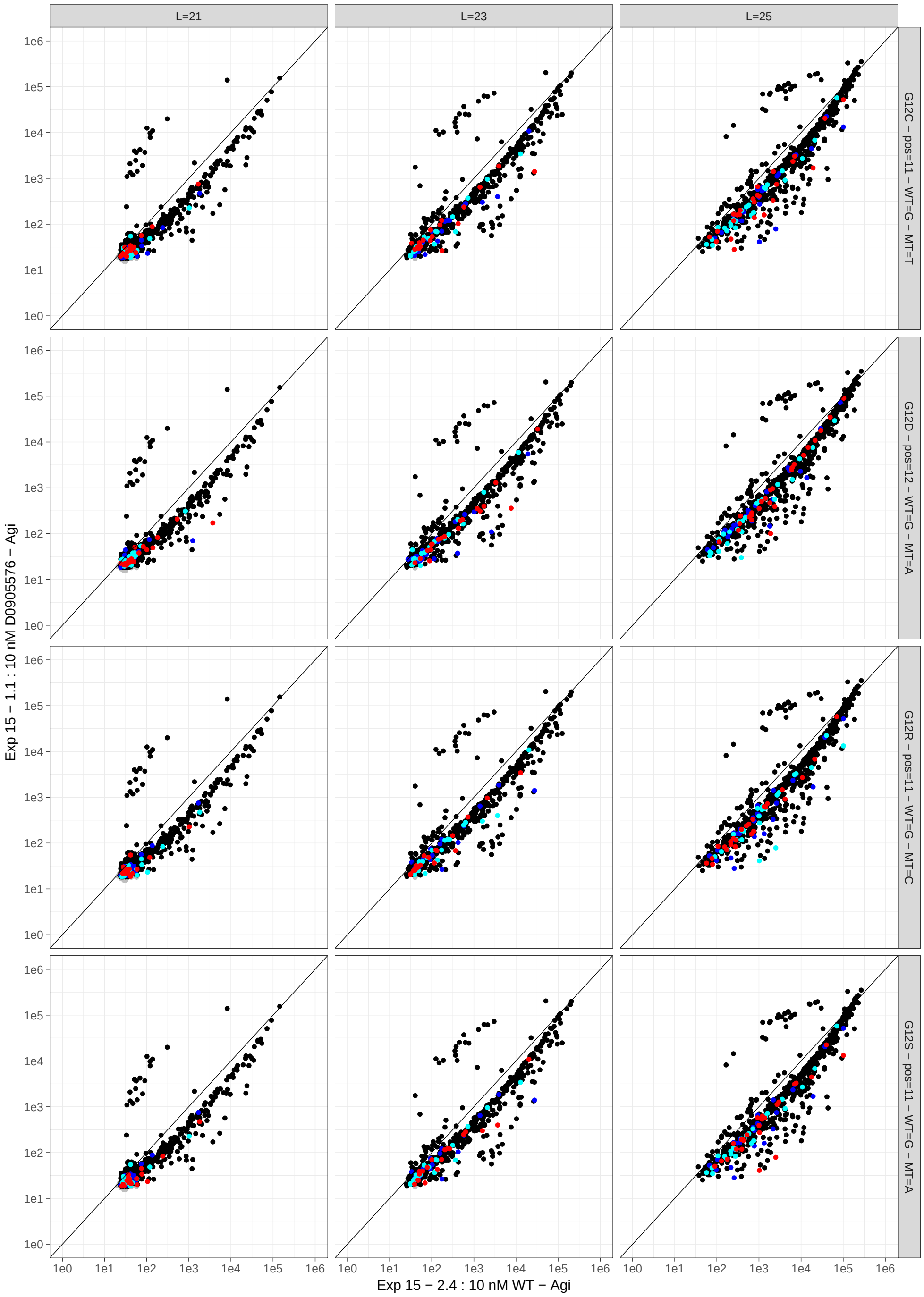

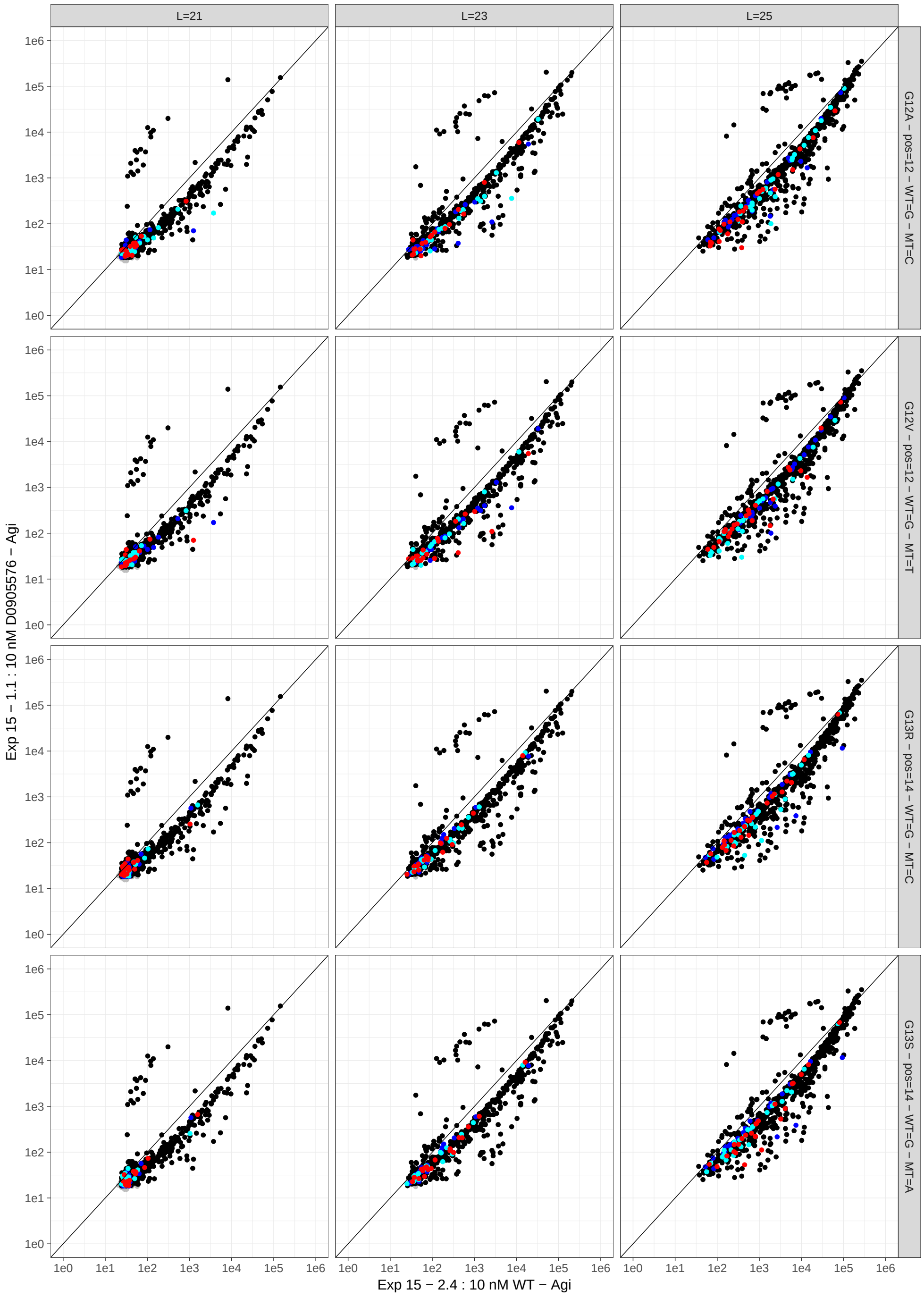

L=21
L=23
L=25
G12A − pos=12 − WT=G − MT=C
G12V − pos=12 − WT=G − MT=T
G13R − pos=14 − WT=G − MT=C
G13S − pos=14 − WT=G − MT=A
Exp 15 − 1.1 : 10 nM D0905576 − Agi
Exp 15 − 2.4 : 10 nM WT − Agi
1e0
1e1
1e2
1e3
1e4
1e5
1e6

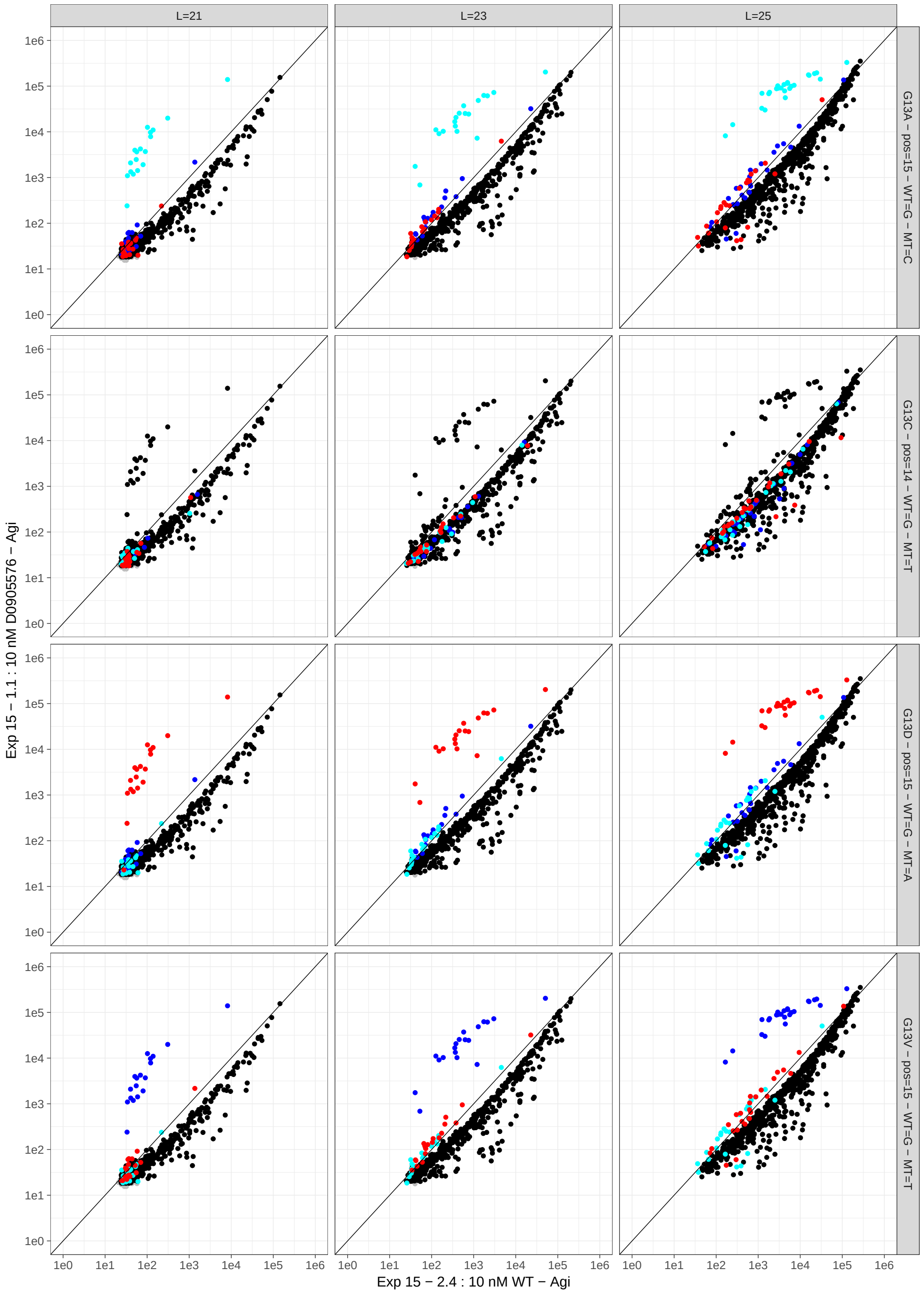
L=21
L=23
L=25
G13A − pos=15 − WT=G − MT=C
G13C − pos=14 − WT=G − MT=T
G13D − pos=15 − WT=G − MT=A
G13V − pos=15 − WT=G − MT=T
1e0
1e1
1e2
1e3
1e4
1e5
1e6
Exp 15 − 1.1 : 10 nM D0905576 − Agi
Exp 15 − 2.4 : 10 nM WT − Agi

# Experiment 21, G12A

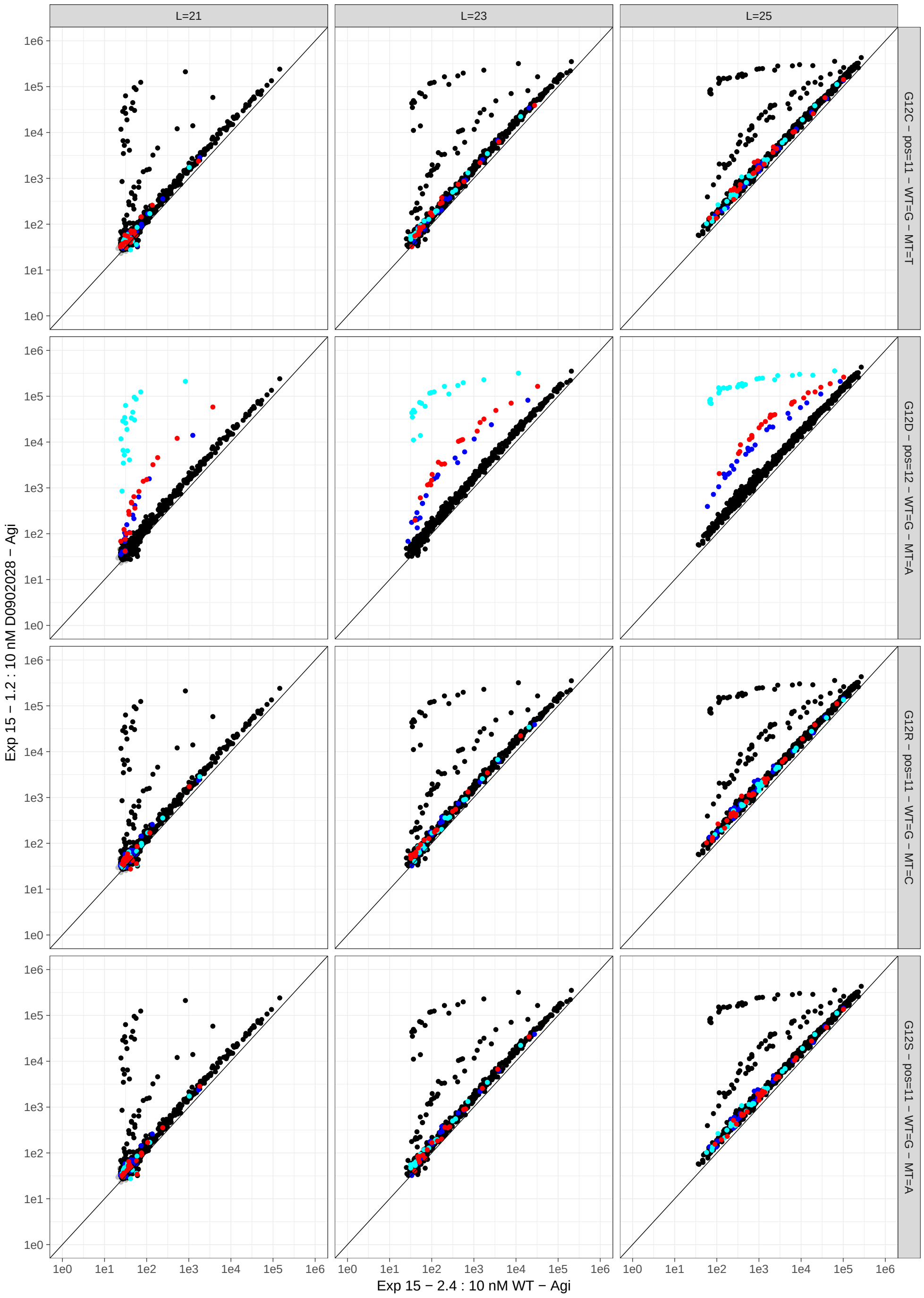

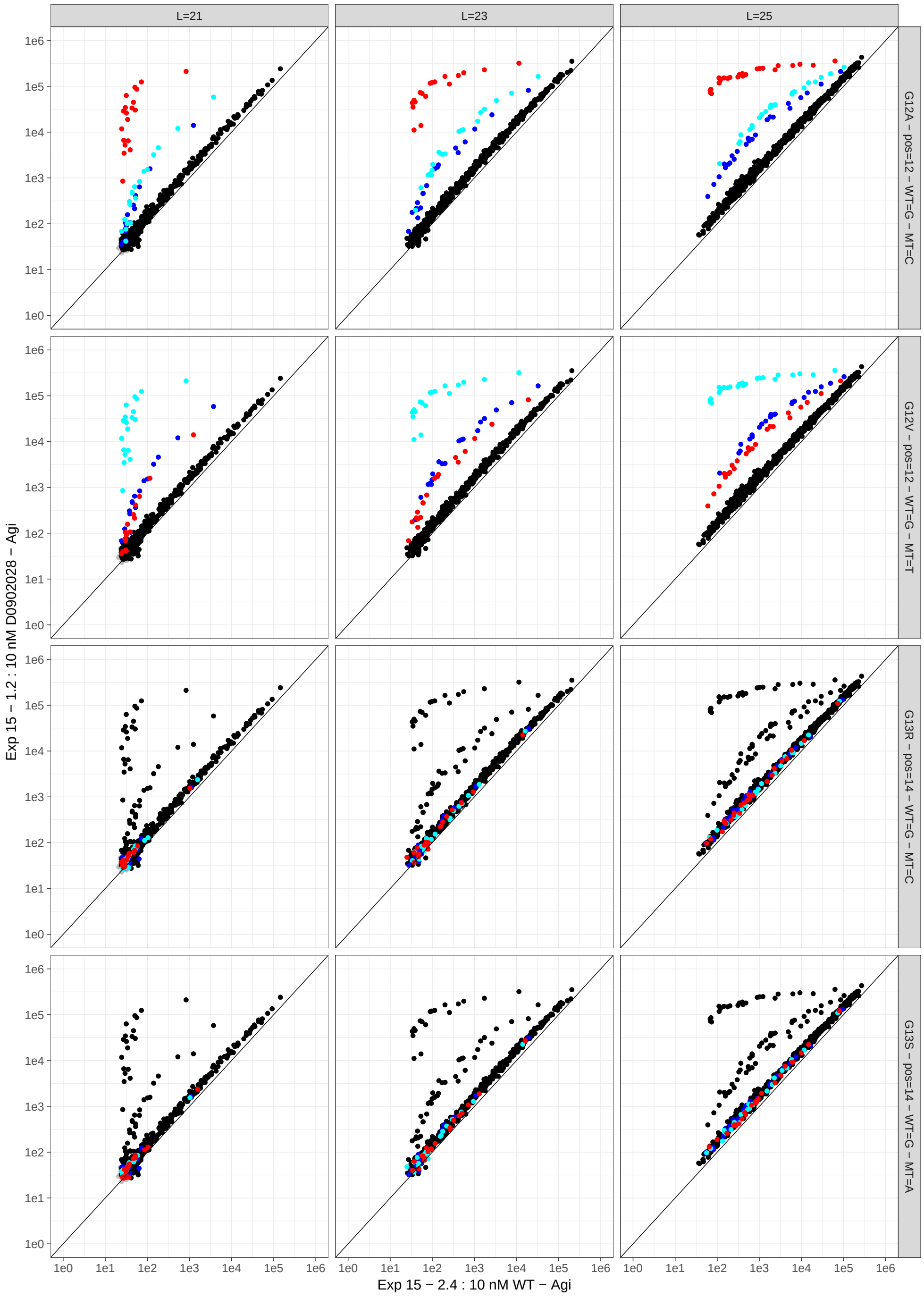

L=21
L=23
L=25
G12A − pos=12 − WT=G − MT=C
G12V − pos=12 − WT=G − MT=T
G13R − pos=14 − WT=G − MT=C
G13S − pos=14 − WT=G − MT=A
1e0
1e1
1e2
1e3
1e4
1e5
1e6
Exp 15 − 1.2 : 10 nM D0902028 − Agi
Exp 15 − 2.4 : 10 nM WT − Agi

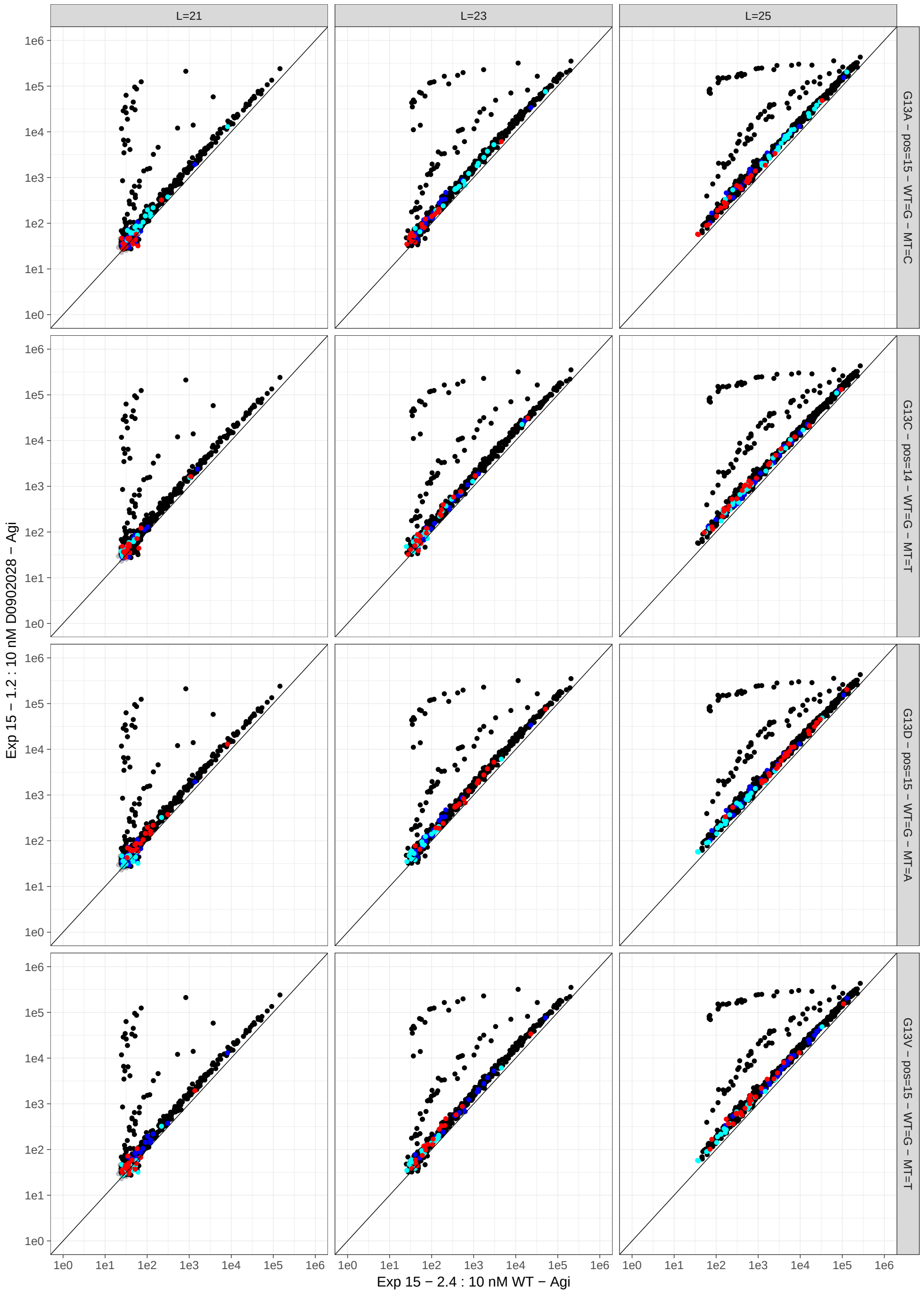
L=21
L=23
L=25
G13A − pos=15 − WT=G − MT=C
G13C − pos=14 − WT=G − MT=T
G13D − pos=15 − WT=G − MT=A
G13V − pos=15 − WT=G − MT=T
Exp 15 − 1.2 : 10 nM D0902028 − Agi
Exp 15 − 2.4 : 10 nM WT − Agi
1e0
1e1
1e2
1e3
1e4
1e5
1e6

# Experiment 22, G12V

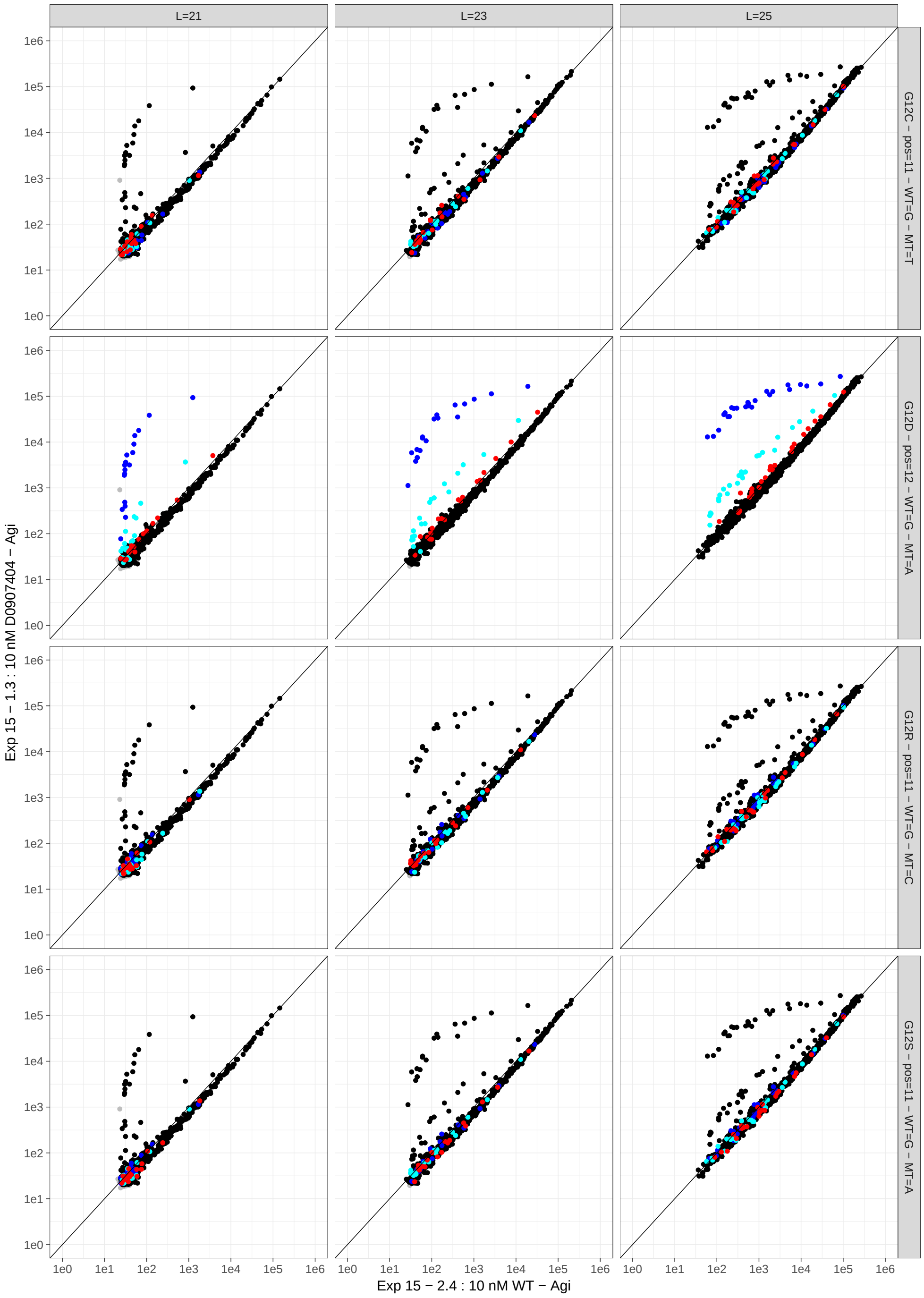

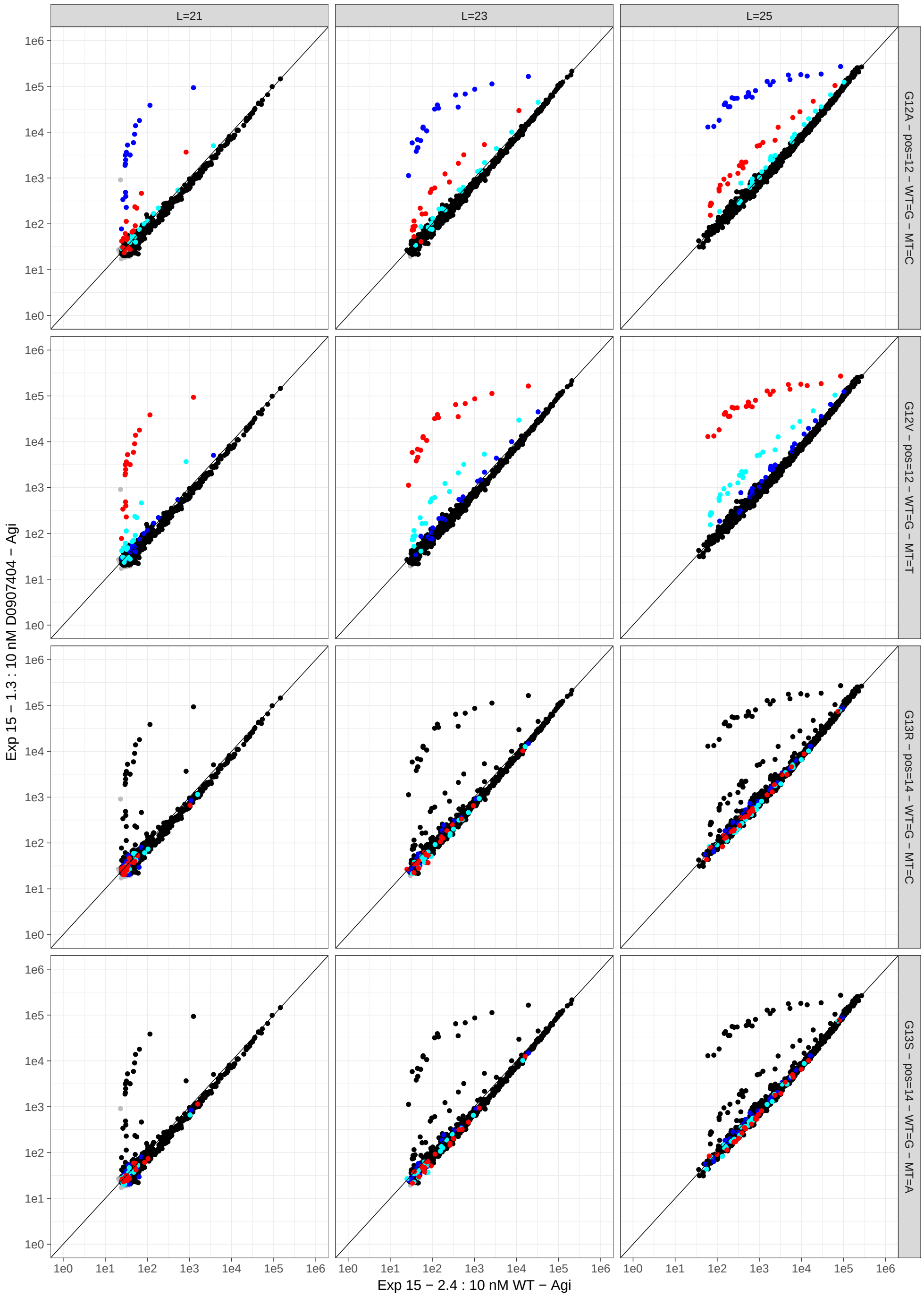

L=21
L=23
L=25
G12A − pos=12 − WT=G − MT=C
G12V − pos=12 − WT=G − MT=T
G13R − pos=14 − WT=G − MT=C
G13S − pos=14 − WT=G − MT=A
1e0
1e1
1e2
1e3
1e4
1e5
1e6
Exp 15 − 1.3 : 10 nM D0907404 − Agi
Exp 15 − 2.4 : 10 nM WT − Agi

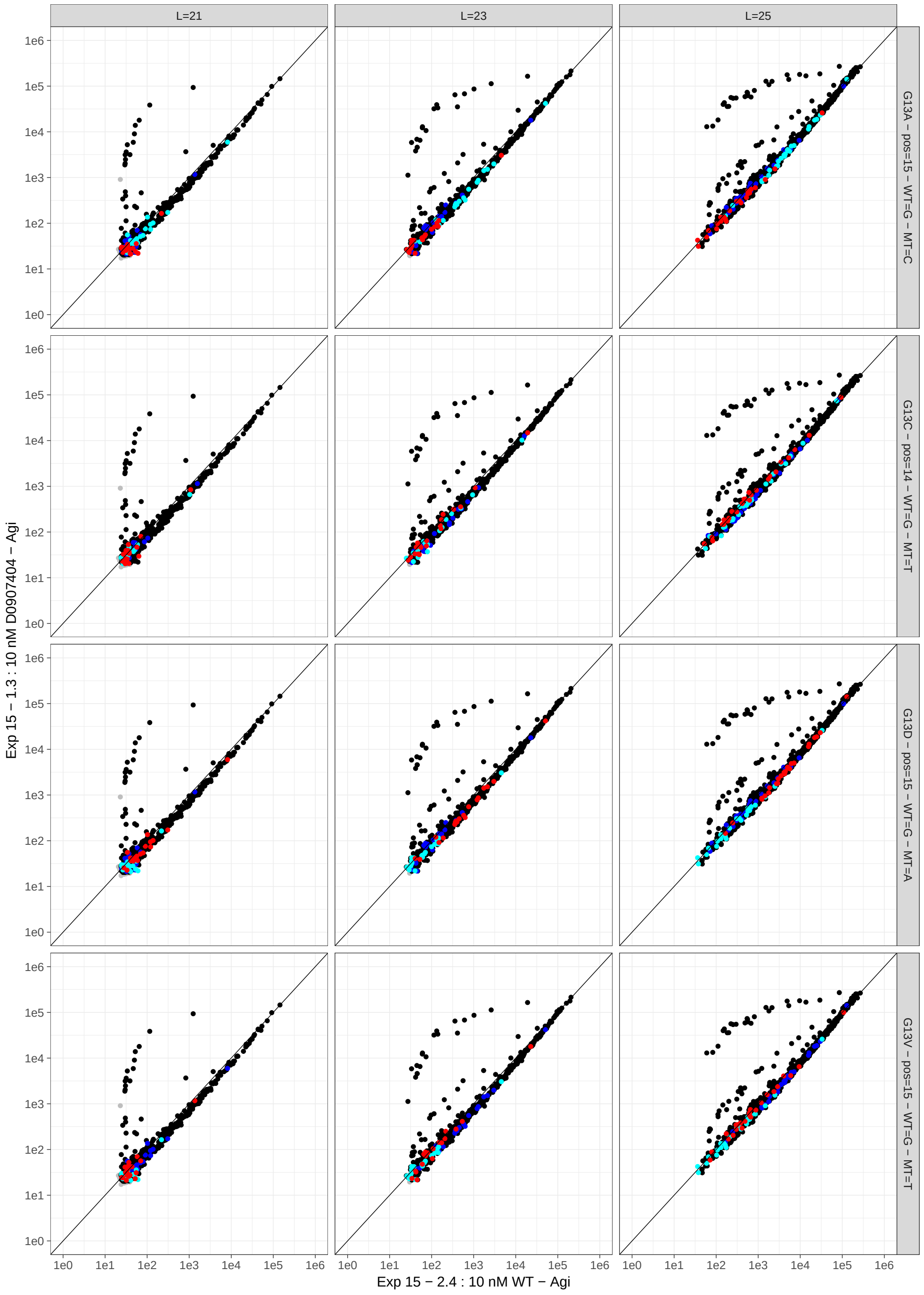

L=21
L=23
L=25
G13A − pos=15 − WT=G − MT=C
G13C − pos=14 − WT=G − MT=T
G13D − pos=15 − WT=G − MT=A
G13V − pos=15 − WT=G − MT=T
1e0
1e1
1e2
1e3
1e4
1e5
1e6
Exp 15 − 1.3 : 10 nM D0907404 − Agi
Exp 15 − 2.4 : 10 nM WT − Agi

## Experiment 23, G12S

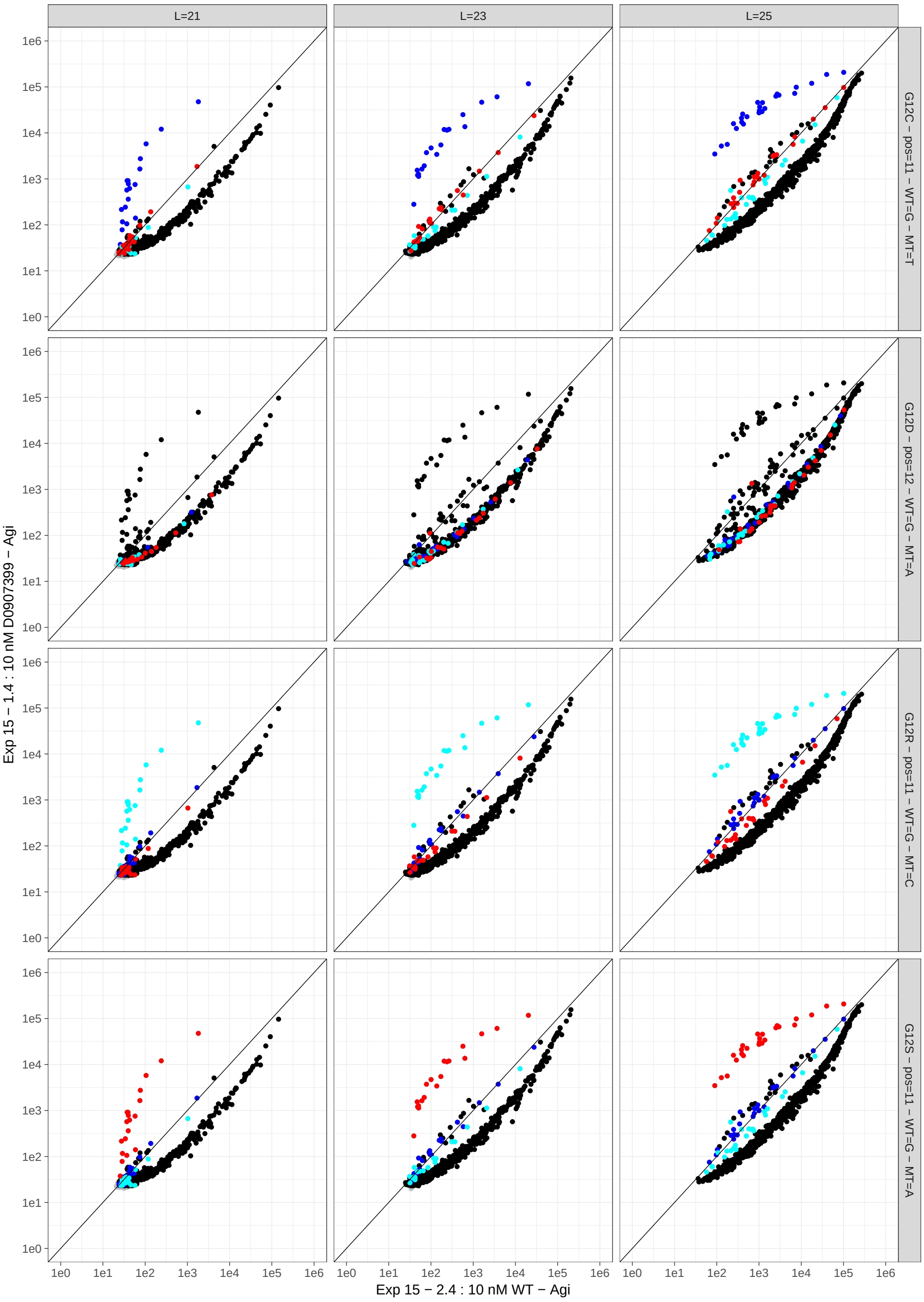

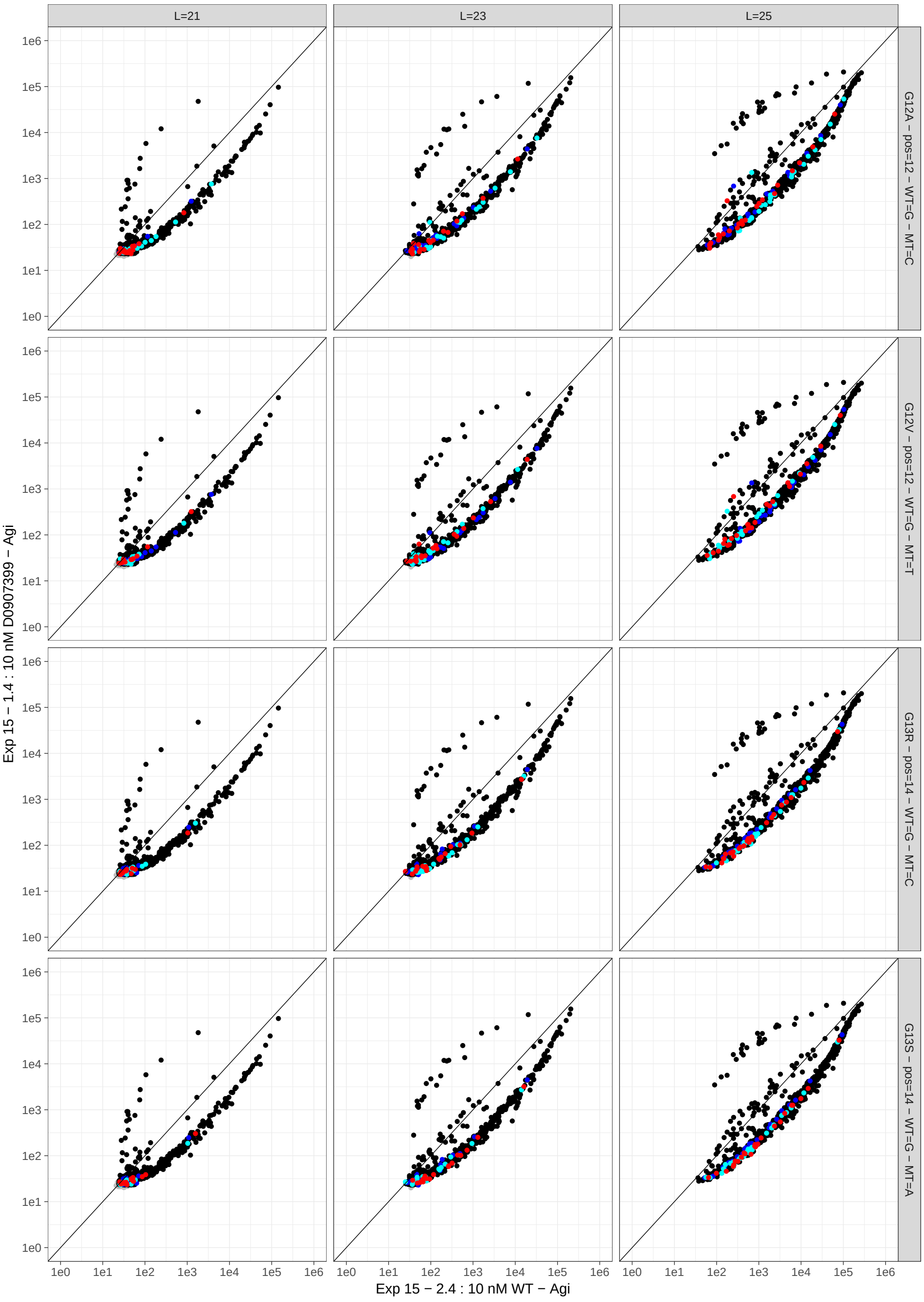

L=21
L=23
L=25
G12A − pos=12 − WT=G − MT=C
G12V − pos=12 − WT=G − MT=T
G13R − pos=14 − WT=G − MT=C
G13S − pos=14 − WT=G − MT=A
Exp 15 − 1.4 : 10 nM D0907399 − Agi
Exp 15 − 2.4 : 10 nM WT − Agi
1e0
1e1
1e2
1e3
1e4
1e5
1e6

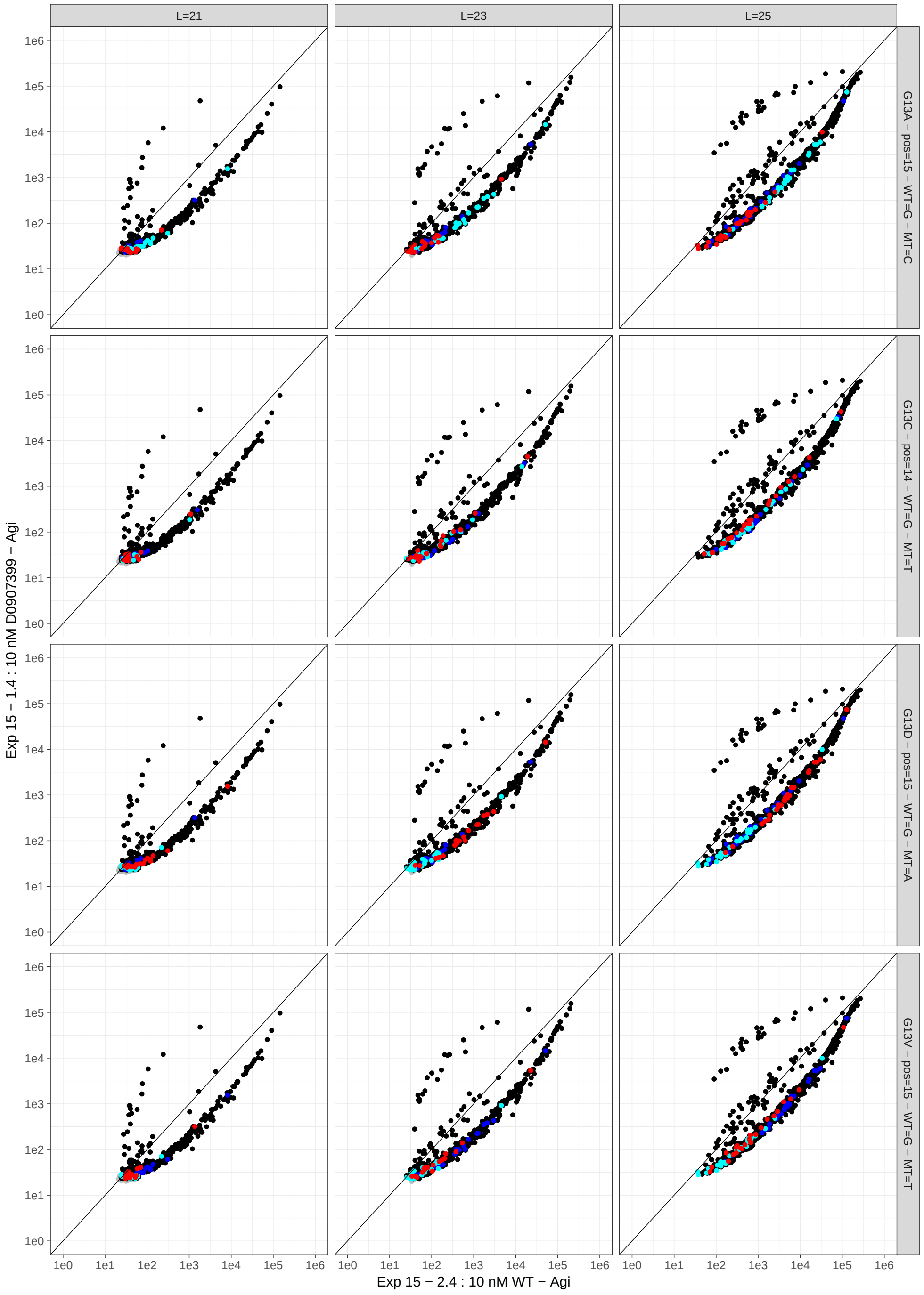
L=21
L=23
L=25
G13A − pos=15 − WT=G − MT=C
G13C − pos=14 − WT=G − MT=T
G13D − pos=15 − WT=G − MT=A
G13V − pos=15 − WT=G − MT=T
Exp 15 − 1.4 : 10 nM D0907399 − Agi
Exp 15 − 2.4 : 10 nM WT − Agi
1e0
1e1
1e2
1e3
1e4
1e5
1e6

## Experiment 24, G12D

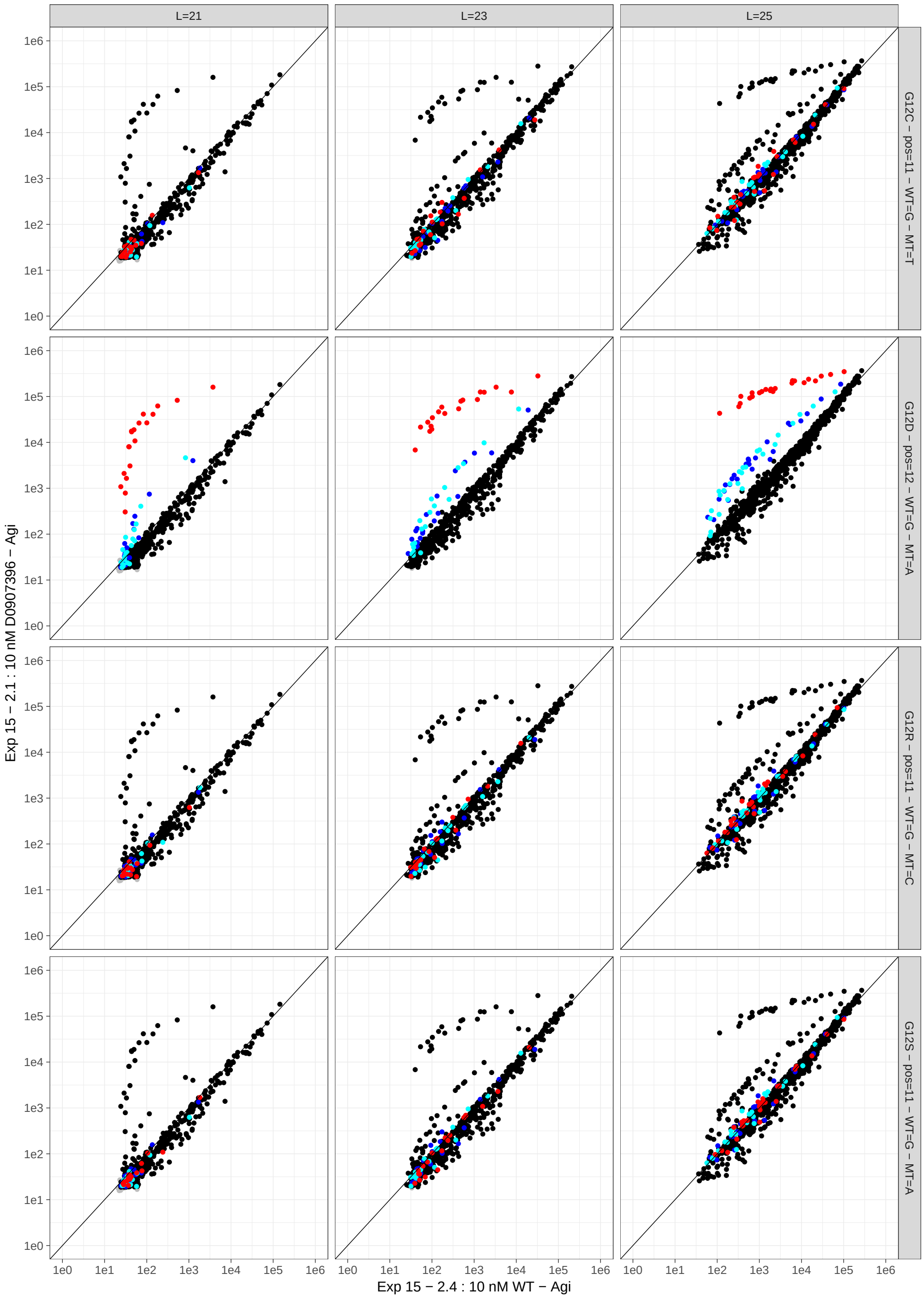

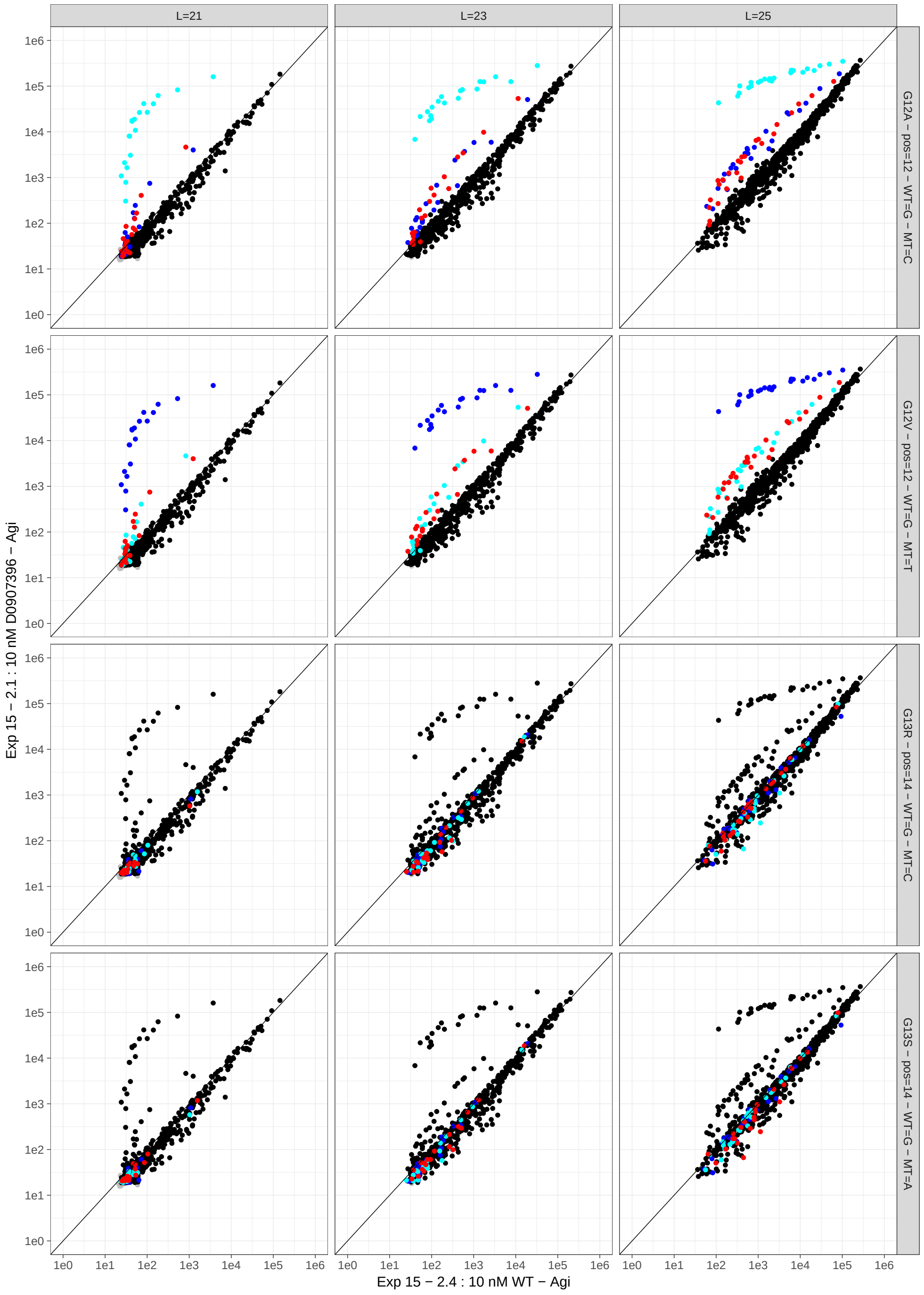

L=21
L=23
L=25
G12A − pos=12 − WT=G − MT=C
G12V − pos=12 − WT=G − MT=T
G13R − pos=14 − WT=G − MT=C
G13S − pos=14 − WT=G − MT=A
1e0
1e1
1e2
1e3
1e4
1e5
1e6
Exp 15 − 2.1 : 10 nM D0907396 − Agi
Exp 15 − 2.4 : 10 nM WT − Agi

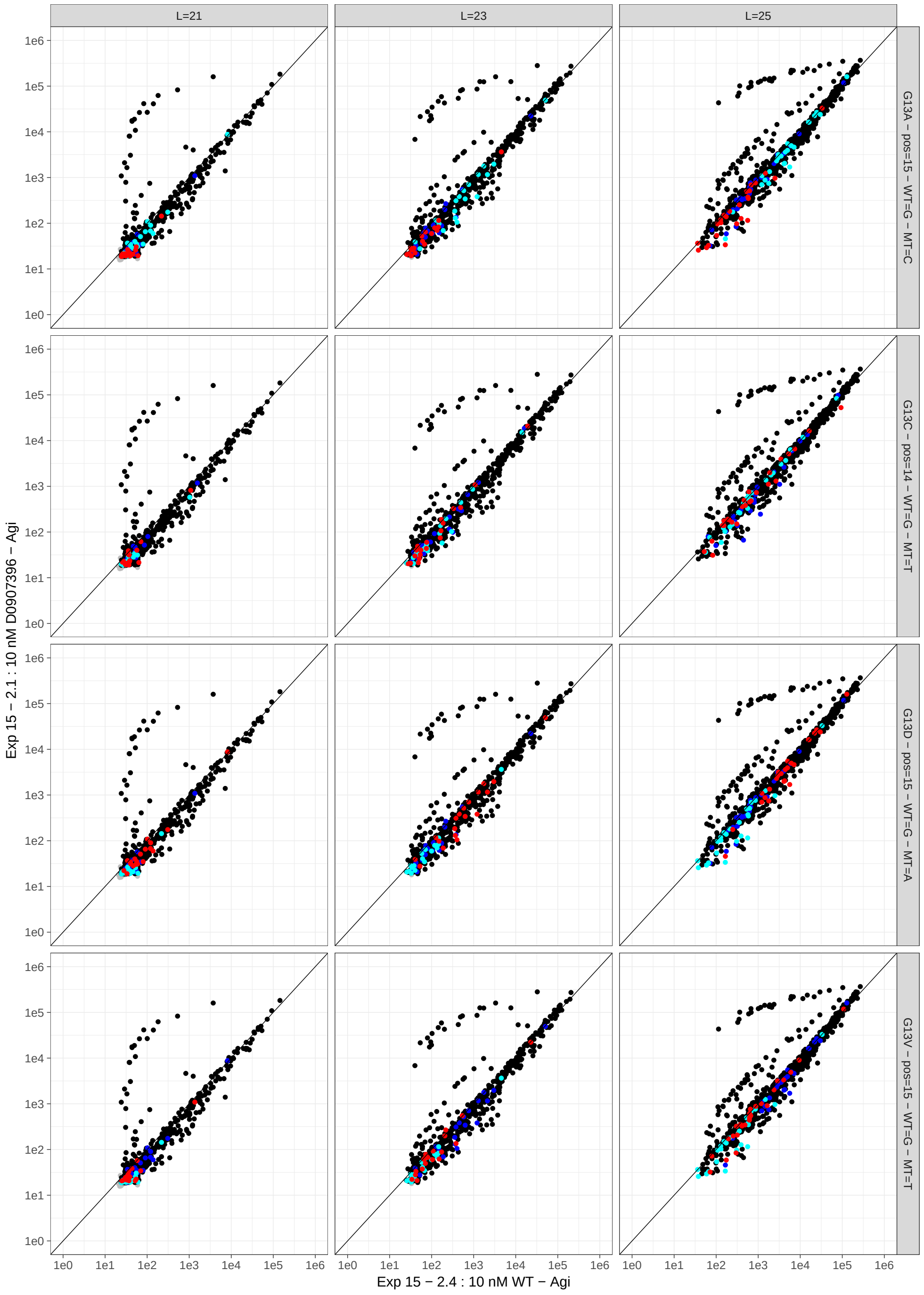

L=21
L=23
L=25
G13A − pos=15 − WT=G − MT=C
G13C − pos=14 − WT=G − MT=T
G13D − pos=15 − WT=G − MT=A
G13V − pos=15 − WT=G − MT=T
1e0
1e1
1e2
1e3
1e4
1e5
1e6
Exp 15 − 2.1 : 10 nM D0907396 − Agi
Exp 15 − 2.4 : 10 nM WT − Agi

# Experiment 25, G13D

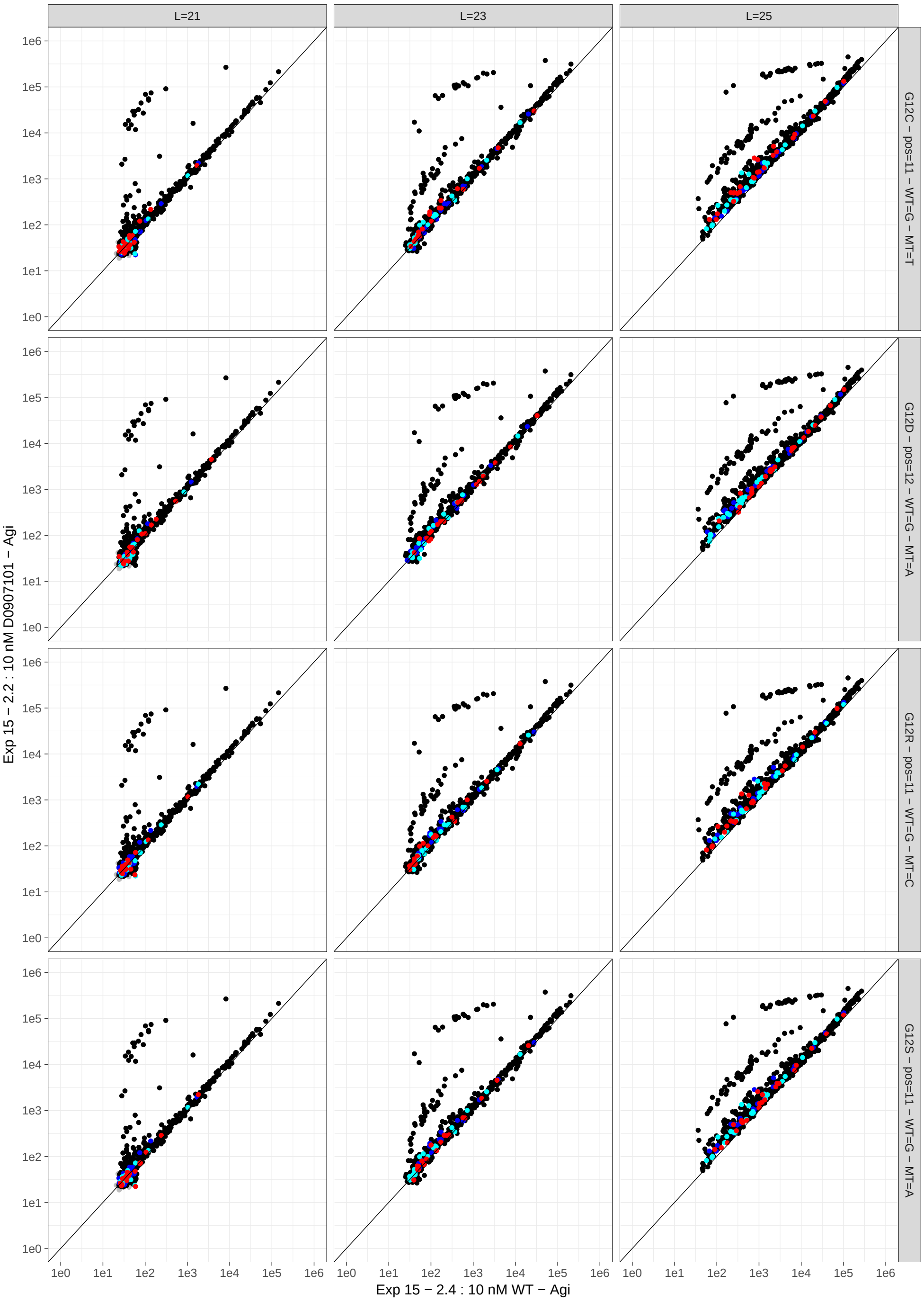

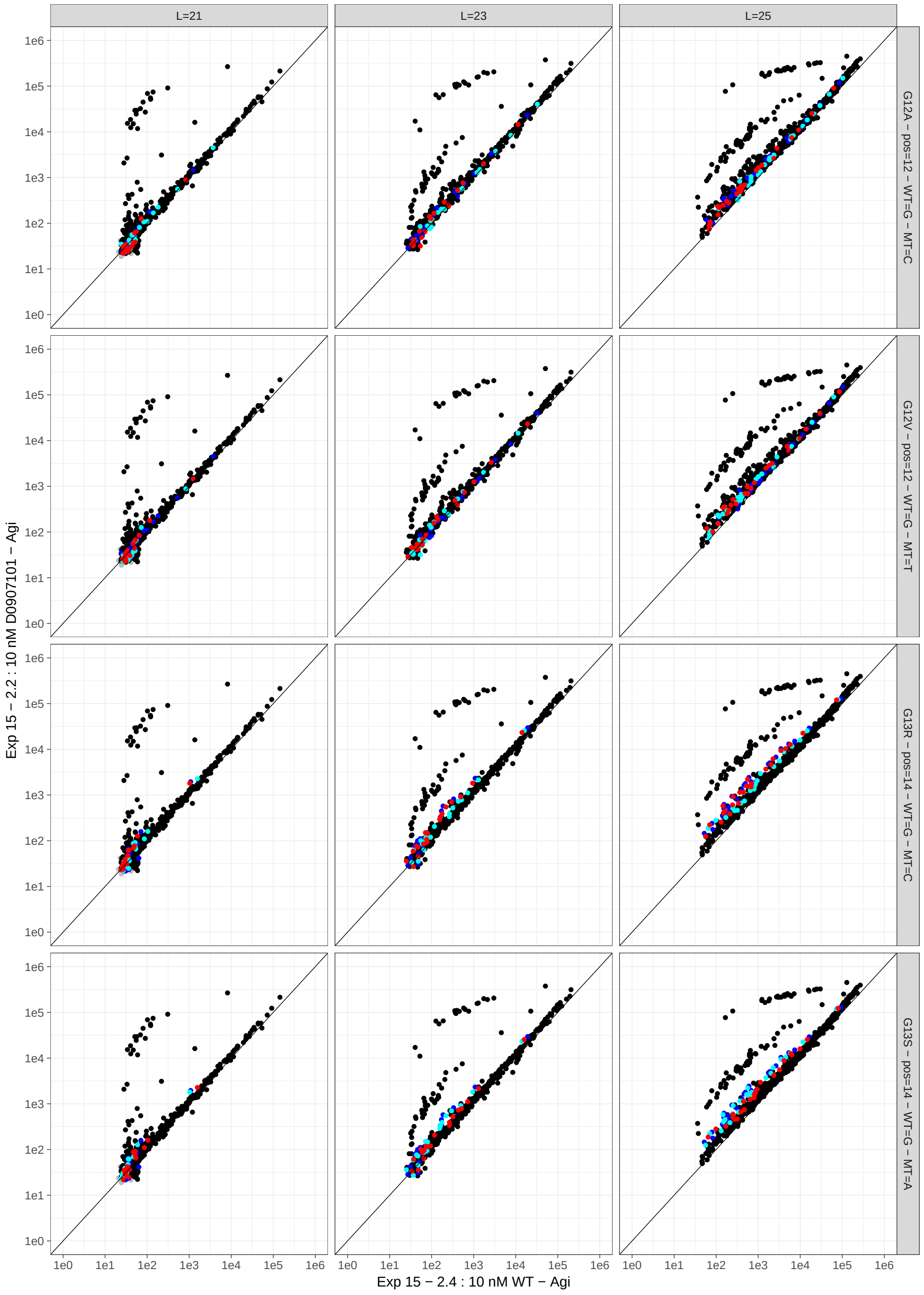

L=21
L=23
L=25
G12A − pos=12 − WT=G − MT=C
G12V − pos=12 − WT=G − MT=T
G13R − pos=14 − WT=G − MT=C
G13S − pos=14 − WT=G − MT=A
1e0
1e1
1e2
1e3
1e4
1e5
1e6
Exp 15 − 2.2 : 10 nM D0907101 − Agi
Exp 15 − 2.4 : 10 nM WT − Agi

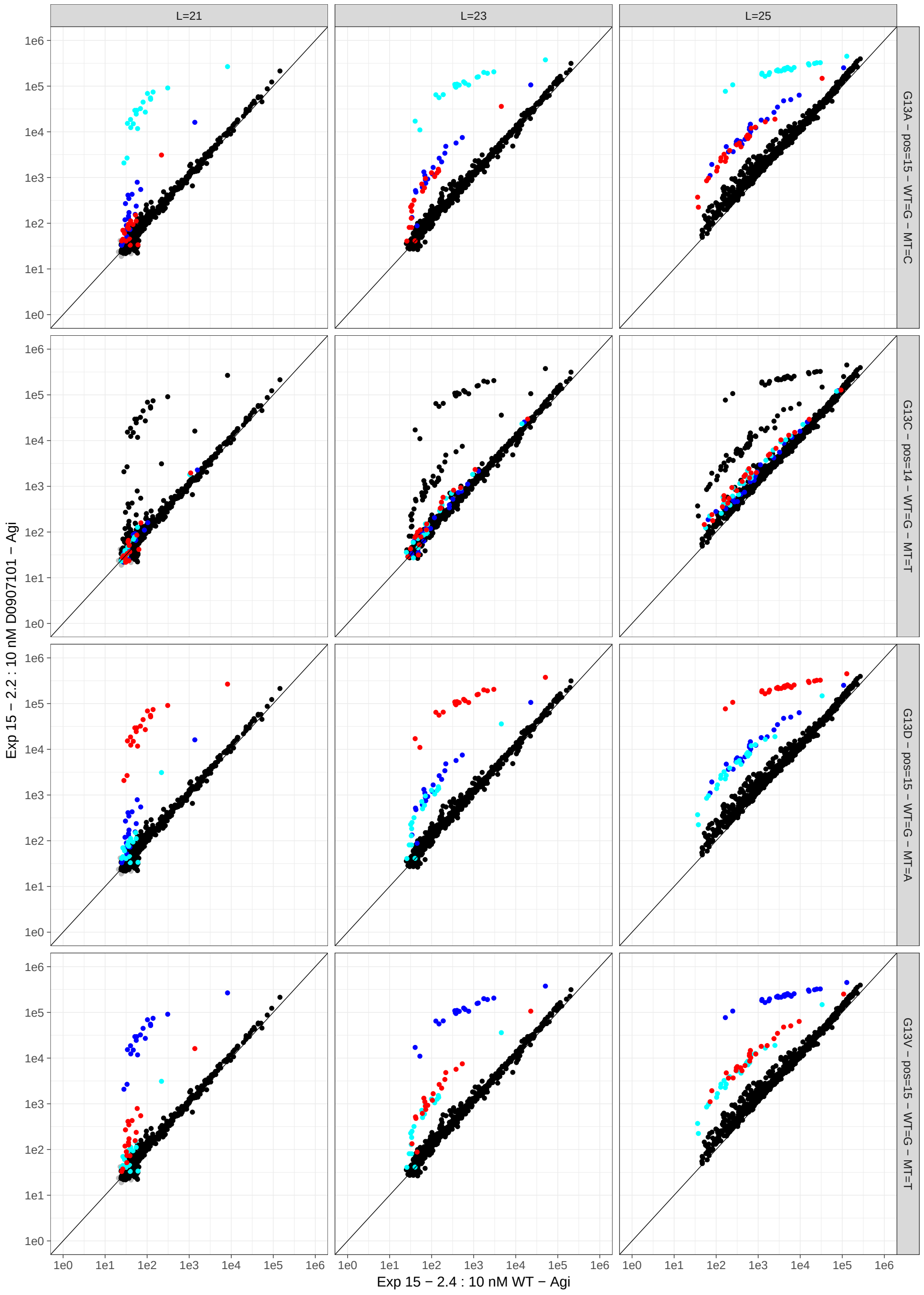

L=21
L=23
L=25
G13A − pos=15 − WT=G − MT=C
G13C − pos=14 − WT=G − MT=T
G13D − pos=15 − WT=G − MT=A
G13V − pos=15 − WT=G − MT=T
Exp 15 − 2.2 : 10 nM D0907101 − Agi
Exp 15 − 2.4 : 10 nM WT − Agi
1e0
1e1
1e2
1e3
1e4
1e5
1e6

# Experiment 26, G12V

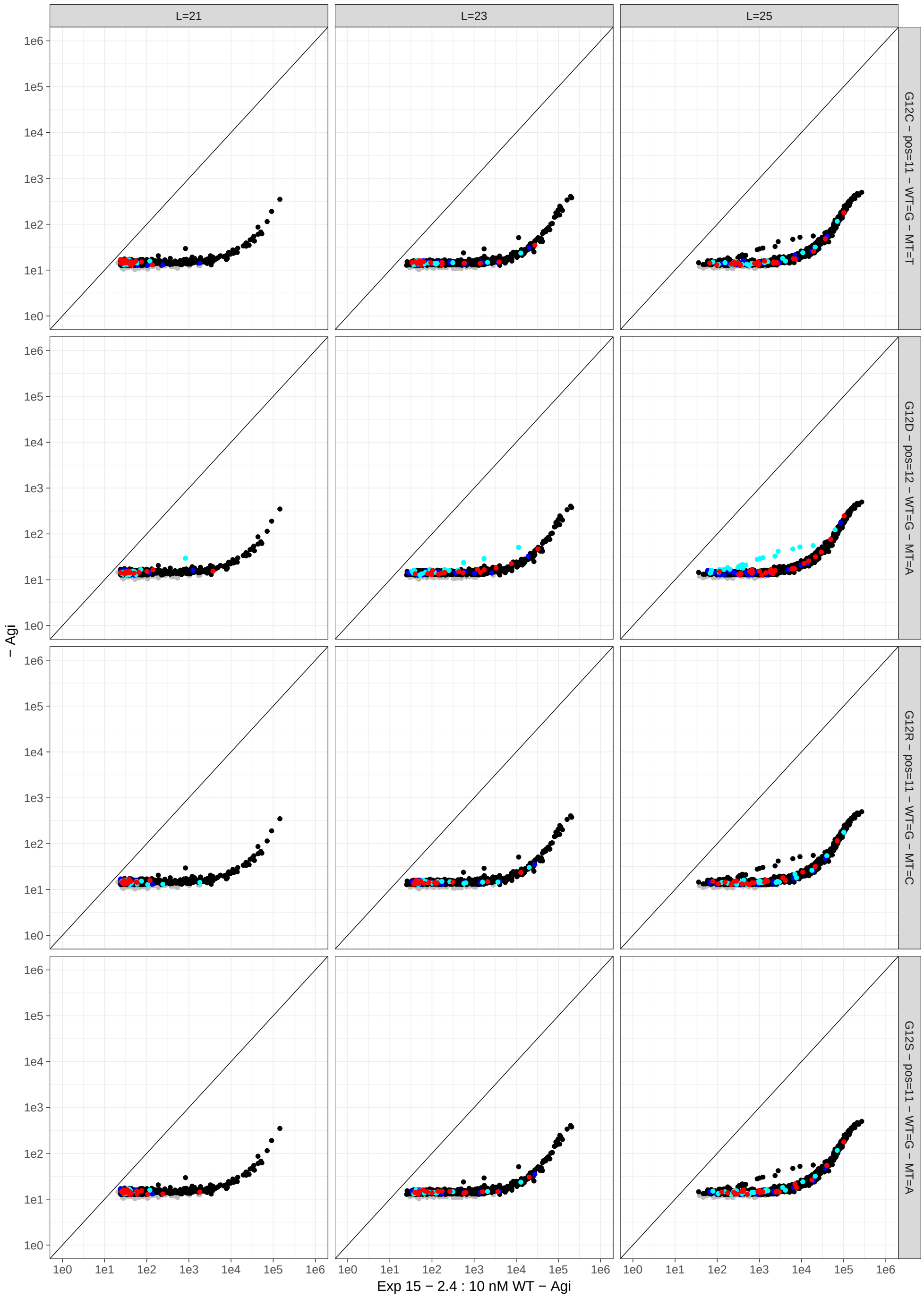

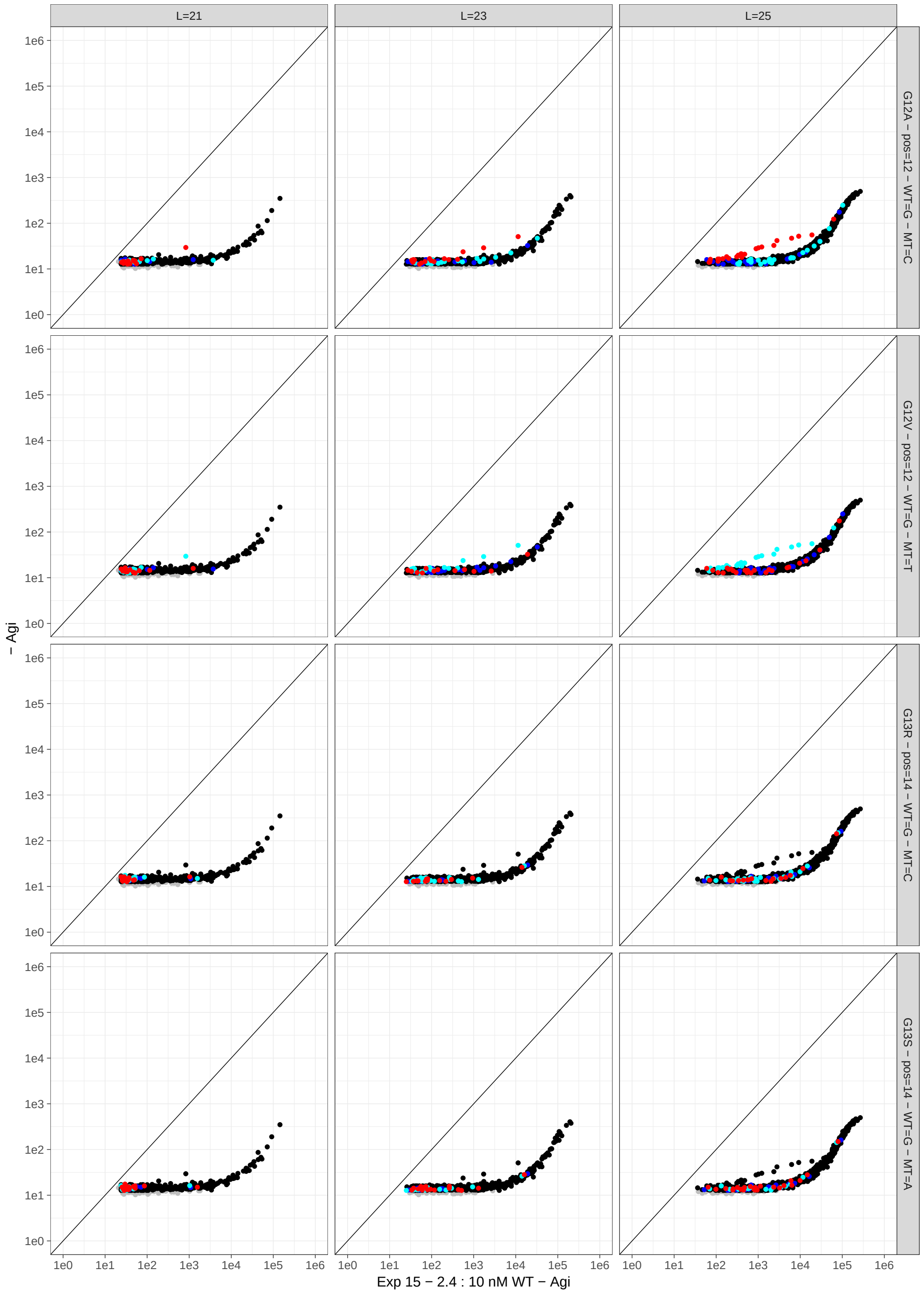
L=21
L=23
L=25
G12A − pos=12 − WT=G − MT=C
G12V − pos=12 − WT=G − MT=T
G13R − pos=14 − WT=G − MT=C
G13S − pos=14 − WT=G − MT=A
1e6
1e5
1e4
1e3
1e2
1e1
1e0
− Agi
Exp 15 − 2.4 : 10 nM WT − Agi

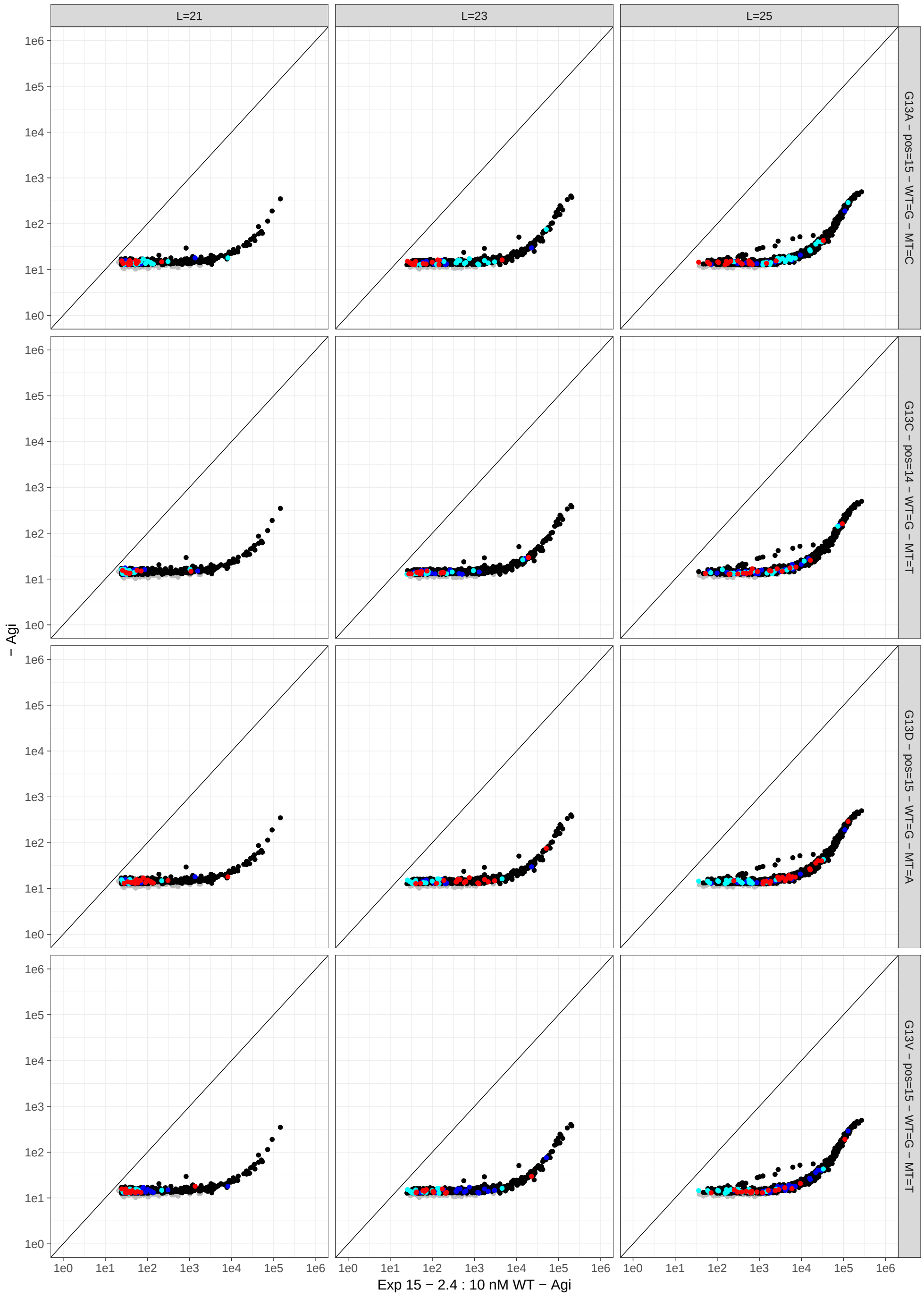

L=21
L=23
L=25
G13A − pos=15 − WT=G − MT=C
G13C − pos=14 − WT=G − MT=T
G13D − pos=15 − WT=G − MT=A
G13V − pos=15 − WT=G − MT=T
1e6
1e5
1e4
1e3
1e2
1e1
1e0
− Agi
Exp 15 − 2.4 : 10 nM WT − Agi

# Experiment 27, WT

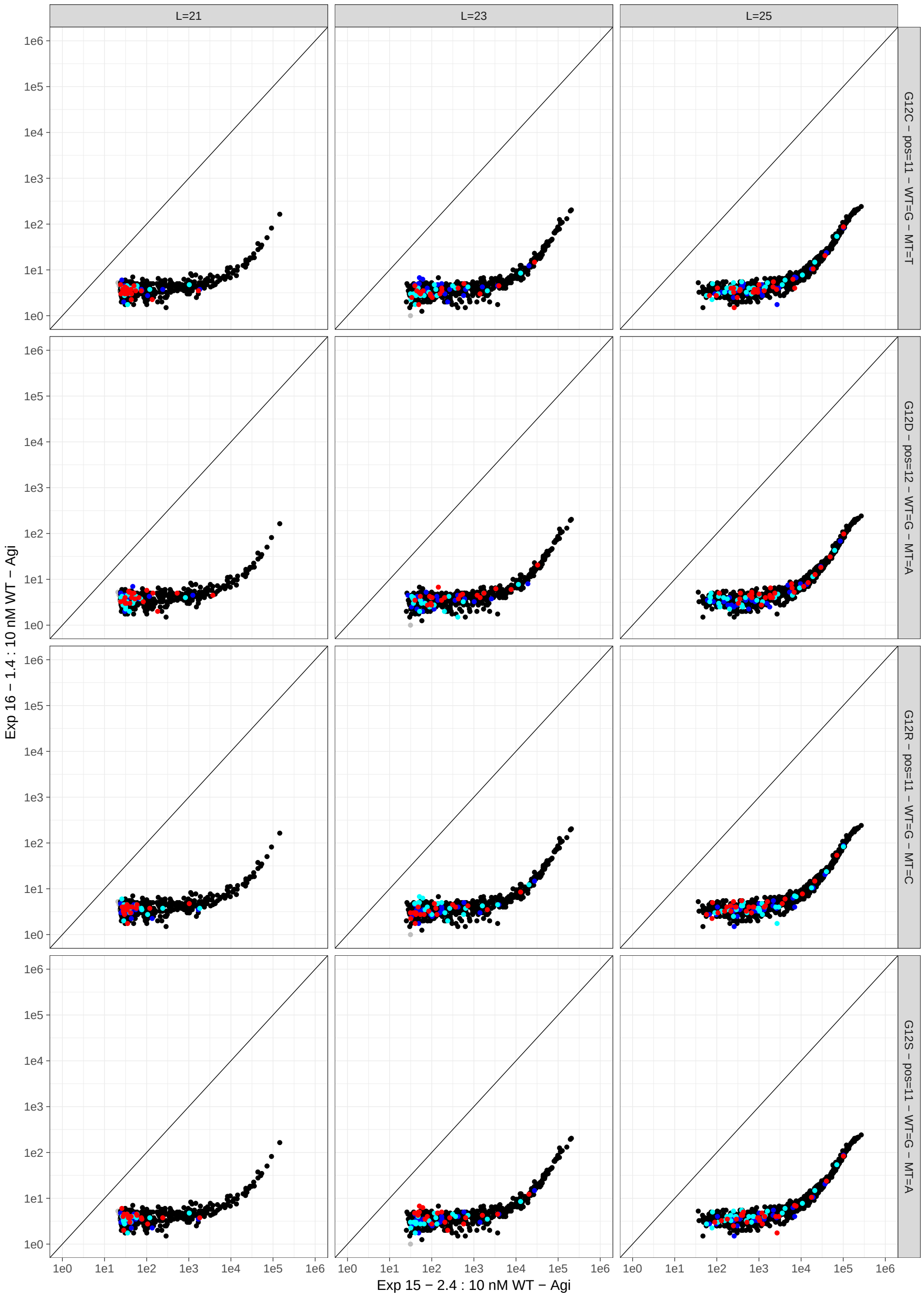

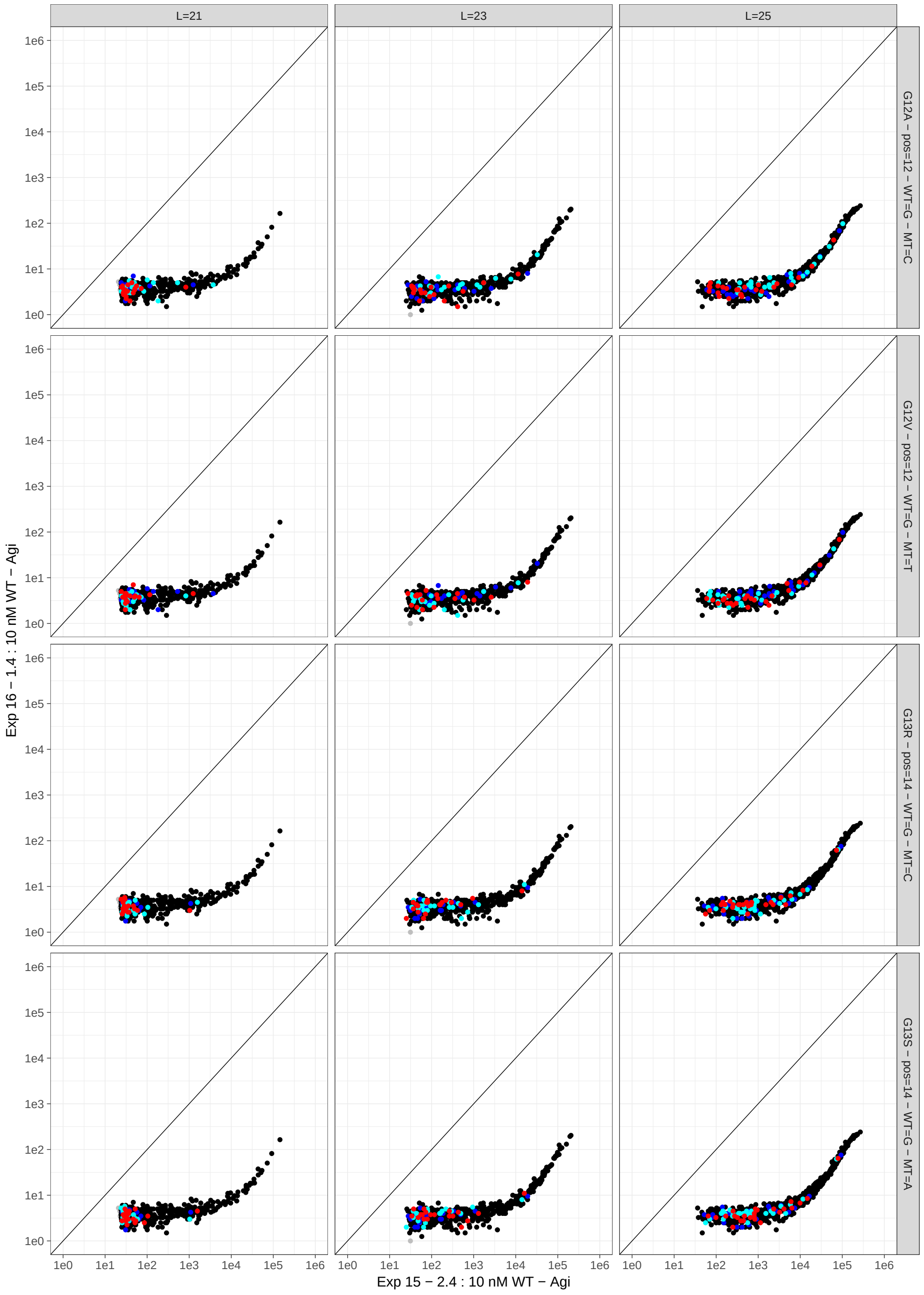

L=21
L=23
L=25
G12A − pos=12 − WT=G − MT=C
G12V − pos=12 − WT=G − MT=T
G13R − pos=14 − WT=G − MT=C
G13S − pos=14 − WT=G − MT=A
1e6
1e5
1e4
1e3
1e2
1e1
1e0
Exp 16 − 1.4 : 10 nM WT − Agi
Exp 15 − 2.4 : 10 nM WT − Agi

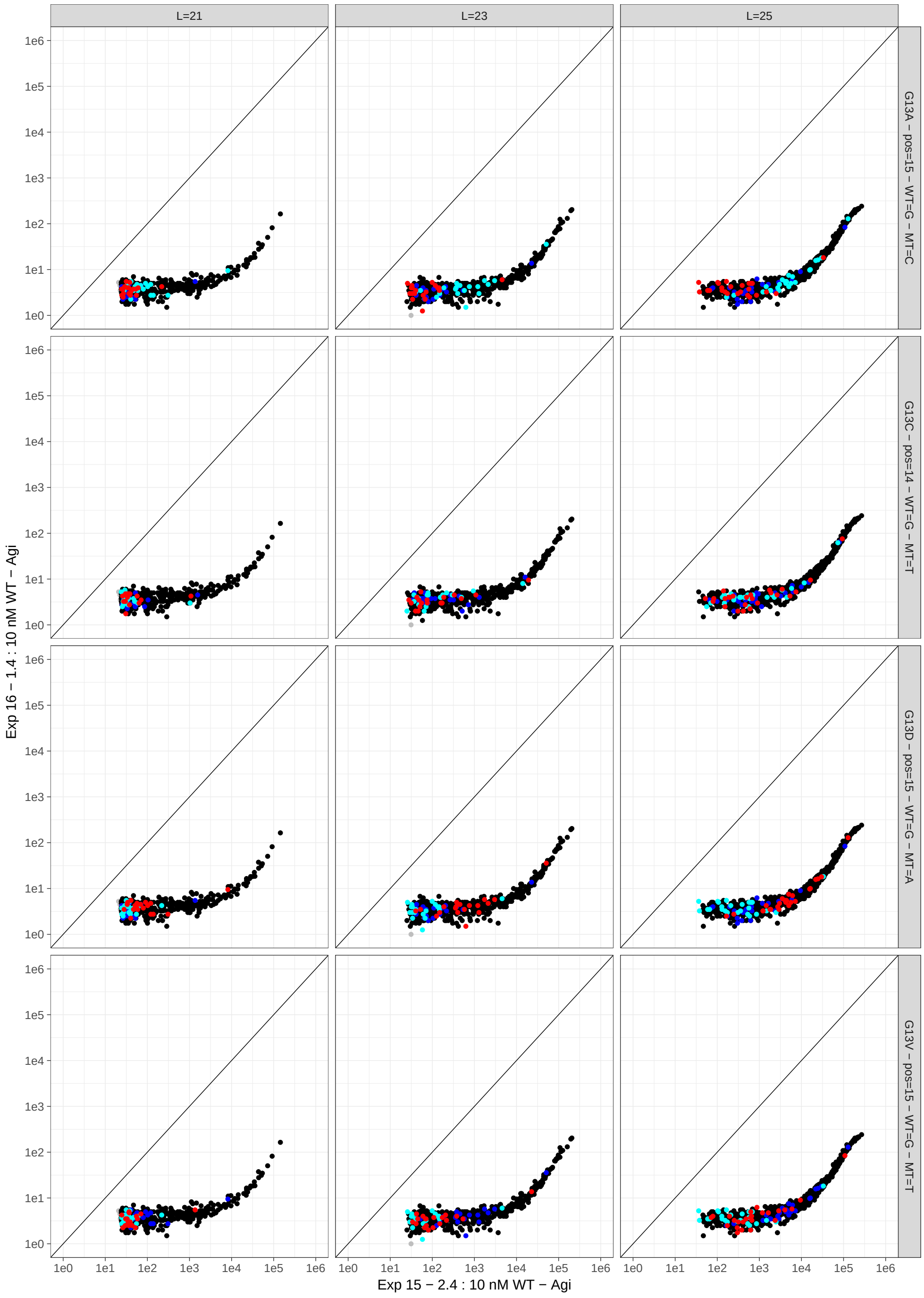
L=21
L=23
L=25
G13A − pos=15 − WT=G − MT=C
G13C − pos=14 − WT=G − MT=T
G13D − pos=15 − WT=G − MT=A
G13V − pos=15 − WT=G − MT=T
1e6
1e5
1e4
1e3
1e2
1e1
1e0
Exp 16 − 1.4 : 10 nM WT − Agi
Exp 15 − 2.4 : 10 nM WT − Agi
