# Supplementary material for: Thermodynamic framework to assess low abundance DNA mutation detection by hybridization
Source: PLoS One. 2017 May 25;12(5):e0177384. doi: 10.1371/journal.pone.0177384 (PMC5444680; doi:10.1371/journal.pone.0177384)
Supplement: S1 Table — (PDF) [file pone.0177384.s002.pdf]

Table S1. Statistical results of experiment set 1: concentration range experiments with gBlocks samples (mutation 10C→G).

nr.	$\mathbf{c_{mut}/c_{total}}$	pos-9	pos-10	pos-12	pos-13
1.	10%	0.0 (0.5)	**44** (5.8)	0.0 (0.2)	0.1 (1.1)
2.	4.0%	0.0 (0.2)	**46** (4.8)	0.2 (0.1)	1.5 (0.1)
3.	1.6%	0.2 (0.1)	**46** (3.7)	0.8 (0.1)	2.7 (0.0)
4.	0.64%	0.3 (0.1)	**46** (2.7)	1.1 (0.0)	5.4 (0.0)
5.	0.26%	1.5 (0.1)	**45** (2.0)	1.3 (0.1)	0.8 (0.0)
6.	0.10%	0.3 (0.1)	**45** (1.4)	2.7 (0.0)	1.9 (0.0)

The first column shows the relative concentration of mutant 10C→G (G12A in amino acid notation). Per sample (row) and per nucleotide position (column) the p' (ρ) values are shown for the most significant mutation hypothesis (out of three possible mutations). The highest p'-values that lead to the identification of the experimental nucleotide mutation in each gBlock sample are indicated in bold.
