# Supplementary material for: Thermodynamic framework to assess low abundance DNA mutation detection by hybridization
Source: PLoS One. 2017 May 25;12(5):e0177384. doi: 10.1371/journal.pone.0177384 (PMC5444680; doi:10.1371/journal.pone.0177384)
Supplement: S3 Table — (PDF) [file pone.0177384.s004.pdf]

Table S3. Statistical results of experiment set 3: blinded clinical samples

nr.	NT notation	AA notation	pos-9	pos-10	pos-12	pos-13
20.	13C→T	G13D	0.5 (0.2)	0.4 (0.2)	1.2 (0.2)	**70** (4.9)
21.	10C→G	G12A	0.1 (0.3)	**47** (6.1)	0.0 (0.5)	0.0 (0.4)
22.	10C→A	G12V	2.3 (0.1)	**64** (5.6)	0.3 (0.2)	0.2 (0.2)
23.	9C→T	G12S	**70** (5.0)	0.6 (0.3)	0.1 (0.5)	0.1 (0.4)
24.	10C→T	G12D	5.1 (0.0)	**69** (5.8)	0.0 (0.5)	0.0 (0.5)
25.	13C→T	G13D	0.3 (0.4)	0.0 (0.4)	8.6 (0.2)	**61** (5.2)
26.	10C→A	G12V	3.0 (0.0)	**64** (6.5)	1.2 (0.2)	0.4 (0.4)
27.	WT	WT	1.6 (0.0)	0.8 (0.0)	3.6 (0.1)	6.6 (0.2)

The tested clinical samples are listed with mutations in nucleotide (NT) and amino acid (AA) notation, except for the WT control (experiment 27). Per sample (row) and per nucleotide position (column) the p' (ρ) values are shown for the most significant mutation hypothesis (out of three possible mutations). The highest p'-values that lead to the identification of the experimental nucleotide mutation in each clinical sample are indicated in bold.
